# Highly Active Iminopyridyl Iron-Based Catalysts for the Polymerization of Isoprene

**DOI:** 10.3390/molecules24173024

**Published:** 2019-08-21

**Authors:** Obaid H. Hashmi, Yohan Champouret, Marc Visseaux

**Affiliations:** UMR 8181–UCCS–Unité de Catalyse et de Chimie du Solide, ENSCL, Centrale Lille, University of Artois, University of Lille, CNRS, F-59000 Lille, France

**Keywords:** iminopyridyl ligand, iron (II)-based catalyst, coordination-insertion polymerization, isoprene polymerization

## Abstract

A series of iminopyridyl-based ligands, 6-[(Ar)N=C(R)]-2-C_6_H_5_N [(Ar = 2,6-Me_2_-C_6_H_3_, R = Me (**L1**); Ar = 2,6-*^i^*Pr_2_-C_6_H_3_, R = Me (**L2**); Ar = 2,6-Me_2_-C_6_H_3_, R = H (**L3**); Ar = 2,6-*^i^*Pr_2_-C_6_H_3_, R = H (**L4**); Ar = 3,5-(CF_3_)_2_-C_6_H_3_, R = Me (**L5**); Ar = C_6_F_5_, R = Me (**L6**)], and their corresponding iron (II) complexes were developed to investigate their application in the controlled coordinative polymerization of isoprene. The modulation of steric and electronic properties within this family of ligands/pre-catalysts has shown to influence the stereo-selectivity and activity of the polymerization of isoprene after activation. Upon activation with various co-catalysts such as Al*^i^*Bu_3_/[Ph_3_C][B(C_6_F_5_)_4_], AlEt_3_/[Ph_3_C][B(C_6_F_5_)_4_] or MAO, the resulting catalysts produced polyisoprenes with an excellent conversion (>99% of 500–5000 equiv.) within less than 1 h (TOF > 500 h^−1^) and having a variety of stereo-/regio-regularities. The presence of electron-donating and withdrawing groups drastically impacted the activity and the stereoselectivity of the catalysts during the course of the polymerization of isoprene. When activated with Al*^i^*Bu_3_/[Ph_3_C][B(C_6_F_5_)_4_], the complexes {6-[(2,6-Me_2_-C_6_H_3_)N=C(Me)]-2-C_6_H_5_N}FeCl_2_ (**C1**) and {6-[(2,6-*^i^*Pr_2_-C_6_H_3_)N=C(Me)]-2-C_6_H_5_N}FeCl_2_ (**C2**) exhibited moderate *trans*-1,4 selectivity (>67%) while the iron-based systems bearing related aldiminopyridyl ligands {6-[(2,6-Me_2_-C_6_H_3_)N=C(H)]-2-C_6_H_5_N}FeCl_2_ (**C3**) and {6-[(2,6-*^i^*Pr_2_-C_6_H_3_)N=C(H)]-2-C_6_H_5_N}FeCl_2_ (**C4**) were found to afford significant *cis*-1,4 selectivity at low temperature (>86% at −40 °C). On the other hand, the ternary {6-[(3,5-(CF_3_)_2_-C_6_H_3_)N=C(Me)]-2-C_6_H_5_N}FeCl_2_ (**C5**) or {6-[(C_6_F_5_)N=C(Me)]-2-C_6_H_5_N}FeCl_2_ (**C6**)/Al*^i^*Bu_3_/[Ph_3_C][B(C_6_F_5_)_4_] catalytic combinations showed exceptional activity for the polymerization of isoprene (TOF > 1,000,000 h^−1^), albeit providing less stereoselectivity.

## 1. Introduction

Since the initial report twenty years ago on the high catalytic activity of the bis(imino)pyridyl-iron/MAO system for the polymerization of ethylene [1,2], the field of iron-catalyzed polymerization reactions has seen a substantial surge of interest for the preparation of a variety of polymeric materials [3,4]. Particular attention has been paid to the development of homogeneous single-site polymerization catalysts, as these systems have, to some extent, provided precise control over molecular weights, molecular weight distribution and stereo-regularity of the polymer chains in comparison with heterogeneous catalysts [5,6,7]. In this context, the research community has been motivated to ingeniously design discrete iron-based pre-catalysts for the coordination-insertion polymerization of not only olefins and related monomers, but also vinyl and cyclic polar monomers [8].

**Figure 1:**
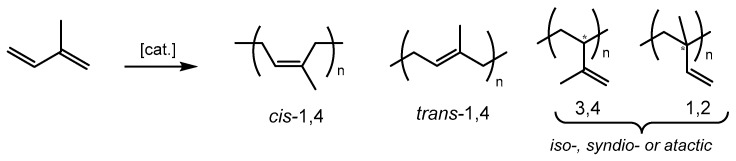
Microstructure contents of polyisoprene.

Among the family of synthetic polymers, polyisoprene is currently receiving special attention for its use in a wide range of applications in the rubber industry, e.g., tire manufacturing, medical devices and others [9,10,11,12,13]. The coordination-insertion polymerization of isoprene produces four types of unit distributions such as *cis*-1,4, *trans*-1,4, 3,4 and 1,2-vinyl arrangements (Figure 1), the amount and type of sequences dictating the resulting thermal, mechanical and physical properties of the polymer. To date, single-site catalysts for the polymerization of 1,3-dienes are mainly based on transition (Ti, Co, Ni) and rare earth (Nd) metal-based systems, producing concomitantly, to a certain degree, high molecular mass polymers with controlled microstructures [14,15,16,17,18,19]. On the other hand, single-site iron-based catalysis for isoprene polymerization has received less attention, whereas this metal is easily accessible, inexpensive and essentially non-toxic compared to its counterparts [20].

**Figure 2:**
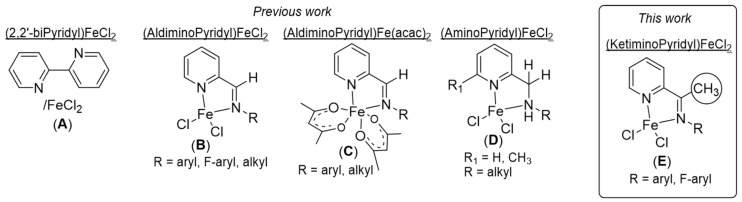
Iron-based complexes bearing bidentate pyridyl-based ligands for the polymerization of isoprene. (A). Ref [22]; (B). Ref [24,25,26]; (C). Ref [27], (D). Ref [28].

Most iron-active systems are mainly supported by bidentate and tridentate aromatic nitrogen ligands [3,18] the former system displaying the most promising performances in terms of activity and selectivity as it offers an additional coordination site to accommodate the isoprene monomer when compared to the latter [8]. In 2002, Ricci et al. showed that the (2,2′-bipyridyl)FeEt_2_/MAO catalytic combination [21], or a mixture of 2,2′-bipyridine, FeCl_2_ and MAO [22] (Figure 2(A)), was able to produce polyisoprene with an exceptionally high efficiency (TOF = 800,000 h^−1^) and poor 3,4-regio-selectivity (*cis*-1,4/3,4 = 33/67). Density functional calculation of the insertion pathway confirmed that the 3,4-regio-selectivity was preferred over the 1,4-insertion [23].

Later, the Ritter’s group reported the *quasi* selective polymerization of isoprene using the (aldiminopyridyl)FeCl_2_ complexes (Figure 2(B)) associated with trialkyl aluminum and [Ph_3_C][B(C_6_F_5_)_4_] [24]. A reversal of the selectivity was achieved by modifying the nature of the substituent on the imino group, the *N*-aryl group (aryl = supermesityl) favoring the *cis*-1,4-selectivity while the *trans-*1,4-selectivity was promoted with an *N*-alkyl substituent (alkyl = tert-octyl). On the other hand, the same sets of complexes were independently evaluated for the polymerization of isoprene by the group of Chen [25] and the group of Wang [26] in the presence of MAO. Surprisingly, the system bearing *N*-alkyl substituent afforded opposite microstructures, respectively, whereas the complex with *N*-aryl group exhibited a slight *cis*-1,4 selectivity with a moderate amount of 3,4 units, relatively, similar to that found upon activation with trialkyl aluminum and [Ph_3_C][B(C_6_F_5_)_4_]. Moreover, for the same catalytic systems and under the same experimental conditions, different activities were found by these two research groups, enabling almost complete conversion of the isoprene in one case, while in the other, only 10 to 20% conversion was reached. In addition, Wang studied the polymerization of isoprene with aldiminopyridyl iron-based system bearing fluorinated *N*-aryl substituent (Figure 2(B)) [26], which was activated either by MAO alone or by a combination of MAO and [Ph_3_C][B(C_6_F_5_)_4_]. The obtained polyisoprene exhibited an equal content mixture of *cis*-1,4 and 3,4 units for the former while high *trans*-1,4 selectivity (>95%) was found in the presence of MAO/[Ph_3_C][B(C_6_F_5_)_4_].

Subsequently, Wang and collaborators described the effect of the auxiliary ligand in the iron-based iminopyridyl complexes by replacing both chloride anions by two acetylacetonato (acac) groups (Figure 2(C)) [27]. In the presence of an excess of MAO, the polyisoprene prepared with the (iminopyridyl)Fe(acac)_2_ system bearing *N*-*tert*-octyl substituents showed a good *trans*-1,4 stereo-regularity (>87%) with an activity five times higher than that of the related chlorinated iron complex. Furthermore, the *N*-aryl counterpart displayed high catalytic activity (TOF > 12,000 h^−1^) but poor selectivity, emphasizing the influence of the resulting counter anion. 

In the past few months, the same research group disclosed the polymerization of isoprene using a series of iron-based complexes supported by aminopyridine ligands (Figure 2(D)) [28]. Upon activation with an excess of MAO, these pre-catalysts provided a wide range of polyisoprene microstructures with, in most cases, high content of 3,4 units. The performance of the catalytic systems was shown to depend on the nature of the substituent carried by the pyridine ring and the *N*-amine group of the aminopyridyl ligand, with the complex bearing R = CH(Ph)_2_ and R_1_ = H showing a very high activity of TOF > 28,000 h^−1^. In parallel, it is worth mentioning that related iron-based pre-catalysts supported by iminoimidazole [29] or 2-(*N*-arylcarboximidoylchloride)-quinoline [30] were also assessed for the polymerization of isoprene.

Overall, these results highlight the importance of the structure/properties of the iron pre-catalyst systems bearing iminopyridyl or aminopyridyl ligand as well as their mode of activation for controlling the polymerization of isoprene. At this stage, it is difficult to draw any rational conclusions regarding the influence of the electronic and steric properties of the ancillary ligand along with the nature of the cocatalyst on the selectivity and activity of the polymerization process.

Herein, we wish to contribute to this field by reporting the polymerization of isoprene with iron complexes supported by various iminopyridyl ligands with a methyl substituent on the carbon of the imino group (Figure 2(E)). To our knowledge, the use of ketiminopyridyl-iron pre-catalysts for the polymerization of isoprene was only studied for the embedment of iron nanoparticles in polyisoprene within the cavity of carbon nanotubes [31]. In any case, no details on the selectivity and activity of the polymerization were described. In the present article, the iron-catalyzed polymerization of isoprene was investigated through the modification of the iminopyridyl ligand framework using two complexes bearing *N*-aryl substituted ketiminopyridyl, which were compared with their aldiminopyridyl counterparts, as well as fluorinated *N*-aryl substituted ketiminopyridyl ligands. The control of the polymerization of isoprene using these complexes was explored under different experimental conditions, in particular by changing the nature of the co-catalyst, the temperature of polymerization and the isoprene/catalyst ratio.

## 2. Results and Discussion

### 2.1. Synthesis of Pro-Ligands **(*L1***–***L6***) and Their Corresponding Iron-Based Complexes (***C1***–***C6***)

**Scheme 1:**
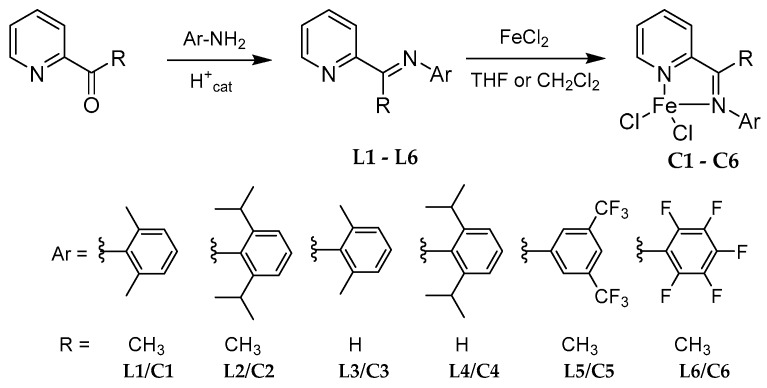
Synthesis of the ligands and iron (II) complexes.

All the iminopyridyl pro-ligands and their corresponding iron complexes have been prepared following the synthetic strategy as shown in Scheme 1. The known **L1**–**L5** pro-ligands were synthesized via acid-catalyzed condensation reaction between 2-acetylpyridine (for the ketiminopyridyl proligands **L1**, **L2** and **L5**) or 2-pyridinecarboxaldehyde (for the aldiminopyridyl proligands **L3** and **L4**) with their corresponding anilines in methanol/toluene, and their respective ^1^H-NMR spectra were consistent with the reported data [32,33,34,35,36] (details are given in the Experimental section, ^1^H-NMR spectra are displayed in Figures S4–S8). Following a similar synthetic methodology, the new **L6** pro-ligand was obtained in poor yield (*ca* 10%) from reaction of 2-acetylpyridine and 2,3,4,5,6-pentafluoroaniline in toluene at high temperature. **L6** was characterized by ^1^H-, ^13^C-, ^19^F-NMR and elemental analysis (see Experimental section and Supplementary Materials, Figures S1–S3). The IR spectra of all ligands revealed the characteristic C=N vibration band in the range of 1633–1649 cm^−1^, with **L5** and **L6** showing the highest stretching frequency, as expected, due to the presence of fluorinated groups. 

Subsequently, the complexation of pro-ligands **L1**–**L4** with anhydrous FeCl_2_ in THF or CH_2_Cl_2_ afforded their related iminopyridyl-based iron complexes **C1**–**C4** in moderate to good yields (53–82%) as previously described in the literature [34,35,37,38,39]. The new iron complexes **C5** and **C6** were synthesized in the same way as their congeners in good yields (79–83%). All complexes were characterized by ^1^H-NMR (the spectra are displayed in the Supplementary Materials, Figures S9–S15) and elemental analyses were also performed to confirm the molecular formula of the new iron complexes **C5** and **C6** (Experimental section). The IR spectra of the resulting complexes displayed a C=N stretching frequency between 1589–1597 cm^−1^; values lower than those identified for their related free pro-ligand due to the coordination of the N atom of the imino group on the metal center. Furthermore, the highest wavenumber differences between pro-ligands and complexes were predictably observed for those bearing electron withdrawing fluorinated substituents (Δυ_(C=N)_ = 52 cm^−1^ for **L5**/**C5** and **L6**/**C6** vs. Δυ_(C=N)_ = 40–46 cm^−1^ for **L1**/**C1**–**L4**/**C4**), indicating, as expected, a more important decrease in electron density of the iron center for **C5**–**C6** compared to **C1**–**C4**.

### 2.2. Polymerization of Isoprene with Iron-Based Complexes ***C1–C6***

The polymerization of isoprene was assessed at room temperature using pre-catalysts **C1**–**C6** under different modes of activation. Various reagents such as triisobutylaluminium (Al^i^Bu_3_) or triethylaluminium (AlEt_3_) combined with trityltetrakis(pentafluorophenyl)borate [CPh_3_][B(C_6_F_5_)_4_] or an excess of MAO were employed as co-catalysts. The results of the polymerization of isoprene using the ternary **C1**–**C6**/Al^i^Bu_3_/[Ph_3_C][B(C_6_F_5_)_4_] catalytic systems are presented in Table 1 and will be discussed here. The polymerization results using **C1**–**C6**/AlEt_3_/[Ph_3_C][B(C_6_F_5_)_4_] and **C1**–**C6**/MAO, which are marginally different from the results presented in Table 1, are depicted in the supporting materials (Tables S1 and S2, respectively) and will be mentioned at the end of this section.

Regardless of the nature of the co-catalysts {Al^i^Bu_3_/[Ph_3_C][B(C_6_F_5_)_4_], AlEt_3_/[Ph_3_C][B(C_6_F_5_)_4_] or MAO}, all the **C1** – **C6** complexes proved to be highly active for the polymerization of isoprene with complete conversion of 500 equiv. of monomer per iron catalyst within 1 h (TOF > 500 h^−1^). These results compare well with related iron-based catalysts available in the literature [8]. 

Analysis of the resulting polyisoprenes (PIs) by size-exclusion chromatography (SEC) revealed that the ternary **C1** and **C2**/Al^i^Bu_3_/[Ph_3_C][B(C_6_F_5_)_4_] catalytic systems (ratio of 1/10/1, respectively) gave *M*_n_ slightly higher than the theoretical value with monomodal curves displaying narrow dispersity (Table 1, entries 1 and 2, respectively). In turn, catalysts resulting from complexes **C3** and **C4** afford *M*_n_ values up to three times higher than expected, which speaks in favor of a slow initiation process under these conditions. The polymerization of isoprene was even less controlled with complex **C5** and **C6** but, interestingly, very high *M*_n_ polyisoprenes could be produced (*M*_n_ > 380 kg/mol). Nevertheless, all the SEC plots of the polyisoprenes obtained from **C1**–**C6**/Al^i^Bu_3_/[Ph_3_C][B(C_6_F_5_)_4_] exhibited a monomodal molar mass distribution, which argues in favor of the presence, in each case, of a single-site catalyst.

**Figure 3:**
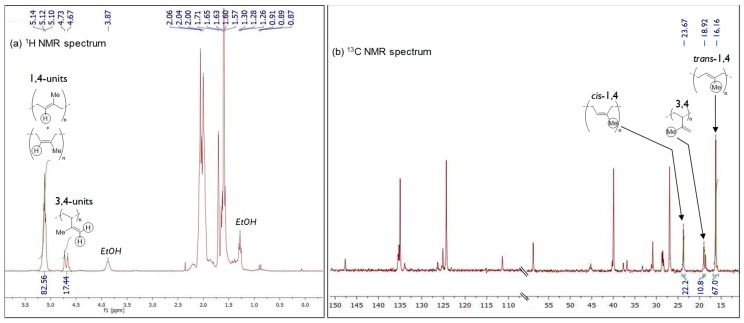
NMR spectra (in CDCl_3_) of the PI obtained from **C1**/Al^i^Bu_3_/[Ph_3_C][B(C_6_F_5_)_4_] (Table 1, entry 1) showing the characteristic resonances of 1,4- and 3,4-units (^1^H-NMR, left) and *trans*/*cis* configuration (^13^C-NMR, right).

**Figure 4:**
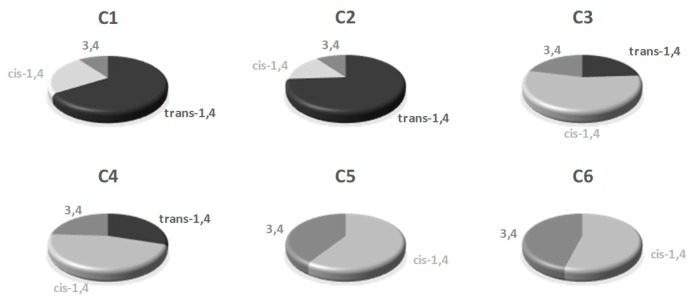
Microstructure content of the polyisoprene obtained from **C1**–**C6**/Al^i^Bu_3_/[Ph_3_C][B(C_6_F_5_)_4_].

All the polyisoprenes were analyzed via NMR spectroscopy studies to determine their microstructure content (see supplementary materials, Figures S17–S22). The corresponding ^1^H and ^13^C-NMR spectra for the polyisoprene obtained in Table 1, entry 1 are displayed in Figure 3. The microstructure content of all the polyisoprenes prepared from Table 1 (entries 1 to 6) are presented in Figure 4.

The polyisoprene acquired from the ternary **C1**/Al^i^Bu_3_/[Ph_3_C][B(C_6_F_5_)_4_] catalytic system consists mainly of 90% of 1,4-units with a majority of *trans*- arrangements (ratio *trans-*1,4/*cis-*1,4 = 66/24) and a small amount (10%) of 3,4 content (Table 1, entry 1; Figure 3 and Figure 4), which reflects the moderate *trans*-1,4 selectivity of catalyst **C1** under these experimental conditions. On the other hand, catalyst issued from **C2** was found to be relatively more selective for *trans*-1,4 polymerization with microstructural content being *trans-*1,4/*cis-*1,4/3,4 = 74/16/10 (Table 1, entry 2). Replacement of the methyl group (**C1**, **C2**) by an hydrogen atom on the carbon of the imino substituent of the ligand (**C3**, **C4**) resulted in a slight decrease of the 1,4-stereoregularity with corresponding increase of 3,4 content (1,4/3,4 = 76–79/21–24, Table 1, entries 3 and 4 with complexes **C3** and **C4**, respectively). In addition, an important increment of the *cis*-1,4 fraction, with respect to the *trans*-1,4 content, was found with these systems, *cis* being predominant now compared to *trans*, unlike what was observed previously with pre-catalysts **C1** and **C2** (**C3**: *trans*/*cis* = 24/55 and **C4**: *trans*/*cis* = 30/46). It is also worth to mention that a decrease in the Al^i^Bu_3_/Fe ratio from 10 to 3 did not affect the stereo- and regio-selectivity of the polymerization, affording polymers with identical yields and microstructural properties (see Supplementary Materials Table S3 and Figures S35–S40).

From these data, it appears that the ketimino-pyridyl-based iron pre-catalysts (**C1** and **C2**), in comparison with the aldimino-pyridyl-based iron complexes counterparts (**C3** and **C4**), preferentially favor the *trans*-1,4-stereoregularity, which is also slightly enhanced, but not significantly, by the presence of sterically hindered *N*-aryl group (**C1** 2,6-*di*-Me_2_Ph vs. **C2** 2,6-*di*-*^i^*Pr_2_Ph: 1,4-*trans* = 67 % vs. 74%, respectively). In addition, this *trans*-1,4-selectivity could be due to an increase in electron density at the iron center, as previously observed with a related electron-rich iron complex bearing an octyl-substituted aldimino-pyridyl ligand [24,26]. Interestingly, the fluorinated pre-catalysts **C5** and **C6** lead to polyisoprenes having a significant fraction of 3,4-motives (41 and 46%, Table 1 entries 5 and 6, respectively) while the 1,4 units exhibit a *cis* configuration only. This is clearly the result of a polymerization process that is governed by kinetic rather than thermodynamic parameters, as already reported with parent iron complexes supported by electron withdrawing fluorinated-aryl-substituted aldimino-pyridyl ligands in combination with an excess of MAO [26].

Substitution of the alkylating agent Al^i^Bu_3_ by AlEt_3_, which has been scarcely used in iron-based polymerization catalysis of conjugated dienes [24], had little effect on the results of the polymerization (see Supplementary Materials Table S1 and Figures S23–S28). One can note however that the polymerizations were less controlled in terms of molar masses for **C1** and **C2** with the presence of a very small amount of polyisoprene displaying high *M*_n_ (Supplementary Materials, Figure S54). In contrast, the ternary **C3** and **C4**/AlEt_3_/[Ph_3_C][B(C_6_F_5_)_4_] catalytic systems resulted in a better control of the polymerization, affording *M*_n_ and *Ð* similar to those obtained with the **C1** and **C2**/Al^i^Bu_3_/[Ph_3_C][B(C_6_F_5_)_4_] systems. With regard to the polymerization of isoprene with complexes **C5** and **C6**, very high *M*_n_ values were obtained as previously found for Al^i^Bu_3_, which again argues for a much higher propagation rate compared to the initiation steps. However, no changes were noticed regarding the selectivity of the polymerization with all complexes by comparison with the results obtained with Al^i^Bu_3_ as alkylating agent (Table 1 vs. Table S1). 

The same polymerization reactions were then studied in the presence of an excess of MAO as co-catalyst (500 equiv. of MAO per Fe) (see supplementary materials Table S2 and Figures S29–S34). The *M*_n_ values of the polyisoprene obtained from **C1**–**C4**/MAO were found to be lower than those produced with Al^i^Bu_3_ or AlEt_3_/[Ph_3_C][B(C_6_F_5_)_4_] as co-reagents, likely due, in some extent, to reversible chain transfer with aluminum. Moreover, a small amount of polyisoprene displaying high *M*_n_ was found for polymerization reactions conducted with complexes **C2**, **C3** and **C4** (see Supplementary Materials, Figure S54). As indicated previously, in the case of the **C5** and **C6** complexes, the same tendency to obtain high molar masses was observed, with the polymer obtained from **C6** exhibiting a bimodal distribution of the molar masses. One can conclude that whatever the nature of the co-catalyst, faster propagation than initiation occurs with the fluorinated pre-catalysts **C5** and **C6**. The microstructures of the obtained polymers were found to be quite comparable to those prepared with **C1**–**C6**/Al^i^Bu_3_ or AlEt_3_/[Ph_3_C][B(C_6_F_5_)_4_] catalytic systems.

The polymerization of isoprene using the **C4**/MAO catalytic systems has already been studied by Chen and coworkers (isoprene/**C4**/MAO = 2500/1/500) [25], which has led to the formation of polyisoprenes in good yield (83% in 2 h at 25 °C) with a slight preference for 1,4-*cis*-stereoregularity (*trans-*1,4/*cis-*1,4/3,4 = 5/70/25). In our case, for isoprene/**C4**/MAO = 500/1/500, the amount of *trans*-1,4 units proved to be higher than that reported previously, whereas the proportion of *cis*-1,4 contents was lower with identical amount of 3,4-motives (*trans-*1,4/*cis-*1,4/3,4 = 30/46/24; Supplementary Materials Table S2, entry 4 and Figure S32). In order to compare these different results, we performed the polymerization of isoprene using the same experimental conditions as those described by Chen (2 h at 25 °C), i.e., dissolution of the pre-catalyst **C4** (8 μmol) in 2 mL of dichloromethane followed by the addition of 7 mL of toluene with isoprene/**C4**/MAO = 2500/1/500. Again, a significant amount of polyisoprene containing *trans*-1,4 units was isolated (*trans-*1,4/*cis-*1,4/3,4 = 21/52/27, see Supplementary Materials, Table S4 and Figure S41). The reason for this difference in result is unknown at this stage, but we can confirm that it does not originate from the use of a chlorinated solvent to dissolve the pre-catalyst.

### 2.3. Kinetic Studies of the Polymerization of Isoprene with the Iron-Based Complexes ***C1**–**C6***

Thereafter, the polymerization processes were optimized by conducting the kinetics of the polymerization with **C1**–**C6**/Al^i^Bu_3_/[Ph_3_C][B(C_6_F_5_)_4_] (1/3/1) catalytic combinations at 23 °C. The studies were carried out with 5000 equiv. isoprene to ensure better reliability, as the too highly active systems prevented us from correctly evaluating the kinetics at 500, 1000 and 2000 equiv. of isoprene/Fe. Aliquots were taken at different times during the course of the polymerization to determine the conversions via ^1^H-NMR (see Supplementary Materials, Table S5). The molecular characteristics of the last sample of polyisoprene for each polymerization run are presented in Table 2 and the Supplementary Materials (Figures S42–S47).

**Figure 5:**
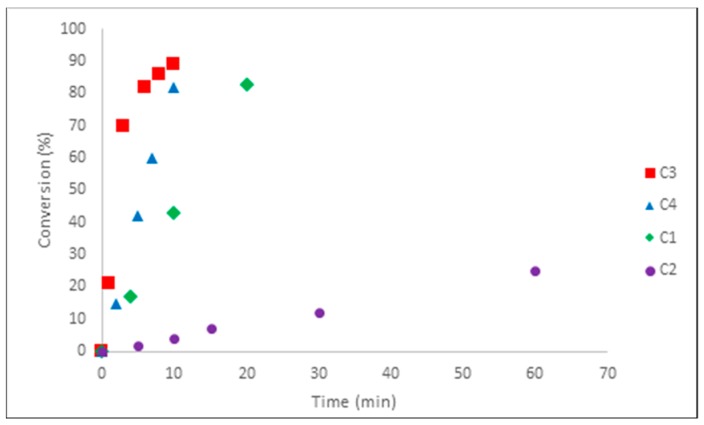
Monomer conversion (%) in function of time (min) for pre-catalysts **C1**–**C4** (Isoprene/Al*^i^*Bu_3_/[Ph_3_C][B(C_6_F_5_)_4_]/Fe = 5,000/3/1/1).

Figure 5 shows the plot of conversion vs. time for complexes **C1**–**C4** as pre-catalysts. The kinetic assessments of complexes **C5** and **C6** could not be obtained because of their extremely high reactivity: full conversion was obtained in less than 1 min (even in 15 s, full conversion was observed), along with notable exothermicity for both pre-catalysts. 

From the profile of the curves, the activity of the complexes in the catalytic combinations is in the order of **C6** ≈ **C5** (TOF > 1,000,000 h^−1^) >> **C3** (TOF = 26,700 h^−1^) > **C4** (TOF = 23,400 h^−1^) > **C1** (TOF = 12,450 h^−1^) > **C2** (TOF = 1,250 h^−1^). For pre-catalysts **C2**–**C4**, 80 % conversion was reached within less than 20 min. Thus, the various pre-catalysts when employed for isoprene polymerization were found to be highly active by comparison with data from the literature [8]. The catalyst based on complex **C1** displayed the lowest activity, although showing the highest *trans*-selectivity amongst this series of complexes. Conversely, the fluorinated complexes **C6** and **C5** have proved to be the most active complexes and, to our knowledge, they display the highest activities reported to date for an iron-catalyzed polymerization of isoprene [8].

It appears that the polymerization is quite controlled for complexes **C1**–**C4**, displaying a nearly first order kinetic profile for all (see Supplementary Materials, Figure S16), which speaks in favor of minimal loss of active species along the polymerization process. These results prompted us to investigate the living character of the polymerization, using pre-catalysts **C1** and **C2**, by sequential addition of isoprene (*vide infra*). 

### 2.4. Sequential Polymerization of Isoprene with ***C1–C2***/Al^i^Bu_3_/[Ph_3_C][B(C_6_F_5_)_4_]

**Figure 6:**
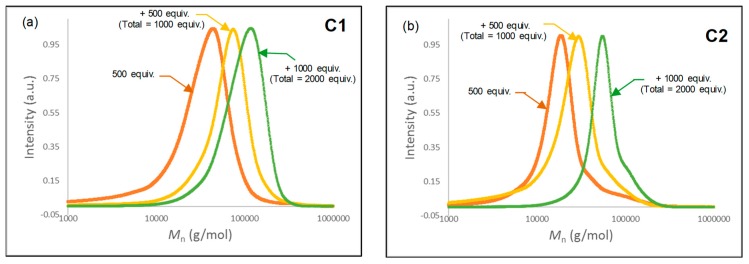
SEC traces of sequential polymerization using pre-catalysts (a, left) **C1** and (b, right) **C2**.

The living polymerization tests were carried out with the ternary **C1** and **C2**/Al^i^Bu_3_/[Ph_3_C][B(C_6_F_5_)_4_] (1/3/1) catalytic systems by sequential addition of monomer in three steps. The polymerization was preliminary conducted with 500 equiv. of isoprene/Fe at room temperature. After completion of this first stage of the polymerization (15 min for **C1** and 30 min for **C2**), an aliquot was withdrawn from the reaction medium to determine the conversion and for SEC analysis, then an additional amount of isoprene (500 equiv./Fe) was added. The last step of the sequential polymerization was achieved by adding 1,000 equiv. of isoprene (after a total time of 30 min for **C1** and 60 min for **C2**) before withdrawing an aliquot; the results are displayed in Table 3, with the SEC traces obtained for each fraction of converted monomer illustrated in Figure 6. 

Analyses of all withdrawn aliquots reveal a continuously increase in isoprene conversion for both pre-catalysts (Table 3, entries 1 and 2). In addition, the SEC traces of the three fractions of the growing polyisoprene chains prepared from the ternary **C1**/Al^i^Bu_3_/[Ph_3_C][B(C_6_F_5_)_4_] and **C2**/Al^i^Bu_3_/[Ph_3_C][B(C_6_F_5_)_4_] catalytic systems show for each a monomodal curve (Figure 6a,b, respectively) with a steady growth of *M*_n_ and a continuous decrease of the dispersity throughout the progression of the polymerization.

For both pre-catalysts, the *M*_n_ resulting from all the aliquots was lower than expected and indicates that the polymerization is not completely controlled, probably due to the presence of chain transfer with aluminum, which was more pronounced for **C2** than for **C1**. Nevertheless, for both runs, the gradual increment of *M*_n_ from the three-stage sequential addition of monomer suggests that the polymerization of isoprene with **C1**/Al^i^Bu_3_/[Ph_3_C][B(C_6_F_5_)_4_] and **C2**/Al^i^Bu_3_/[Ph_3_C][B(C_6_F_5_)_4_] display a *quasi-*living character.

### 2.5. Polymerization of Isoprene with Complexes ***C3**–**C6*** at Lower Temperatures

When conducting isoprene polymerization experiments at room temperature using the complexes **C5** and **C6**, we observed an instantaneous increase in the viscosity of the reaction medium. Consequently, the polymerization of isoprene was assessed at low temperature using **C5** and **C6** in presence of Al^i^Bu_3_/[Ph_3_C][B(C_6_F_5_)_4_] for a sake of better control and improvement of the selectivity. This was also performed with fairly *cis* selective **C3** and **C4** pre-catalysts under the same conditions (Table 4, Figures S48–S53 and Figure S54). 

As a result, all the catalytic systems displayed in Table 4 were found active at temperatures as low as −40 °C (Table 4). In contrast, traces of polymer were isolated from the polymerization carried out at −78 °C for the two complexes **C5** and **C6** (Table 4, entries 4 and 6, respectively), reflecting a probable lack of initiation at this temperature. The ternary **C5** and **C6**/Al^i^Bu_3_/[Ph_3_C][B(C_6_F_5_)_4_] systems displayed high catalytic activity at −40 °C (TOF = 3000 h^−1^) however, the selectivity for 1,4 and 3,4 motives was maintained throughout all the experiments (Table 4, entries 1–3 and 5). This observation reflects an absence of selectivity dependence as a function of temperature, with a similar *cis*/3,4 ratio for the polymerization conducted from room temperature to −40 °C.

**Scheme 2:**
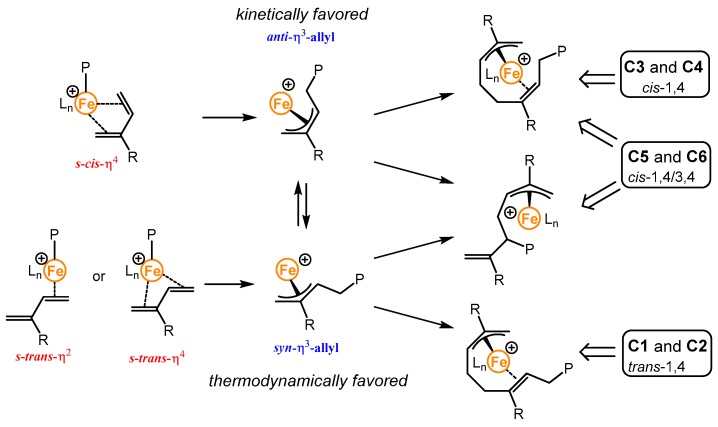
Elementary coordination and insertion steps in isoprene polymerization.

On the other hand, the ternary **C3** and **C4**/Al^i^Bu_3_/[Ph_3_C][B(C_6_F_5_)_4_] catalytic systems exhibit moderate activity at −40 °C (TOF = 100 h^−1^), with the obtained polymers showing narrow dispersity but very high *M*_n_ values compared to the expected *M*_n_, presumably due to a rate of propagation being higher than the initiation step at low temperature. In addition, a slight increase of 1,4-selectivity, at the expense of 3,4 selectivity was observed at −40 °C for **C3** (1,4/3,4 = 89/11) and **C4** (1,4/3,4 = 88/12) complexes when compared to those at 25 °C (Table S3, 1,4/3,4 = 78/22 for **C3** and 1,4/3,4 = 75/25 for **C4**). More interestingly, the polyisoprene obtained from the catalytic systems based on **C3** and **C4** at −40 °C shows a unique *cis* configuration for the 1,4-selectivity (*trans*/*cis* = 0/89 for **C3** and 0/88 for **C4**). Clearly, the propagation takes place via an *anti* (kinetic) active species conformer that is not prone to isomerize into the *syn* (thermodynamic) conformer under these low temperature conditions (Scheme 2, *vide infra*) [40,41].

## 3. Concluding Remarks

**Figure 7:**
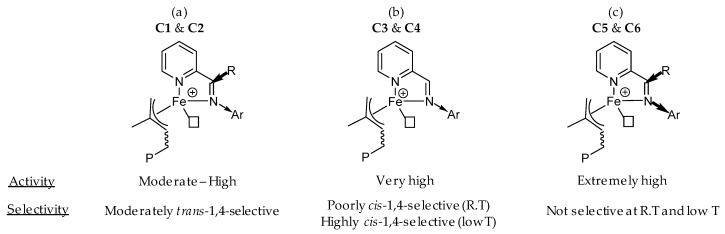
Structure-properties relationships in iminopyridyl iron-based catalysts issued from (**a**) ketiminopyridyl **C1**–**C2**, (**b**) aldiminopyridyl **C3**–**C4** and (**c**) fluoroaryl-ketiminopyridyl **C5**–**C6**.

In this study, we observed that the activity of the catalytic system based on iminopyridyl-iron complexes is clearly related to the electron density on iron and, to a lesser extent, to the environment of the coordination sphere of the metal center. Indeed, an increase of the electrophilicity on the metal center leads to stronger monomer coordination and presumably faster chain propagation, while an increase in steric hindrance, conferred by the presence of bulky *N*-aryl substituent, results in a decrease of activity that could be attributed to the difficulty of the incoming monomer to coordinate with the active species. As such, the ketiminopyridyl complexes **C1**/**C2**, which comprise a more electron-rich metal center, were found to be less active (Figure 7a; TOF = 12,450 and 1250 h^−1^, respectively) than their homolog **C3**/**C4** aldiminopyridyl complexes (Figure 7b; TOF = 26,700 and 23,400 h^−1^, respectively). This is attributable to the more inductive donor effect of the methyl group on the carbon of the imino substituent of the ligands **L1** and **L2**, when compared to **L3** and **L4**. Moreover, an increase of the steric hindrance around the iron metal center, for the same set of complexes with *N*-aryl group = 2,6-*^i^*Pr_2_ for **C2** and **C4** vs. 2,6-Me_2_ for **C1** and **C3**, resulted in a decrease of activity (in the order **C2** > **C1** and **C4** > **C3**), which could be connected to a restricted coordination of the isoprene to the metal center. In contrast, in the case of the ketiminopyridyl complexes **C1**/**C2** and **C5**/**C6**, the lower reactivity in **C1**/**C2** is largely compensated for the remarkable electron-withdrawing effect of the fluorinated *N*-aryl substituent in **C5**/**C6** (Figure 7c), which leads to extremely active catalysts (TOF > 1,000,000 h^−1^), the most effective among the families of iron-catalyzed polymerization of isoprene described to date [8].

Regarding the selectivity, the concomitant presence of methyl group on the carbon of the imino substituent and *N*-aryl group with alkyl substituent on the *ortho* position in **C1**/**C2** favor *trans*-1,4 selectivity (Figure 7a) up to 74% under our conditions. Although, the electronic effect of fluorinated *N*-aryl substituent in **C5**/**C6** leads to *cis*-1,4 selectivity, presumably due to a preferential η^4^-*cis* coordination of the incoming monomer, but combined with the presence of methyl group on the carbon of the imino substituent, this results in a substantial amount of 3,4 arrangement (Figure 7c). These results are in line with previous experiments done by the group of Wang with fluorinated *N*-aryl substituted aldiminopyridine iron complexes activated with MAO [26]. Interestingly, the iron catalyst systems based on **C5**/**C6** are not temperature dependent in term of regio- and stereo-selectivity. Regarding the high *cis*-1,4 rate (89%) observed with **C3**/**C4** at low temperature, it reveals the importance of the *anti*/*syn* isomerization (Scheme 2) of the propagating species in the stereoselective iron-catalyzed 1,3-dienes polymerization (Figure 7b).

## 4. Experimental

### 4.1. General Information

An inert atmosphere by using Schlenk techniques or in a dry/inert glovebox (O_2_ < 1 ppm, H_2_O < 1 ppm, Jacomex, Dagneux, France) were used to implement all manipulations. Toluene was passed through an alumina column from an Mbraun SPS (MBraun France, Mérignac, France), distilled over sodium/benzophenone and stored on 4 Å molecular sieves in a glove box before used. Isoprene (Sigma-Aldrich, St Quentin Fallavie, France) was dried over calcium hydride, distilled once over 4 Å molecular sieves, and stored at −20 °C in the glove box before used. All the organic reagents (2-acetylpyridine, pyridine-2-carboxaldehyde and aniline derivatives) were acquired from Sigma-Aldrich or Fisher Scientic S.A.S. (Illkrich, France), and used as received. Triisobutylaluminum [Al(*^i^*Bu)_3_, Sigma-Aldrich], triethylaluminum [Al(Et)_3_, Sigma-Aldrich], methylaluminoxane (MAO, 10 wt% in toluene, Sigma-Aldrich) and trityl tetrakis(pentaflurophenyl)borate {[Ph_3_C][B(C_6_F_5_)_4_]} (TCI Europe N.V., Zwijndrecht, Belgium) were stored in the glove box and used as received. ^1^H-, ^13^C- and ^19^F-NMR spectra were recorded on an Avance 300 instrument (Bruker France, Palaiseau France) at 300 K. All ^1^H chemical shifts (reported in [ppm]) were referenced by using residual signals of the deuterated solvents. Elemental analyses were performed by Céline Delabre on an Micro Cube apparatus Elementar Vario (Elementar France SARL, Lyon, France) at UCCS, University Lille Nord de France. In the case of polymers analyses, the conversion as well as the composition of the polymers and the microstructural contents were calculated by mean of ^1^H- and ^13^C-NMR spectroscopy analysis using Topspin or MestreNova softwares. Size exclusion chromatography (SEC) analysis of the samples were performed in THF as an eluent at 40 °C (1 mL/min) with a SIS HPLC pump (Waters S.A.S, Saint-Quentin-en-Yvelines, France), a Waters 410 refractometer, and Waters Styragel columns (HR2, HR3, HR4, and HR5E) calibrated with polystyrene standards. FTIR analyses were performed on an ATR spectrometer (Shimadzu France, Marne-la-Vallée, France). 

### 4.2. General Procedure for the Synthesis of Ligands ***L1**–**L4***

A solution of corresponding substituted aniline (8 mmol) and pyridine-2-carboxaldehyde/2-acetyl pyridine (10 mmol) in methanol (15 mL) was prepared in a round-bottomed flask equipped with a magnetic stirrer. Catalytic amount of formic acid (3–4 drops) was added subsequently before the mixture was refluxed overnight at 90 °C. 

*N-(2,6-Dimethylphenyl)-1-(pyridin-2-yl)ethan-1-imine* (**L1**): the reaction mixture was concentrated under reduced pressure which was further dissolved in pentane, dried over sodium sulfate and filtered. The mixture was concentrated again under reduced pressure to obtain a crude product which was further recrystallized in methanol at –20 °C to obtain light-yellow crystals. Yield: 46%. ^1^H-NMR (300 MHz, CDCl_3_, 25 °C) δ (ppm) = 8.71 (d broad, 1H, *H_a_*), 8.40 (dd broad, 1H, *H*_b_), 7.82 (dd broad, 1H, *H_c_*), 7.39 (dd broad, 1H, *H*_d_), 7.09–6.98 (m, 3H, *H*_e,f,_), 2.20 (s, 3H, *H_g_*), 2.05 (s, 6H, *H*_h_). IR/cm^−1^ = 1637 ν(C=N). The data are similar to those found in the literature [33,34] 

*N-(2,6-Diisopropylphenyl)-1-(pyridin-2-yl)ethan-1-imine* (**L2**): similar to **L1**. The crude product obtained was purified over silica column with ethyl acetate/petroleum ether (1/10) as an eluent to yield a yellow oil. Yield: 15%. ^1^H-NMR (300 MHz, CDCl_3_, 25 °C) δ (ppm) = 8.70 (ddd, ^3^*J*_HH_ = 4.8 Hz, ^4^*J*_HH_ = 1.7 Hz, ^5^*J*_HH_ = 0.8 Hz, 1H, *H*_a_), 8.41 (d, ^3^*J*_HH_ = 8.1 Hz, 1H, *H*_b_), 7.85 (ddd, ^3^*J*_HH_ = 8.1, 8.1 Hz, ^4^*J*_HH_ = 1.7 Hz, 1H, *H*_c_), 7.42 (ddd, ^3^*J*_HH_ = 8.1 Hz, ^4^*J*_HH_ = 4.8 Hz, ^5^*J*_HH_ = 0.8 Hz, 1H, *H*_d_), 7.21–7.03 (m, 3H, *H*_e,f_), 2.74 (sept, ^3^*J*_HH_ = 6.9 Hz, 2H, *H*_g_), 2.24 (s, 3H, *H*_h_), 1.15 (d, ^3^*J*_HH_ = 6.9 Hz, 12H, *H*_i_). The data are similar to those found in the literature [32,33,34].

*N-(2,6-Dimethylphenyl)-1-(pyridin-2-yl)methanimine* (**L3**): similar to **L1**. The crude product obtained is a yellow-viscous oil, which crystallizes slowly in methanol to yield a yellow solid. Yield: 89%.^1^H-NMR (300 MHz, CDCl_3_, 25 °C) δ (ppm) = 8.76 (d, ^3^*J*_HH_ = 4.7 Hz, 1H, *H*_a_), 8.42 (s, 1H, *H_b_*), 8.34 (d, ^3^*J*_HH_ = 7.9 Hz, 1H, *H*_c_), 7.85 (dd, ^3^*J*_HH_ = 7.9, 7.9 Hz, 1H, *H*_d_), 7.46–7.37 (m, 1H, *H*_e_), 7.14–6.96 (m, 3H, *H*_f,g_), 2.23 (s, 6H, *H*_h_). IR/cm^−1^ = 1637 ν(C=N). The data are similar to those found in the literature [33]. 

*N-(2,6-Diisopropylphenyl)-1-(pyridin-2-yl)methanimine* (**L4**): similar to **L3**. The crude product was recrystallized in ethanol to afford the product as yellow crystals. Yield: 29 %. ^1^H-NMR (300 MHz, C_6_D_6_, 25 °C) δ (ppm) = 8.59 (s, 1H, *H*_a_), 8.46 (dd, ^3^*J*_HH_ = 4.8 Hz, ^4^*J*_HH_ = 1.7 Hz, 1H, *H*_b_), 8.27 (d, ^3^*J*_HH_ = 7.9 Hz, 1H, *H*_c_), 7.13–7.04 (m, 3H, *H*_e,f,g_), 6.65 (dd, ^3^*J*_HH_ =7.5, 4.8 Hz, 1H, *H*_d_), 3.15 (sept, ^3^*J*_HH_ = 6.8 Hz, 2H, *H*_h_), 1.15 (d, ^3^*J*_HH_ = 6.8 Hz, 12H, *H*_i_). IR/cm^−1^ = 1633 ν(C=N). The data are similar to those found in the literature [33,35].

*N-(3,5-bis(Trifluoromethyl)phenyl)-1-(pyridin-2-yl)ethan-1-imine* (**L5**): a mixture of 2-acetyl pyridine (0.69 mL, 6.2 mmol) and 3,5-bis(trifluoromethyl)aniline (0.97 mL, 6.2 mmol) in toluene (25 mL) was prepared in a round-bottomed flask equipped with a magnetic stirrer and Dean-Stark apparatus. Catalytic amount of *p*-toluenesulfonic acid (2–3 mg) was added to the reaction mixture and the solution was refluxed overnight. The obtained mixture was concentrated under reduced pressure to obtain a yellow crude oil. Yield: 43%. ^1^H-NMR of **L5** (300 MHz, CD_2_Cl_2_, 25 °C) δ (ppm) 8.67 (dd, ^3^*J*_HH_ = 4.8 Hz, ^4^*J*_HH_ = 1.7 Hz, 1H, *H*_a_), 8.25 (d, ^3^*J*_HH_ = 7.8 Hz, 1H, *H_c_*), 7.83 (dd, *J =* 7.8, 7.8 Hz, 1H, *H_d_*), 7.65 (s, 1H, *H_b_*), 7.42 (dd, ^3^*J*_HH_ = 7.8, 4.8 Hz, 1H, *H_e_*), 7.30 (s, 2H, *H_f_*), 2.37 (s, 3H, *H_g_*). ^19^F-NMR (282 MHz, C_6_D_6_, 25 °C, δ): -63.32 (s, 6F). IR/cm^−1^ = 1649 ν(C=N). The data are similar to those found in the literature [36].

*N-(Perfluorophenyl)-1-(pyridin-2-yl)ethan-1-imine* (**L6**): a mixture of 2-acetyl pyridine (0.93 mL, 8.3 mmol) and 2,3,4,5,6-pentafluoroaniline (1.49 g, 8.1 mmol) with catalytic amounts of *p*-toluenesulfonic acid and sodium sulfate (10 mg) in toluene (14 mL) was refluxed overnight in an Ace pressure tube at 110 °C. The mixture obtained was filtered over cotton, extracted with dichloromethane and concentrated under reduced pressure to obtain a brown solid. The product was extracted with pentane, filtered and concentrated under vacuum. The crude compound was further recrystallized in pentane to obtain orange-brown crystals. Yield: 10%. ^1^H-NMR (300 MHz, C_6_D_6_) δ (ppm) = ^1^H-NMR (300 MHz, C_6_D_6_) δ (ppm) = 8.35 (dd, ^3^*J*_HH_ = 4.8 Hz, ^4^*J*_HH_ = 1.7 Hz, 1H, *H*_a_), 8.25 (d, ^3^*J*_HH_ = 7.8 Hz, 1H, *H*_b_), 7.03 (ddd, ^3^*J*_HH_ = 7.8 Hz, 7.8 Hz, ^4^*J*_HH_ = 1.7 Hz, 1H, *H*_c_), 6.63 (dd, ^3^*J*_HH_ = 7.8 Hz, ^3^*J*_HH_ = 4.8 Hz, 1H, *H*_d_), 2.20 (s, 3H, *H*_e_). ^19^F-NMR (282 MHz, C_6_D_6_, 25 °C) δ (ppm) = −152.5 (d, *J =* 23.8 Hz, 2F, F_meta_), −163.1 (t, *J =* 21.7 Hz, 1F, F_para_), −163.6 (dd, *J =* 23.8, 21.7 Hz, 2F, F_ortho_). ^13^C-NMR (75 MHz, C_6_D_6_, 25 °C) δ (ppm) = 175.1, 154.9, 148.5, 139.7, 139.3, 139.1, 138.7, 135.9, 125.4, 121.8, 17.5. At this stage, we could not unambiguously assign the ^13^C NMR spectrum. IR/cm^−1^ = 1647 ν(C=N). Anal. Calcd. for C_13_H_7_N_2_F_5_: C 54.56, H 2.47, N 9.79; found C 54.57, H 2.48, N 9.72.

### 4.3. General Procedure for the Synthesis of Complexes ***C1**–**C6***

The corresponding ligand (3.5 mmol), anhydrous FeCl_2_ (3.5 mmol) and dry THF (32 mL) were added to a Schlenk inside the glove box. The mixture was stirred overnight under argon atmosphere. The excess solvent was evaporated under reduced pressure and the product was washed with dry pentane (3 × 30 mL), further dried under high vacuum to obtain a powder. 

*[N-(2,6-Dimethylphenyl)-1-(pyridin-2-yl)ethan-1-imine]FeCl*_2_ (**C1**): purple solid. Yield: 82%. ^1^H-NMR (300 MHz, CD_2_Cl_2_, 25 °C, δ): 99.0 (Δν_1/2_ = 134 Hz, 1H), 75.1 (Δν_1/2_ = 55 Hz, 1H), 51.0 (Δν_1/2_ = 48 Hz, 1H), 3.1 (Δν_1/2_ = 34 Hz, 3H), 2.4 (Δν_1/2_ = 28 Hz, 12H), 0.4 (Δν_1/2_ = 39 Hz, 1H), −5.6 (Δν_1/2_ = 94 Hz, 2H), −17.8 (Δν_1/2_ = 29 Hz, 2H), −19.3 (Δν_1/2_ = 42 Hz, 1H). IR/cm^−1^ = 1591 ν(C=N). The data are similar to those found in the literature [34].

*[N-(2,6-Diisopropylphenyl)-1-(pyridin-2-yl)ethan-1-imine*]*FeCl*_2_ (**C2**): dark purple powder. Yield: 57%. ^1^H-NMR (300 MHz, CD_2_Cl_2_, 25 °C, δ): 102.7 (Δν_1/2_ = 80 Hz, 3H), 72.6 (Δν_1/2_ = 54 Hz, 1H), 65.5 (Δν_1/2_ = 1089 Hz, 1H), 49.1 (Δν_1/2_ = 61 Hz, 1H), 10.6 (Δν_1/2_ = 194 Hz, 6H), 3.0 (Δν_1/2_ = 28 Hz, 2H), −18.4 & −18.5 (2H). IR/cm^−1^ = 1589 ν(C=N). The data are similar to those found in the literature [34,37].

*[N-(2,6-Dimethylphenyl)-1-(pyridin-2-yl)methanimine]FeCl_2_* (**C3**): violet powder. Yield: 61%. ^1^H-NMR (300 MHz, CD_2_Cl_2_, 25 °C, δ): 85.9 (Δν_1/2_ = 183 Hz, 1H), 64.4 (Δν_1/2_ = 1526 Hz 1H), 54.2 (Δν_1/2_ = 62 Hz, 1H), 50.5 (Δν_1/2_ = 77 Hz, 1H), 11.3 (Δν_1/2_ = 228 Hz, 6H), 5.1 (1H), −13.5 (Δν_1/2_ = 31 Hz, 2H), −17.3 (Δν_1/2_ = 47 Hz, 1H). IR/cm^−1^ = 1593 ν(C=N). The data are similar to those found in the literature [34]. 

*[N-(2,6-Diisopropylphenyl)-1-(pyridin-2-yl)methanimine]FeCl_2_* (**C4**): violet powder. Yield: 75%. ^1^H-NMR (300 MHz, CD_2_Cl_2_, 25 °C, δ): 85.6 (Δν_1/2_ = 156 Hz, 1H), 62.5 (Δν_1/2_ = 2300 Hz, 1H), 54.8 (Δν_1/2_ = 61 Hz, 1H), 51.1 (Δν_1/2_ = 75 Hz, 1H), 3.8 (Δν_1/2_ = 50 Hz, 12H), 2.2 (Δν_1/2_ = 447 Hz, 1H), −3.1 (Δν_1/2_ = 95 Hz, 2H), −13.0 (Δν_1/2_ = 34 Hz, 2H), -18.0 (Δν_1/2_ = 48 Hz, 1H). IR/cm^−1^ = 1593 ν(C=N). The data are similar to those found in the literature [38,39]. 

*[N-(3,5-bis(trifluoromethyl)phenyl)-1-(pyridin-2-yl)ethan-1-imine]FeCl_2_* (**C5**): azure blue powder. Yield: 79%. ^1^H-NMR (300 MHz, CD2Cl2, 25 °C, δ): 72.7 (Δν_1/2_ = 496 Hz, 1H), 67.1 (Δν_1/2_ = 611 Hz, 1H), 53.2 & 50.65 (1H), 12.4 (Δν_1/2_ = 1146 Hz, 1H), −7.5 (Δν_1/2_ = 215 Hz, 3H), -11.6 (Δν_1/2_ = 162 Hz, 1H), −16.6 (Δν_1/2_ = 1711 Hz, 1H). IR/cm^−1^ = 1597 ν(C=N). Anal. Calcd. for C_15_H_10_N_2_F_6_FeCl_2_: C 39.25, H 2.20, N 6.10; found C 40.10, H 2.43, N 6.07.

*[N-(Perfluorophenyl)-1-(pyridin-2-yl)ethan-1-imine]FeCl_2_* (**C6**): navy blue powder. Yield: 83%. ^1^H-NMR (300 MHz, CD_2_Cl_2_, 25 °C, δ): 70.1 (Δν_1/2_ = 809 Hz, 2H), 53.6 (Δν_1/2_ = 555 Hz, 1H), 48.8 (Δν_1/2_ = 304 Hz, 1H), -19.4 (Δν_1/2_ = 487 Hz, 3H). IR/cm^−1^ = 1595 ν(C=N). Anal. Calcd. for C_13_H_7_N_2_F_5_FeCl_2_ + one molecule of THF (C_4_H_8_O): C 42.09, H 3.06, N 5.72; found C 42.10, H 3.12, N 5.78.

### 4.4. General Procedure for Isoprene Polymerization 

In a typical polymerization experiment, a Schlenk reactor (20 mL) was heated, dried in a vacuum and purged with argon three times before introducing it to the glove box. Iron (II) chloride complex (10 µmol, 1 eq.) and toluene (3 mL) were added to the reactor followed by the addition of an aluminum co-catalyst at 23 °C. 

*Activation with AlR_3_ (100 µmol, 10 eq.)/[Ph_3_C][B(C_6_F_5_)_4_] (10 µmol, 1 eq.):* the reaction mixture was stirred for 2-3 min and trityltetrakis(pentafluorophenyl)borate was added as a solution in 2 mL of toluene at 23 °C. The mixture was stirred for 2 min and isoprene (0.346 g, 0.5 mL, 500 eq.) was added. The reaction mixture was stirred for the desired time before being quenched with 2–3 drops of HCl followed by dilution in toluene. The polymer was precipitated in ethanol (150 mL) containing the stabilizing agent BHT (*tert*-butylhydroxytoluene), isolated and dried in vacuum for at least 4 h to yield a gummy solid.

*Activation with MAO (5 mmol, 500 eq.):* the mixture was stirred for 2–3 min and isoprene (0.346 g, 0.5 mL, 500 eq.) was added. The reaction mixture was stirred for the desired time before being quenched with 2–3 drops of HCl followed by dilution in toluene. The polymer was precipitated in ethanol (150 mL) containing the stabilizing agent BHT, isolated and dried in vacuum for at least 4 h to yield a gummy solid.

For a given aliquot, the conversion was determined using the chemical shifts of the olefinic protons of polyisoprene (5.11 and 4–4.6 ppm) and isoprene (6.5–6.42 ppm). The aliquots that were taken at several intervals were quenched with isopropanol present inside the NMR tubes containing the benzene-d6 capillaries. The percentage conversion was calculated according to the equation:

$$\left[\%\;Conversion\right]=\frac{Area\;normalised\;for\;polymer}{Area\;normalised\;for\;polymer+Area\;normalised\;for\;monomer\;*}$$

$$=\frac{I\left(5.14-5.10\;ppm\right)+\;\frac{I\left(4.72-4.67\;ppm\right)}{2}}{I\left(5.14-5.10\;ppm\right)+\frac{I\left(4.72-4.67\;ppm\right)}{2}+\left(I\left(6.5-6.42\;ppm\right)\;or\;I\left(5.22-5\;ppm\right)\right)}$$

* Can be chosen between the two olefinic regions of isoprene depending on the better resolution of peaks (see Supporting Materials).

### 4.5. Calculation of Microstructure Contents 

As previously known [42], the characteristic signals of polyisoprene in a ^1^H-NMR spectrum were found at 5.14–5.10 ppm and 4.72–4.67 ppm corresponding to 1,4 and 3,4 units respectively. The percentage content of 1,4 and 3,4 units was determined according to the equations:

$$\left[\%1,4\;content\right]=\frac{I\left(5.14-5.10\;ppm\right)}{I\left(5.14-5.10\;ppm\right)+\frac{I\left(4.72-4.67\;ppm\right)}{2}}$$

$$\left[\%3,4\;content\right]=\frac{\frac{I\left(4.72-4.67\;ppm\right)}{2}}{I\left(5.14-5.10\;ppm\right)+\frac{I\left(4.72-4.67\;ppm\right)}{2}}$$

The characteristic signals at 16.2 and 23.8 ppm in the ^13^C-NMR spectra correspond to the methyl carbon of *cis*-1,4 and *trans*-1,4 polyisoprene motifs, respectively [43]. Therefore, the percentage *cis*-1,4 and *trans*-1,4 content is given by the equations:

$$\left[\%trans-1,4\;content\right]=\frac{I\left(16.2\;ppm\right)}{I\left(16.2\;ppm\right)+I\;\left(23.8\;ppm\right)}$$

$$\left[\%cis-1,4\;content\right]=\frac{I\left(23.8\;ppm\right)}{I\left(16.2\;ppm\right)+I\;\left(23.8\;ppm\right)}$$

## Figures and Tables

**Table 1:** Polymerization of isoprene using **C1**–**C6**/Al^i^Bu_3_/[Ph_3_C][B(C_6_F_5_)_4_] catalytic systems **^a^**.

Entry ^a^	Complex	Conv. (%)	*M*_n(exp)_^b^ (g/mol)	*Đ* ^b^	Microstructure ^c^ (%)	
					1,4 (*trans*/*cis*)	3,4
1	**C1**	>99	44,000	1.5	90 (67/22)	11
2	**C2**	>99	44,000	1.7	90 (74/16)	10
3	**C3**	>99	101,000	2.7	79 (24/55)	21
4	**C4**	>99	84,000	1.5	76 (30/46)	24
5	**C5**	>99	513,000	2.4	59 (0/59)	41
6	**C6**	>99	382,000	1.8	54 (0/54)	46

^a^ Polymerization conditions: 10 µmol of Fe(II) complex; isoprene/Fe/Al^i^Bu_3_/[Ph_3_C][B(C_6_F_5_)_4_] = 500/1/10/1; toluene = 5 mL; time = 1 h; temperature = 25 °C; ^b^ determined by size exclusion chromatography (SEC); ^c^ determined by ^1^H-NMR and ^13^C-NMR; *M*_n(th)_ = 33,700 g/mol (considering one growing chain per metal center); Activity = 34 kg_(PI)∙_mol_(cat)_^−1^ ∙h^−1^ or TOF = 500 h^−1^ for all.

**Table 2:** Polymerization of 5000 equiv. of isoprene/Fe using **C1**–**C6**/Al^i^Bu_3_ /[Ph_3_C][B(C_6_F_5_)_4_] catalytic systems ^a.^

Entry	Complex	Conv. (%)	Time (min)	TOF (h^−1^)	*M*_n(exp)_^b^ (g/mol)	*Đ* ^b^	Microstructure ^c^ (%)	
							1,4 (*trans*/*cis*)	3,4
1	**C1**	83	20	12,450	142,000	1.9	90 (60/30)	10
2	**C2**	25	60	1250	87,000	1.4	84 (69/18)	13
3	**C3**	89	10	26,700	105,000	1.8	79 (25/54)	21
4	**C4**	82	10	23,400	163,000	1.6	74 (26/48)	26
5	**C5**	>99	< 1	> 300,000	323,000	1.7	58 (0/58)	42
6	**C6**	>99	< 1	> 300,000	213,000	1.6	54 (0/54)	46

^a^ Polymerization conditions: 5 µmol of Fe(II) complex; Isoprene/Al^i^Bu_3_/[Ph_3_C][B(C_6_F_5_)_4_]/Fe = 5,000/3/1/1; toluene = 25 mL; temperature = 25 °C; ^b^ determined by SEC; ^c^ determined by ^1^H-NMR and ^13^C-NMR; considering one growing chain per metal center *M*_n(th)_ ≈ 282,700 g/mol for **C1** and **C4**, *M*_n(th)_ = 85,150 g/mol for **C2**, *M*_n(th)_ = 303,100 g/mol for **C3**, *M*_n(th)_ = 337,200 g/mol for **C5** and **C6**.

**Table 3:** Sequential polymerization of isoprene using **C1, C2**/Al^i^Bu_3_ /[Ph_3_C][B(C_6_F_5_)_4_] catalytic systems ^a^.

Entry	Complex	Total Monomer (intermediate addition of monomer) (equiv./Fe)	Time (min)	Conv. (%)	*M*_n(exp)_^b^ (g/mol)	*Đ* ^b^
1	**C1**	500 (500)	15	>99	36,500	1.6
		1000 (+500)	30 (+15)	>99	69,000	1.5
		2000 (+1000)	60 (+30)	>99	102,000	1.3
2	**C2**	500 (500)	30	>99	23,000	2.4
		1000 (+500)	60 (+30)	>99	32,000	2.0
		2000 (+1000)	120 (+60)	>99	62,000	1.3

^a^ Polymerization conditions: 10 µmol of Fe(II) complex; Fe/Al^i^Bu_3_/[Ph_3_C][B(C_6_F_5_)_4_] = 1/3/1; [C]i_soprene_ = 1 mol/L in toluene; temperature = 25 °C; ^b^ determined by SEC; ^c^ determined by ^1^H-NMR and ^13^C-NMR; *M*_n(th)_ = 135,000 g/mol for the last sample (considering one growing chain per metal center).

**Table 4:** Polymerization of isoprene at lower temperatures with pre-catalysts **C3**–**C6** ^a^.

Entry	Complex	T (°C)	Time (min)	Conv. (%)	TOF (h^−1^)	*M*_n(exp)_^b^ (g/mol)	*Đ* ^b^	Microstructure ^c^ (%)	
								1,4 (*trans*/*cis*)	3,4
1	**C5**	0	10	>99	3000	294,000	1.2	58 (0/58)	42
2		–20	10	>99	3000	251,000	1.3	58 (0/58)	42
3		–40	10	>99	3000	173,000	1.3	58 (0/58)	42
4		–78	480	traces	-	-	-	-	-
5	**C6**	–40	10	>99	3000	274,500	1.2	54 (0/54)	46
6		–78	180	traces	-	-	-	-	-
7	**C3**	–40	300	>99	100	169,000	1.3	89 (0/89)	11
8	**C4**	–40	300	>99	100	216,000	1.6	88 (0/88)	12

^a^ Polymerization conditions: 10 µmol of Fe(II) complex; Isoprene/Al^i^Bu_3_/[Ph_3_C][B(C_6_F_5_)_4_]/Fe = 500/1; toluene = 5 mL; reaction times have not been optimized; ^b^ determined by SEC; ^c^ determined by ^1^H-NMR and ^13^C-NMR.

## Supplementary Materials

The following are available online. Figures S1–S3: ^1^H-NMR, ^13^C-NMR and ^19^F-NMR of the ligand **L6**; Figures S4–S8: ^1^H-NMR of the ligands L1-L5; Figures S9–S14: ^1^H-NMR of the complexes **C1**–**C6**; Figure S15**:** ^1^H-NMR spectra of stacked of all iron-based complexes; Table S1: Polymerization of isoprene using **C1**–**C6**/AlEt_3_/[Ph_3_C][B(C_6_F_5_)_4_] (1/10/1) catalytic systems; Table S2: Polymerization of isoprene using **C1**–**C6**/MAO (1/500) catalytic systems; Table S3: Polymerization of isoprene using **C1**–**C6**/Al*^i^*Bu_3_/[Ph_3_C][B(C_6_F_5_)_4_] (1/3/1) catalytic systems; Table S4: Polymerization of isoprene (2500 eq.) using **C4**/MAO (1/500) catalytic systems; Figure S16: Kinetic Profile of Polymerization with complexes **C1**–**C4**; Table S5: Polymerization of 5,000 equiv. of isoprene/Fe using **C1**–**C4**/Al^i^Bu_3_ /[Ph_3_C][B(C_6_F_5_)_4_] catalytic systems; Figures S17–S53: ^1^H- and ^13^C-NMR spectra of the polymers obtained; Figure S54: SEC traces of the polymers obtained.
