# Supplementary material for: Highly Active Iminopyridyl Iron-Based Catalysts for the Polymerization of Isoprene
Source: Molecules. 2019 Aug 21;24(17):3024. doi: 10.3390/molecules24173024 (PMC6749290; doi:10.3390/molecules24173024)
Supplement: Supplementary file 1 [file molecules-24-03024-s001.pdf]

Highly active iminopyridine iron-based catalysts for the polymerization of isoprene

Obaid H. Hashmi, Yohan Champouret * and Marc Visseaux *

UMR 8181–UCCS–Unité de Catalyse et de Chimie du Solide, ENSCL, Centrale Lille, University of Artois, University of Lille, CNRS, F-59000 Lille, France

* Correspondence: yohan.champouret@univ-lille.fr (Y.C.); marc.visseaux@univ-lille.fr (M.V.)

-SUPPORTING INFORMATION-

Figures S1-S3. ^{1}H NMR, ^{13}C NMR and ^{19}F NMR of the ligand **L6**

Figures S4-S8. ^{1}H NMR of the ligands **L1**-**L5**

Figures S9-S14. ^{1}H NMR of the complexes **C1**-**C6**

Figure S15. ^{1}H NMR spectra of stacked of all iron-based complexes

Table S1. Polymerization of isoprene using **C1 – C6**/$AlEt_3$/$[Ph_3C][B(C_6F_5)_4]$ (1/10/1) catalytic systems

Table S2. Polymerization of isoprene using **C1 – C6**/MAO (1/500) catalytic systems

Table S3. Polymerization of isoprene using **C1 – C6**/$Al^{i}Bu_3$/$[Ph_3C][B(C_6F_5)_4]$ (1/3/1) catalytic systems

Table S4. Polymerization of isoprene (2,500 eq.) using **C4**/ MAO (1/500) catalytic systems

Figure S16. Kinetic Profile of Polymerization with complexes **C1**-**C4**

Table S5. Polymerization of 5,000 equiv. of isoprene/Fe using **C1 – C4**/$Al^{i}Bu_3$ /$[Ph_3C][B(C_6F_5)_4]$ catalytic systems [a]

Figures S17-S53. ^{1}H and ^{13}C NMR spectra of the polymers obtained

Figure S54. SEC traces of the polymers obtained

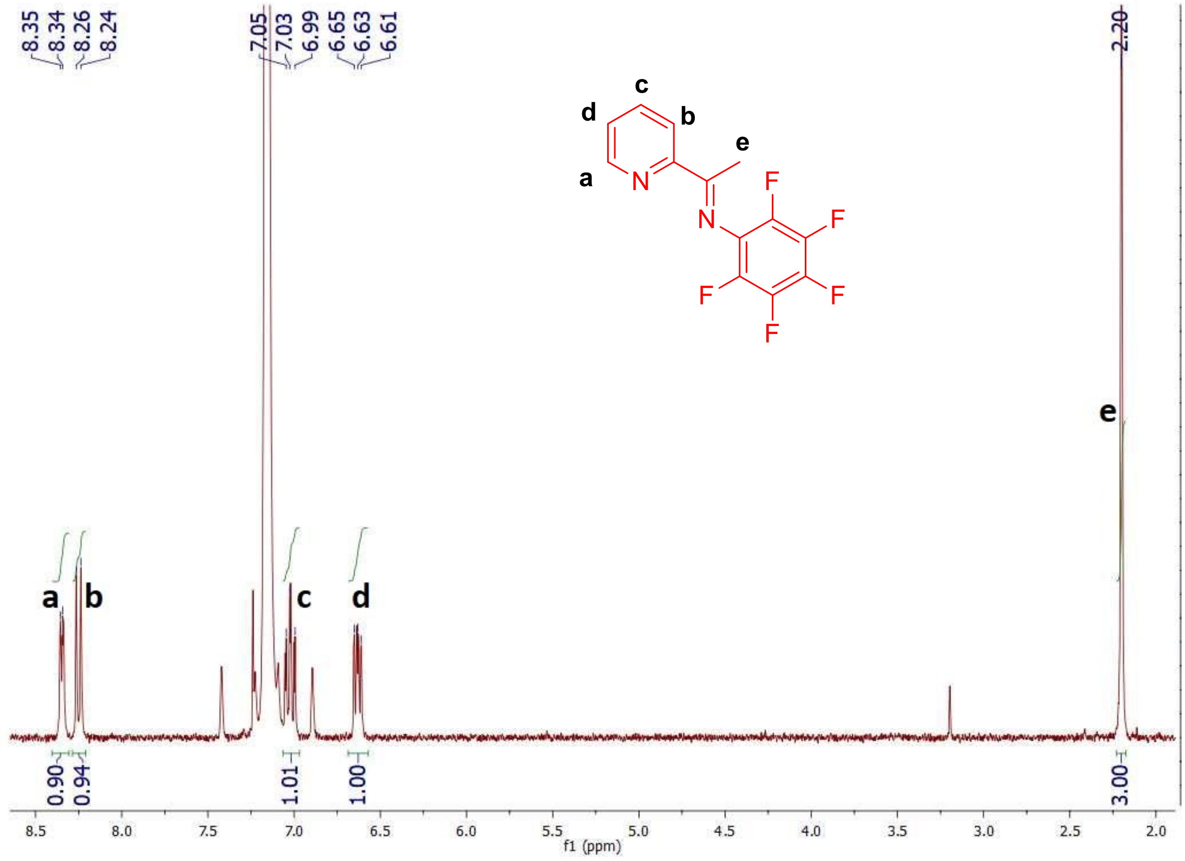

Figure S1. ^{1}H NMR of **L6** (300 MHz, C_6D_6, 25 °C)

^{1}H NMR (300 MHz, C_6D_6) δ (ppm) = 8.35 (dd, $^{3}J_{HH}$ = 4.8 Hz, $^{4}J_{HH}$ = 1.7 Hz, 1H, H_a), 8.25 (d, $^{3}J_{HH}$ = 7.8 Hz, 1H, H_b), 7.03 (ddd, $^{3}J_{HH}$ = 7.8, 7.8 Hz, $^{4}J_{HH}$ = 1.7 Hz, 1H, H_c), 6.63 (dd, $^{3}J_{HH}$ = 7.8 Hz, $^{3}J_{HH}$ = 4.8 Hz, 1H, H_d), 2.20 (s, 3H, H_e).

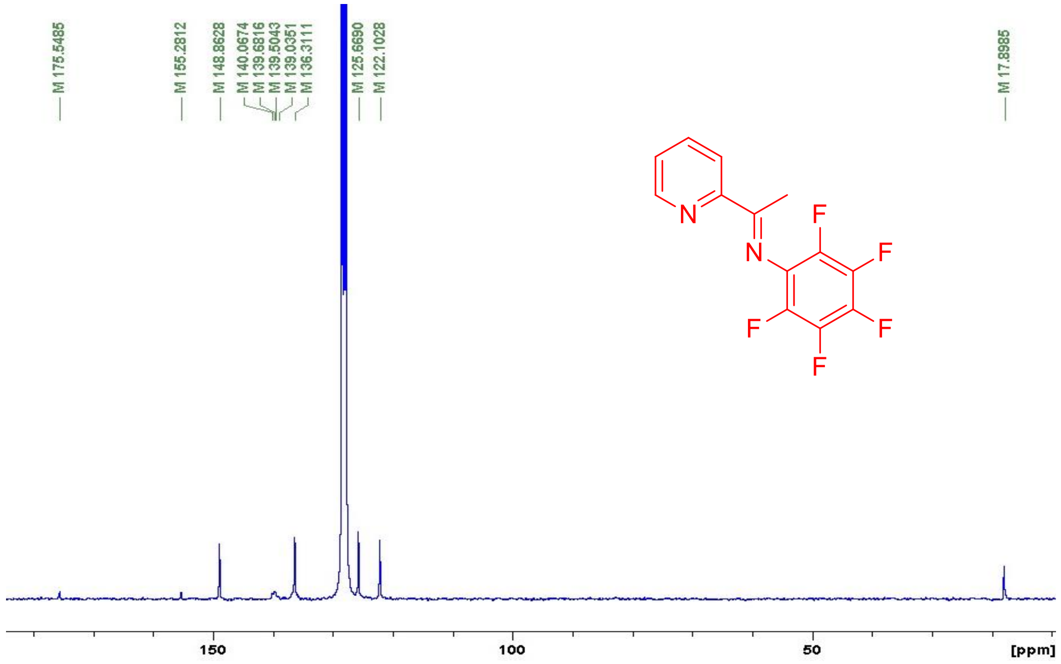

Figure S2. ^{13}C NMR of **L6** (75 MHz, C_6D_6, 25 °C)

^{13}C NMR (75 MHz, C_6D_6, 25 °C) δ (ppm) = 175.1, 154.9, 148.5, 139.7, 139.3, 139.1, 138.7, 135.9, 125.4, 121.8, 17.5. At this stage, we could not assign the ^{13}C NMR spectrum

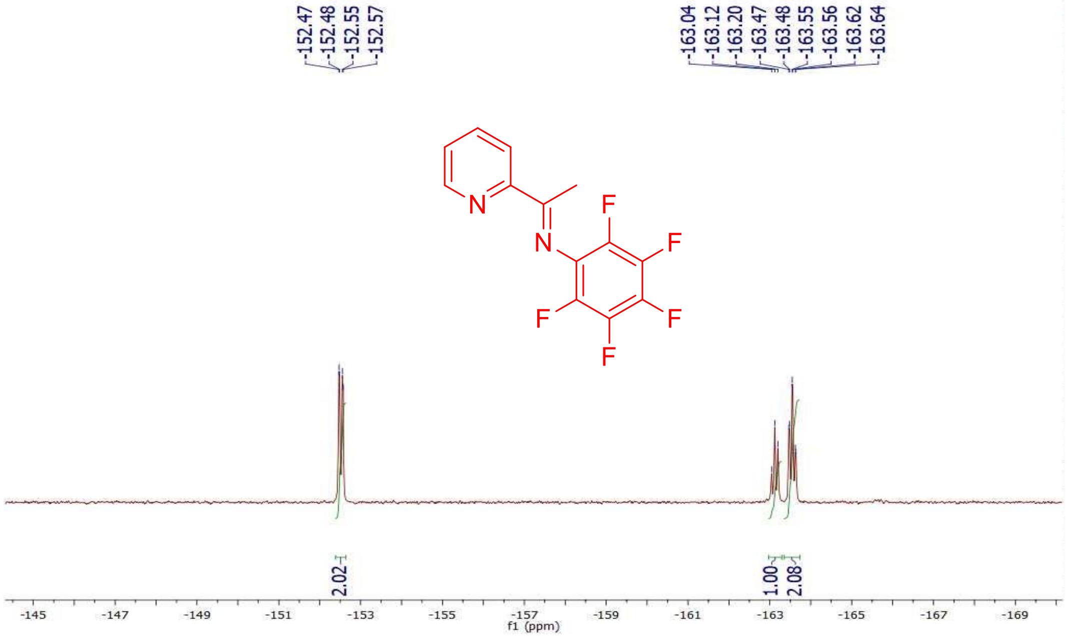

Figure S3. ^{19}F NMR of **L6** (282 MHz, C_6D_6, 25 °C)

^{19}F NMR (282 MHz, C_6D_6, 25 °C) δ (ppm) = -152.5 (d, *J* = 23.8 Hz, 2F, F_{meta}), -163.1 (t, *J* = 21.7 Hz, 1F, F_{para}), -163.6 (dd, *J* = 23.8, 21.7 Hz, 2F, F_{ortho}).

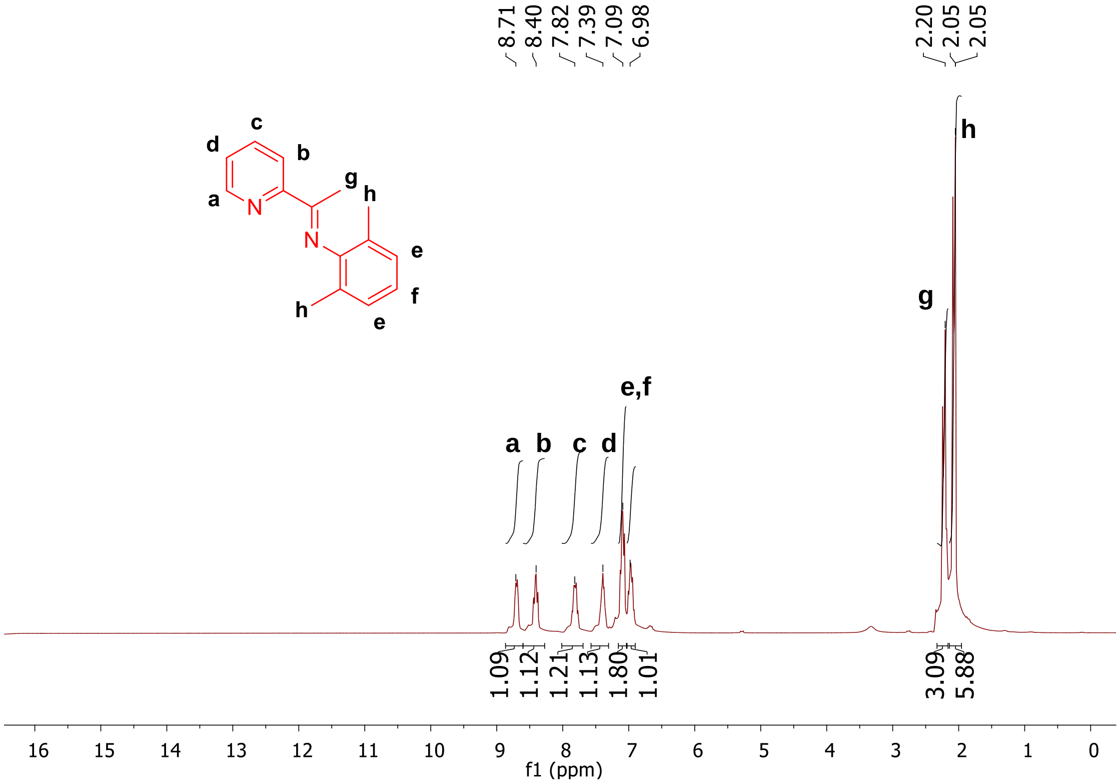

Figure S4. ^{1}H NMR of **L1** (300 MHz, $CDCl_3$, 25 °C)

^{1}H NMR of **L1** (300 MHz, $CDCl_3$, 25 °C) δ (ppm) = 8.71 (d broad, 1H, H_a), 8.40 (dd broad, 1H, H_b), 7.82 (dd broad, 1H, H_c), 7.39 (dd broad, 1H, H_d), 7.09-6.98 (m, 3H, $H_{e,f,}$), 2.20 (s, 3H, H_g), 2.05 (s, 6H, H_h). The ^{1}H NMR spectrum of L1 was poorly resolved; however, the chemical shifts and integrations of each proton are consistent with previous reported data.

Ref [33] *New J. Chem.* **2002**, *26* (4), 387–397: ^{1}H NMR ($CDCl_3$): δ (ppm) = 8.69 (ddd, ^3J(HH) = 4.9, ^4J(HH) = 1.8, ^5J(HH) = 0.9 Hz, 1H, $H_{6\text{-py}}$), 8.30 (ddd, ^3J(HH) = 7.7, ^4J(HH) = 1.3, ^5J(HH) = 0.9 Hz, 1H, $H_{3\text{-py}}$), 7.83 (td, ^3J(HH) = 7.7, ^4J(HH) = 1.8 Hz, 1H, $H_{4\text{-py}}$), 7.40 (ddd, ^3J(HH) = 7.7, 4.9, ^4J(HH) = 1.3 Hz, 1H, $H_{5\text{-py}}$), 6.91–7.10 (m, 3H, H_{phenyl}), 2.20 (s, 3H, $H_{\text{bridge-Me}}$), 2.05 (s, 6H, H_{Me}).

Ref [34] *Org. Chem. Front.* **2014**, *1* (9), 1101–1106: ^{1}H NMR (400 MHz, $CDCl_3$) δ (ppm) = 8.68-8.66 (m, 1H, $H_{6\text{-py}}$), 8.38-8.36 (m, 1H, $H_{3\text{-py}}$), 7.82-7.78 (m, 1H, $H_{4\text{-py}}$), 7.39-7.36 (m, 1H, $H_{5\text{-py}}$), 7.06 (d, J = 7.2 Hz, 2H, H_{phenyl}), 6.93 (t, J = 7.6 Hz, 1H, H_{phenyl}), 2.19 (s, 3H, $H_{\text{bridge-Me}}$), 2.04 (s, 6H, H_{Me}).

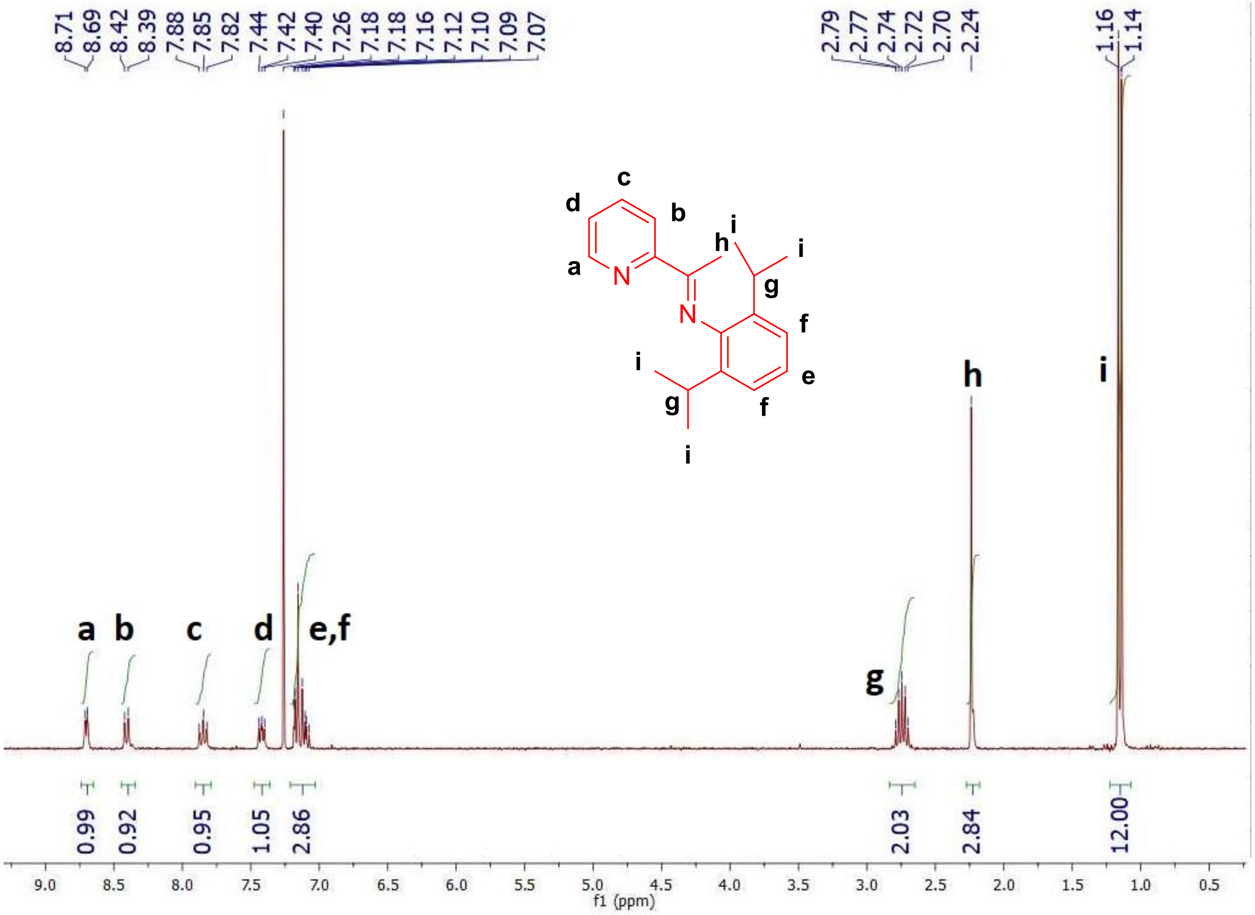

Figure S5. ^{1}H NMR of **L2** (300 MHz, $CDCl_3$, 25 °C)

^{1}H NMR of **L2** (300 MHz, $CDCl_3$, 25 °C) δ (ppm) = 8.70 (ddd, $^3J_{HH}$ = 4.8 Hz, $^4J_{HH}$ = 1.7 Hz, $^5J_{HH}$ = 0.8 Hz, 1H, H_a), 8.41 (d, $^3J_{HH}$ = 8.1 Hz, 1H, H_b), 7.85 (ddd, $^3J_{HH}$ = 8.1, 8.1 Hz, $^4J_{HH}$ = 1.7 Hz, 1H, H_c), 7.42 (ddd, $^3J_{HH}$ = 8.1 Hz, $^4J_{HH}$ = 4.8 Hz, $^5J_{HH}$ = 0.8 Hz, 1H, H_d), 7.21-7.03 (m, 3H, $H_{e,f}$), 2.74 (sept, $^3J_{HH}$ = 6.9 Hz, 2H, H_g), 2.24 (s, 3H, H_h), 1.15 (d, $^3J_{HH}$ = 6.9 Hz, 12H, H_i). The chemical shifts and integrations of each proton are consistent with previous reported data.

Ref [32] *J. Organomet. Chem.* **2000**, *606* (2), 112–124: ^{1}H NMR ($CDCl_3$) δ (ppm) = 8.69 (d, 1H, $H_{6\text{-}Py}$), 8.36 (d, 1H, $H_{3\text{-}Py}$), 7.82 (t, 1H, $H_{4\text{-}Py}$), 7.39 (m, 1H, $H_{5\text{-}Py}$), 7.11-7.20 (m, 3H, H_{phenyl}), 2.75 (m, 2H, C*H*Me_2), 2.21 (s, 3H, $H_{bridge\text{-}Me}$), 1.15 (d, 12H, H_{Me})

Ref [33] *New J. Chem.* **2002**, *26* (4), 387–397: ^{1}H NMR ($CDCl_3$) δ (ppm) = 8.70 (ddd, ^3J(HH) = 5.0, ^4J(HH) = 1.8, ^5J(HH) = 0.9 Hz, 1H, $H_{6\text{-}py}$), 8.37 (ddd, ^3J(HH) = 7.7, ^4J(HH) = 1.3, ^5J(HH) = 0.9 Hz, 1H, $H_{3\text{-}py}$), 7.83 (td, ^3J(HH) = 7.7, ^4J(HH) = 1.8 Hz, 1H, $H_{4\text{-}py}$), 7.41 (ddd, ^3J(HH) = 7.7, 5.0, ^4J(HH) = 1.3 Hz, 1H, $H_{5\text{-}py}$), 7.01– 7.21 (m, 3H, H_{phenyl}), 2.76 (sept., ^3J(HH) = 7.0 Hz, 2H, C*H*Me_2), 2.23 (s, 3H, $H_{bridge\text{-}Me}$), 1.16 (d, ^3J(HH) = 7.0 Hz, 12H, H_{Me}).

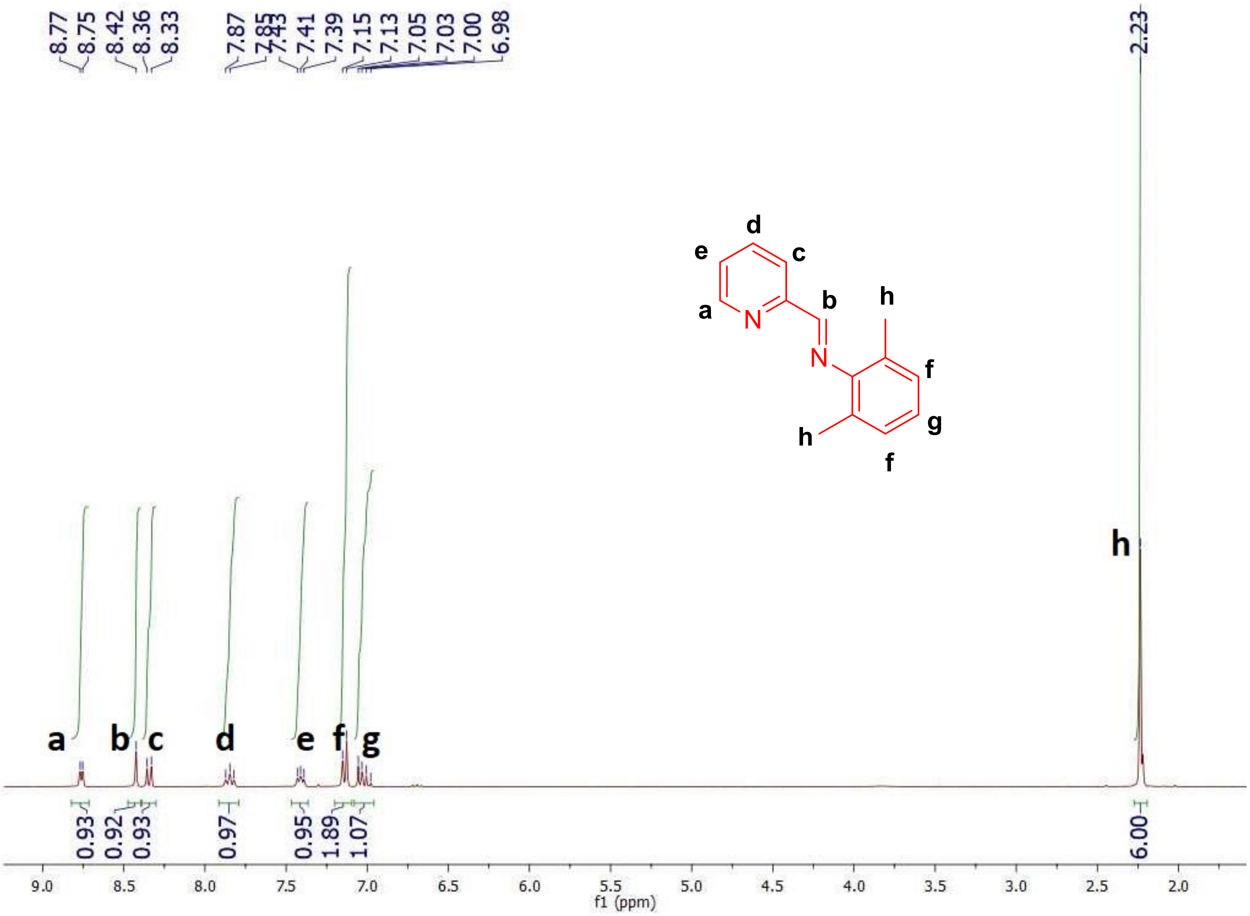

Figure S6. ^{1}H NMR of **L3** (300 MHz, $CDCl_3$, 25 °C)

^{1}H NMR of **L3** (300 MHz, $CDCl_3$, 25 °C) δ (ppm) = 8.76 (d, $^{3}J_{HH}$ = 4.7 Hz, 1H, H_a), 8.42 (s, 1H, H_b), 8.34 (d, $^{3}J_{HH}$ = 7.9 Hz, 1H, H_c), 7.85 (dd, $^{3}J_{HH}$ = 7.9, 7.9 Hz, 1H, H_d), 7.46-7.37 (m, 1H, H_e), 7.14-6.96 (m, 3H, $H_{f,g}$), 2.23 (s, 6H, H_h). The chemical shifts and integrations of each proton are consistent with previous reported data

Ref [33] *New J. Chem.* **2002**, *26* (4), 387–397: ^{1}H NMR ($CDCl_3$): δ (ppm) = 8.73 (ddd, ^{3}J(HH) = 4.9, ^{4}J(HH) = 1.8, ^{5}J(HH) = 1.0 Hz, 1H, $H_{6\text{-py}}$), 8.36 (s, 1H, C(H)=N), 8.30 (ddd, ^{3}J(HH) = 7.7, ^{4}J(HH) = 1.4, ^{5}J(HH) = 1.0 Hz, 1H, $H_{3\text{-py}}$), 7.85 (td, ^{3}J(HH) = 7.7, ^{4}J(HH) = 1.8 Hz, 1H, $H_{4\text{-py}}$), 7.42 (ddd, ^{3}J(HH) = 7.7, 4.9, ^{4}J(HH) = 1.4 Hz, 1H, $H_{5\text{-py}}$), 6.95–7.13 (m, 3H, H_{phenyl}), 2.18 (s, 6H, H_{Me}).

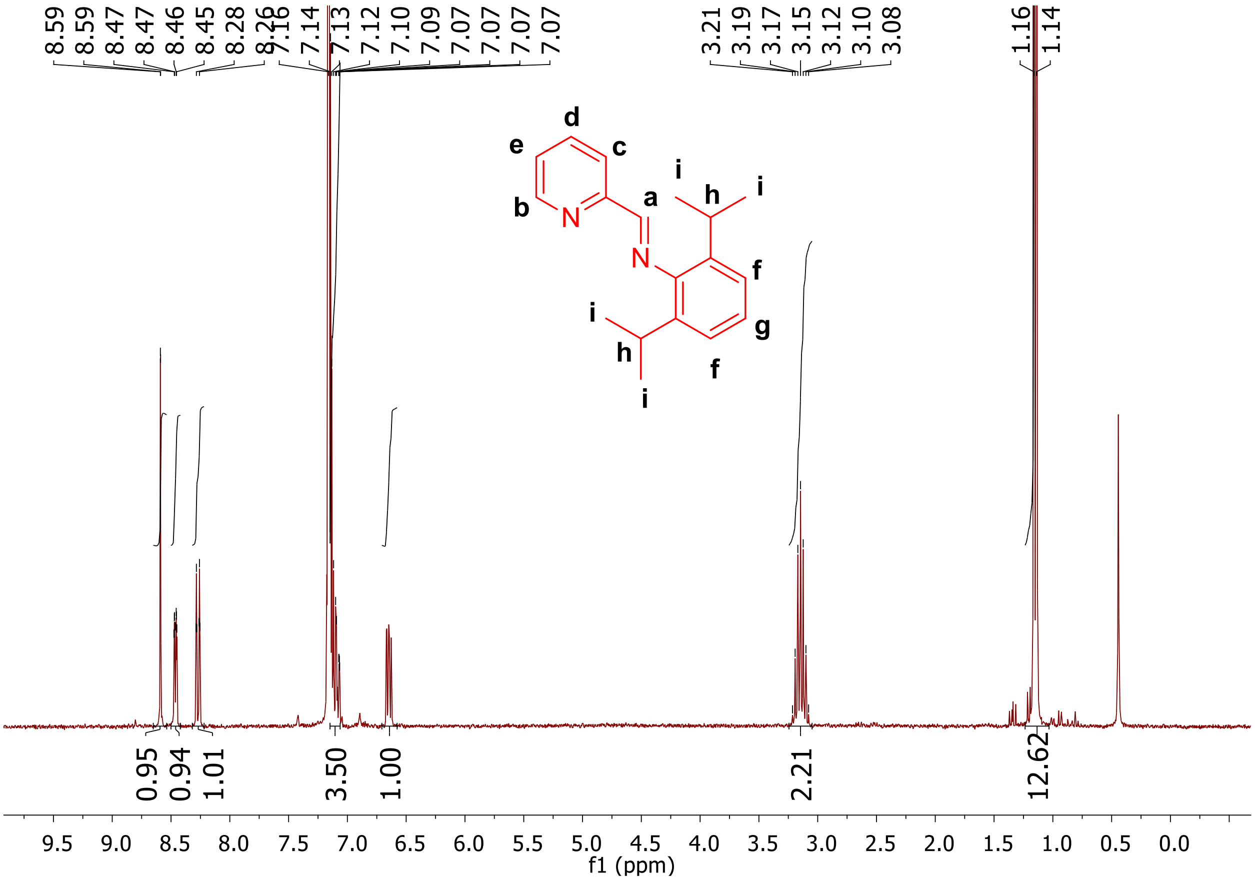

Figure S7. ^{1}H NMR of **L4** (300 MHz, C_6D_6, 25 °C)

^{1}H NMR of **L4** (300 MHz, C_6D_6, 25 °C) δ (ppm) = 8.59 (s, 1H, H_a), 8.46 (dd, $^3J_{HH}$ = 4.8 Hz, $^4J_{HH}$ = 1.7 Hz, 1H, H_b), 8.27 (d, $^3J_{HH}$ = 7.9 Hz, 1H, H_c), 7.13-7.04 (m, 4H, $H_{e,f,g}$), 6.65 (dd, $^3J_{HH}$ = 7.9, 4.8 Hz, 1H, H_d), 3.15 (sept, $^3J_{HH}$ = 6.8 Hz, 2H, H_h), 1.15 (d, $^3J_{HH}$ = 6.8 Hz, 12H, H_i). We encountered hydrolysis of the product when the ^{1}H NMR spectrum was performed in $CDCl_3$, probably due to the presence of traces of acid in the deuterated solvent. The integrations are consistent with the molecular formula of L4.

Ref [33] *New J. Chem.* **2002**, *26* (4), 387–39: ^{1}H NMR ($CDCl_3$): δ (ppm) = 8.73 (ddd, ^3J(HH) = 5.0, ^4J(HH) = 1.8, ^5J(HH) = 0.9 Hz, 1H, $H_{6\text{-py}}$), 8.31 (s, 1H, C(H)=N), 8.27 (ddd, ^3J(HH) = 7.7, ^4J(HH) = 1.3, ^5J(HH) = 0.9 Hz, 1H, $H_{3\text{-py}}$], 7.86 (td, ^3J(HH) = 7.7, ^4J(HH) = 1.8 Hz, 1H, $H_{4\text{-py}}$), 7.42 (ddd, ^3J(HH) = 7.7, 5.0, ^4J(HH) = 1.3 Hz, 1H, $H_{5\text{-py}}$), 7.05–7.23 (m, 3H, H_{phenyl}), 2.97 (sept., ^3J(HH) = 7.0 Hz, 2H, C*H*Me$_2$), 1.16 (d, ^3J(HH) = 7.0 Hz, 12H, H_{Me}).

Ref [35] *Eur. J. Inorg. Chem.* **1999**, 959-964: ^{1}H NMR ($CDCl_3$): δ (ppm) = 1.17 (d, 12 H, H_{Me}), 2.97 (m, 2 H, C*H*Me$_2$), 7.16 (m, 3 H, H_{phenyl}), 7.42 (m, 1 H, $H_{5\text{-py}}$), 7.86 (t, 1 H, $H_{4\text{-py}}$), 8.27 (d, 1 H, $H_{3\text{-py}}$), 8.31 (s, 1 H, C(H)=N), 8.73 (d, 1 H, $H_{6\text{-py}}$).

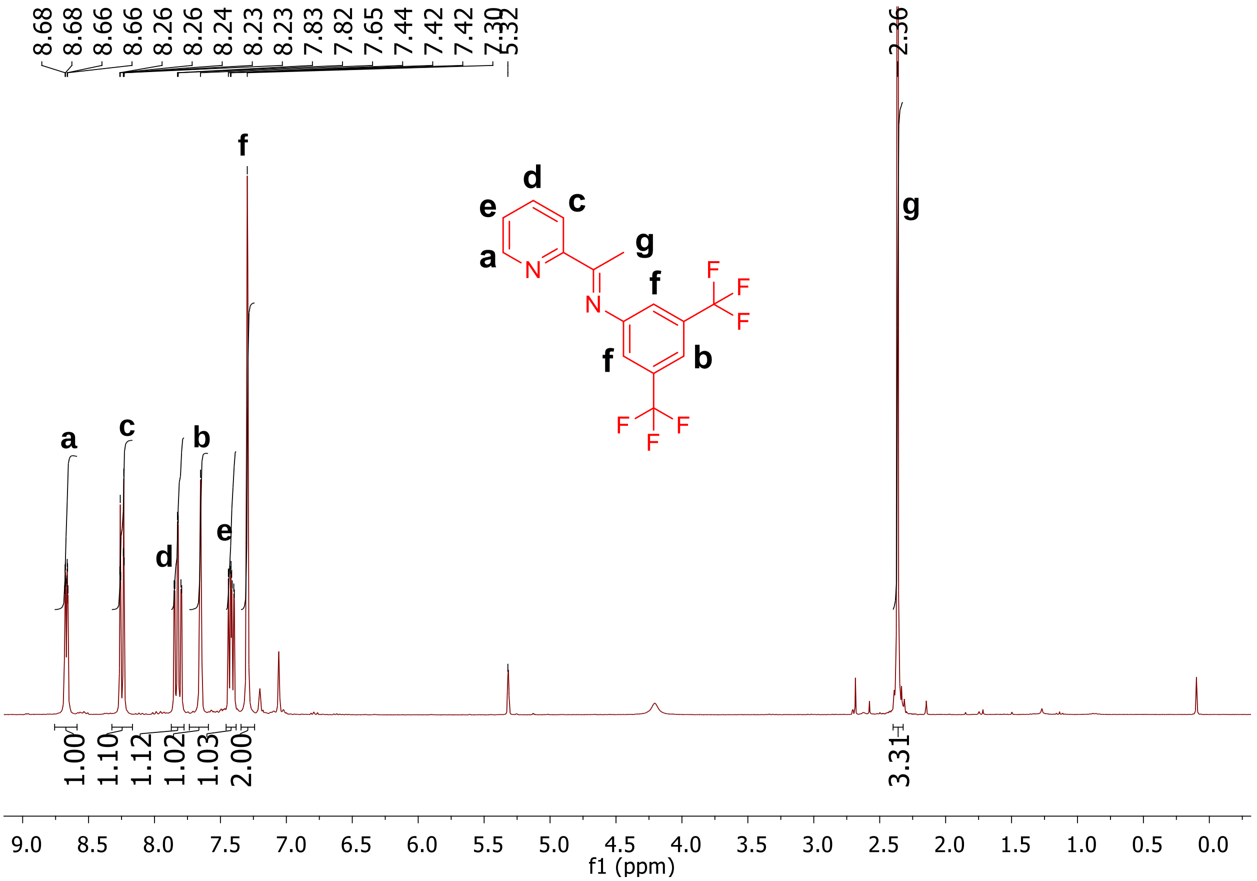

Figure S8. ^{1}H NMR of **L5** (300 MHz, CD_2Cl_2, 25 °C)

^{1}H NMR of **L5** (300 MHz, CD_2Cl_2, 25 °C) δ (ppm) = 8.67 (dd, $^3J_{HH}$ = 4.8 Hz, $^4J_{HH}$ = 1.7 Hz, 1H, H_a), 8.25 (d, $^3J_{HH}$ = 7.8 Hz, 1H, H_c), 7.83 (dd, J = 7.8, 7.8 Hz, 1H, H_d), 7.65 (s, 1H, H_b), 7.42 (dd, $^3J_{HH}$ = 7.8, 4.8 Hz, 1H, H_e), 7.30 (s, 2H, H_f), 2.37 (s, 3H, H_g). We encountered hydrolysis of the product when the ^{1}H NMR spectrum was performed in $CDCl_3$, probably due to the presence of traces of acid in the deuterated solvent. The integrations are consistent with the molecular formula of L5.

Ref [36] *Angew. Chem. Int. Ed.* **2018**, *57* (37), 12111–12115: ^{1}H NMR (300 MHz, $CDCl_3$) δ (ppm) = 8.69 (d, J = 4.5 Hz, 1H, $H_{6\text{-}pyridine}$), 8.24 (d, J = 8.1 Hz, 1H, H_{phenyl}), 7.90-7.74 (m, 1H, $H_{3\text{-}pyridine}$), 7.63 (s, 1H, $H_{4\text{-}pyridine}$), 7.48-7.33 (m, 1H, $H_{5\text{-}pyridine}$), 7.27 (s, 2H, H_{phenyl}), 2.39 (s, 3H, $H_{bridge\text{-}Me}$);

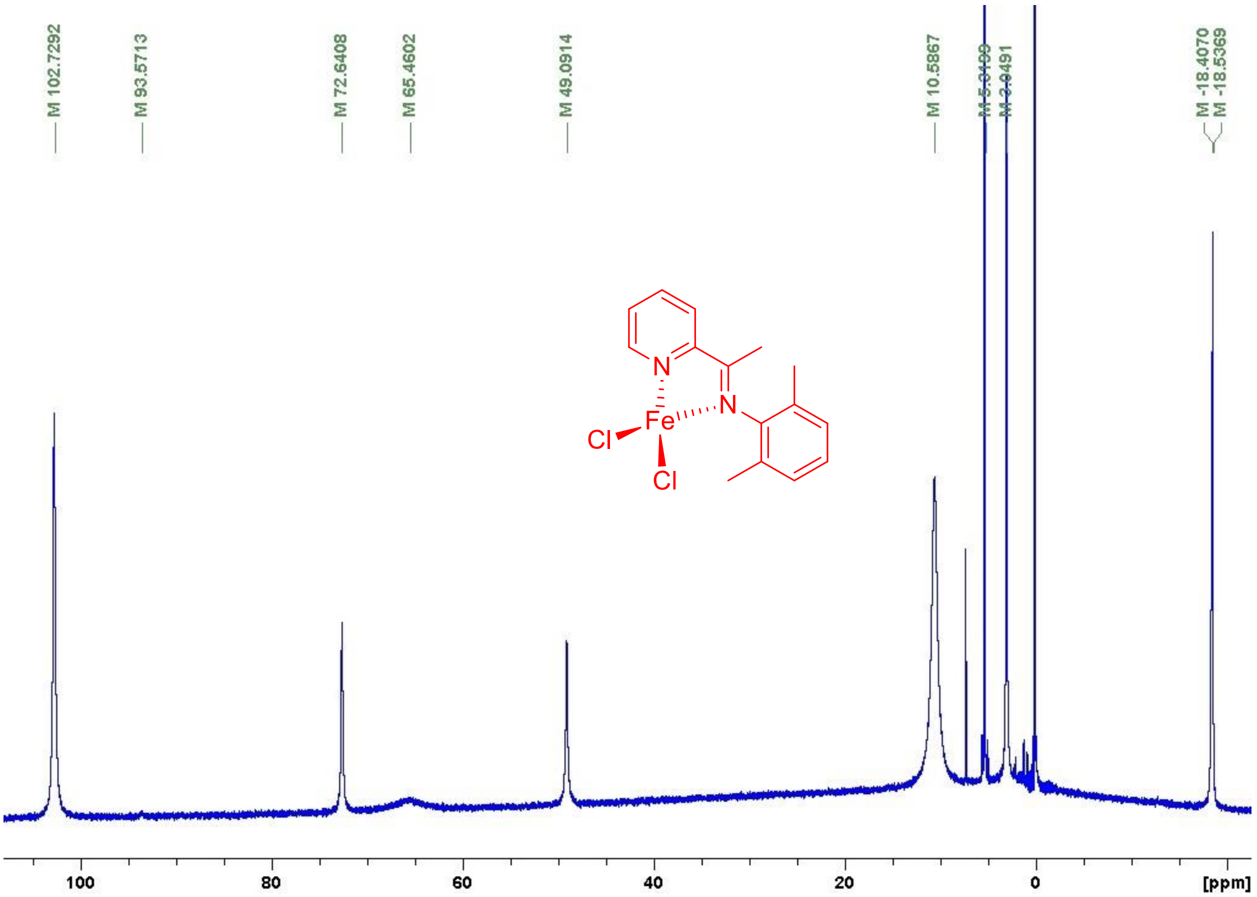

Figure S9. ^{1}H NMR spectrum of complex **C1** (300 MHz, CD_2Cl_2, 25 °C)

δ (ppm) = 102.7 ($\Delta\nu_{1/2}$ = 80 Hz, 3H), 72.6 ($\Delta\nu_{1/2}$ = 54 Hz, 1H), 65.5 ($\Delta\nu_{1/2}$ = 1089 Hz, 1H), 49.1 ($\Delta\nu_{1/2}$ = 61 Hz, 1H), 10.6 ($\Delta\nu_{1/2}$ = 194 Hz, 6H), 3.0 ($\Delta\nu_{1/2}$ = 28 Hz, 2H), -18.4 (2H).

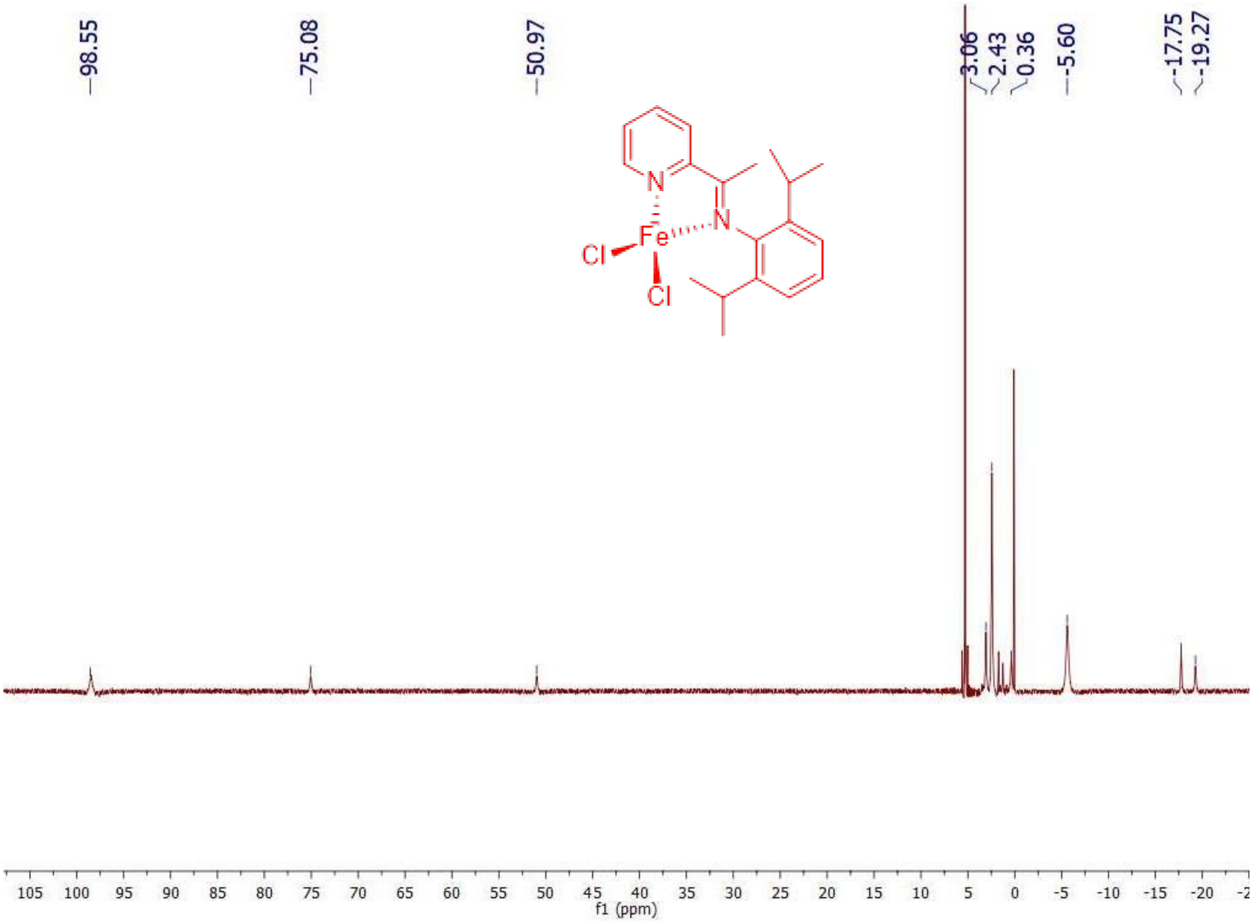

Figure S10. ^{1}H NMR spectrum of complex **C2** (300 MHz, CD_2Cl_2, 25 °C)

δ (ppm) = 99.0 ($\Delta\nu_{1/2}$ = 134 Hz, 1H), 75.1 ($\Delta\nu_{1/2}$ = 55 Hz, 1H), 51.0 ($\Delta\nu_{1/2}$ = 48 Hz, 1H), 3.1 ($\Delta\nu_{1/2}$ = 34 Hz, 3H), 2.4 ($\Delta\nu_{1/2}$ = 28 Hz, 12H), 0.4 ($\Delta\nu_{1/2}$ = 39 Hz, 1H), -5.6 ($\Delta\nu_{1/2}$ = 94 Hz, 2H), -17.8 ($\Delta\nu_{1/2}$ = 29 Hz, 2H), -19.3 ($\Delta\nu_{1/2}$ = 42 Hz, 1H).

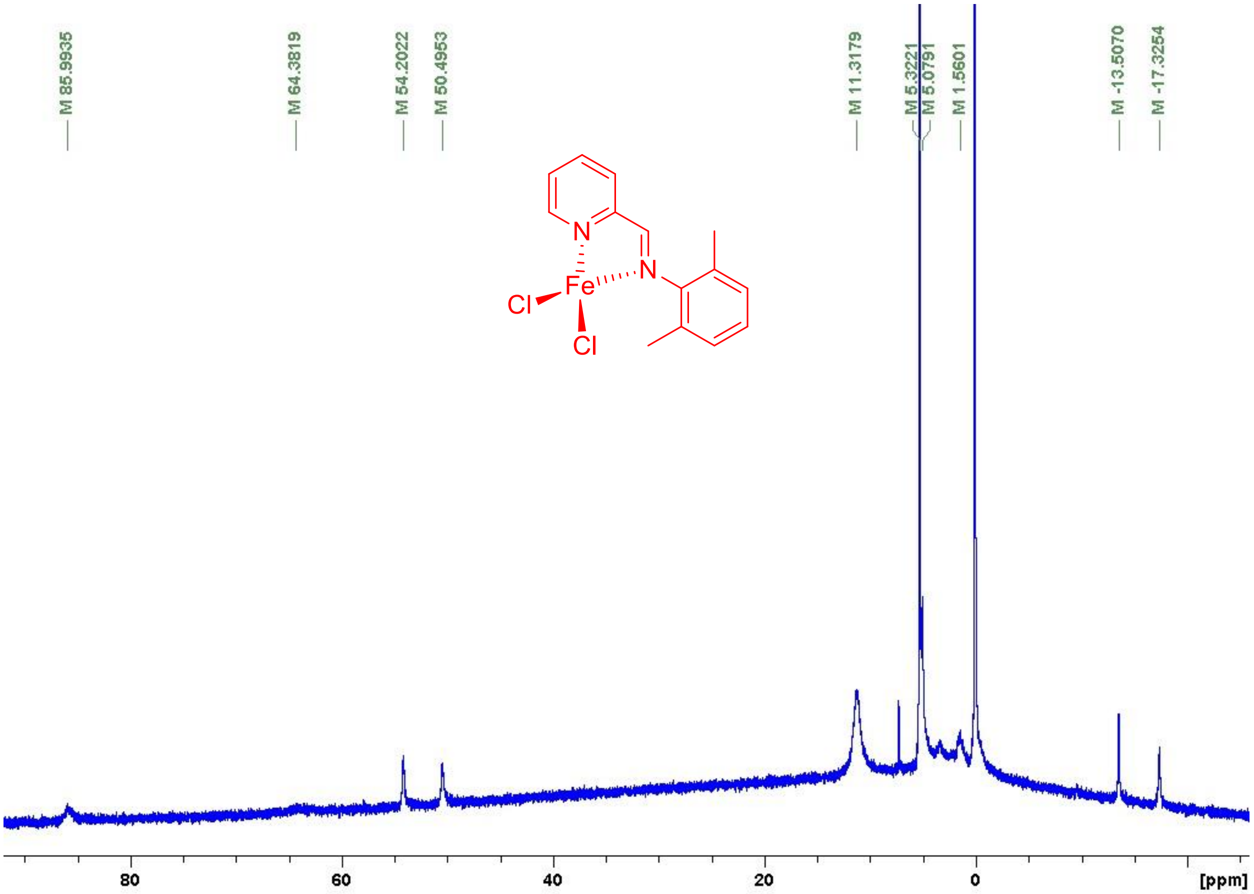

Figure S11. ^{1}H NMR spectrum of complex **C3** (300 MHz, CD_2Cl_2, 25 °C)

δ (ppm) = 85.9 ($\Delta\nu_{1/2}$ = 183 Hz, 1H), 64.4 ($\Delta\nu_{1/2}$ = 1526 Hz 1H), 54.2 ($\Delta\nu_{1/2}$ = 62 Hz, 1H), 50.5 ($\Delta\nu_{1/2}$ = 77 Hz, 1H), 11.3 ($\Delta\nu_{1/2}$ = 228 Hz, 6H), 5.1 (1H), -13.5 ($\Delta\nu_{1/2}$ = 31 Hz, 2H), -17.3 ($\Delta\nu_{1/2}$ = 47 Hz, 1H).

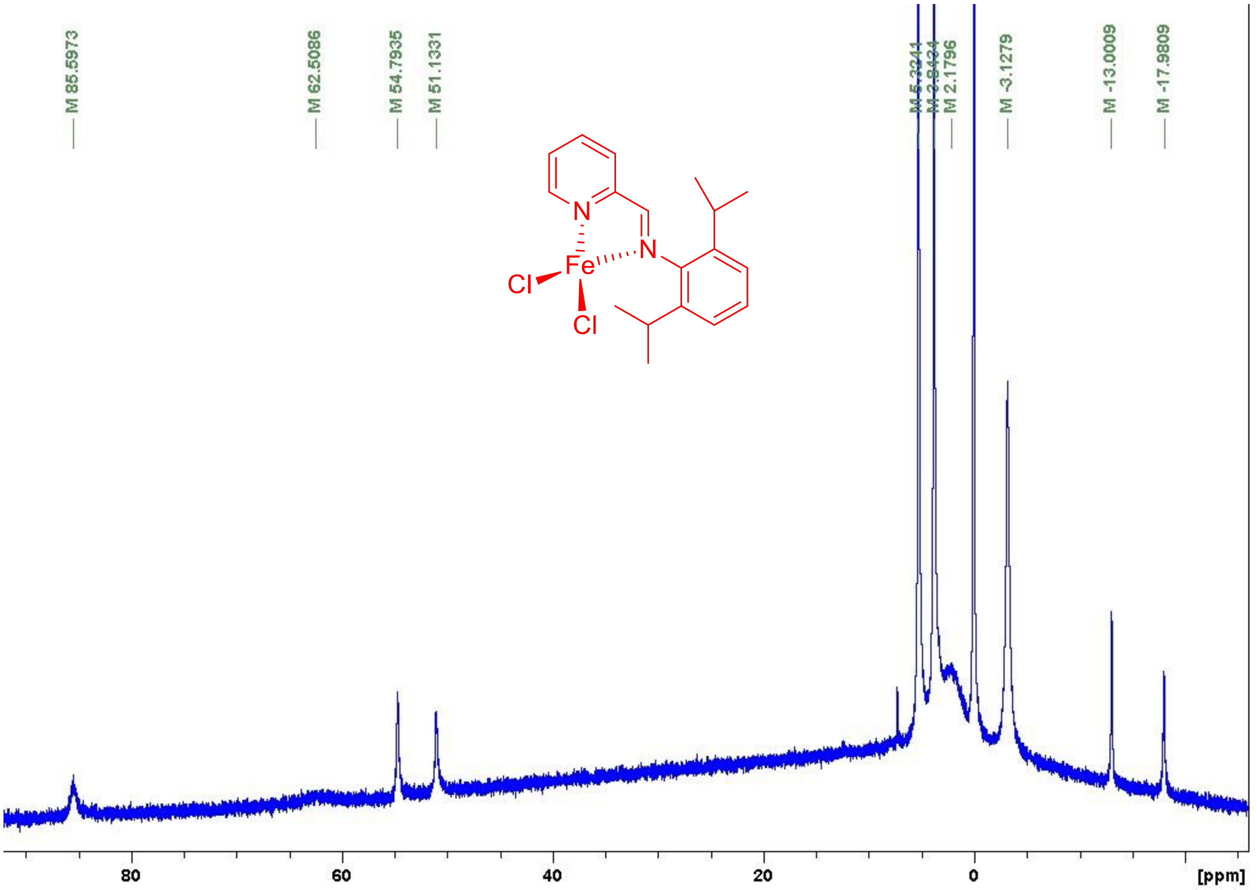

Figure S12. ^{1}H NMR spectrum of complex **C4** (300 MHz, CD_2Cl_2, 25 °C)

δ (ppm) = 85.6 ($\Delta\nu_{1/2}$ = 156 Hz, 1H), 62.5 ($\Delta\nu_{1/2}$ = 2300 Hz, 1H), 54.8 ($\Delta\nu_{1/2}$ = 61 Hz, 1H), 51.1 ($\Delta\nu_{1/2}$ = 75 Hz, 1H), 3.8 ($\Delta\nu_{1/2}$ = 50 Hz, 12H), 2.2 ($\Delta\nu_{1/2}$ = 447 Hz, 1H), -3.1 ($\Delta\nu_{1/2}$ = 95 Hz, 2H), -13.0 ($\Delta\nu_{1/2}$ = 34 Hz, 2H), -18.0 ($\Delta\nu_{1/2}$ = 48 Hz, 1H).

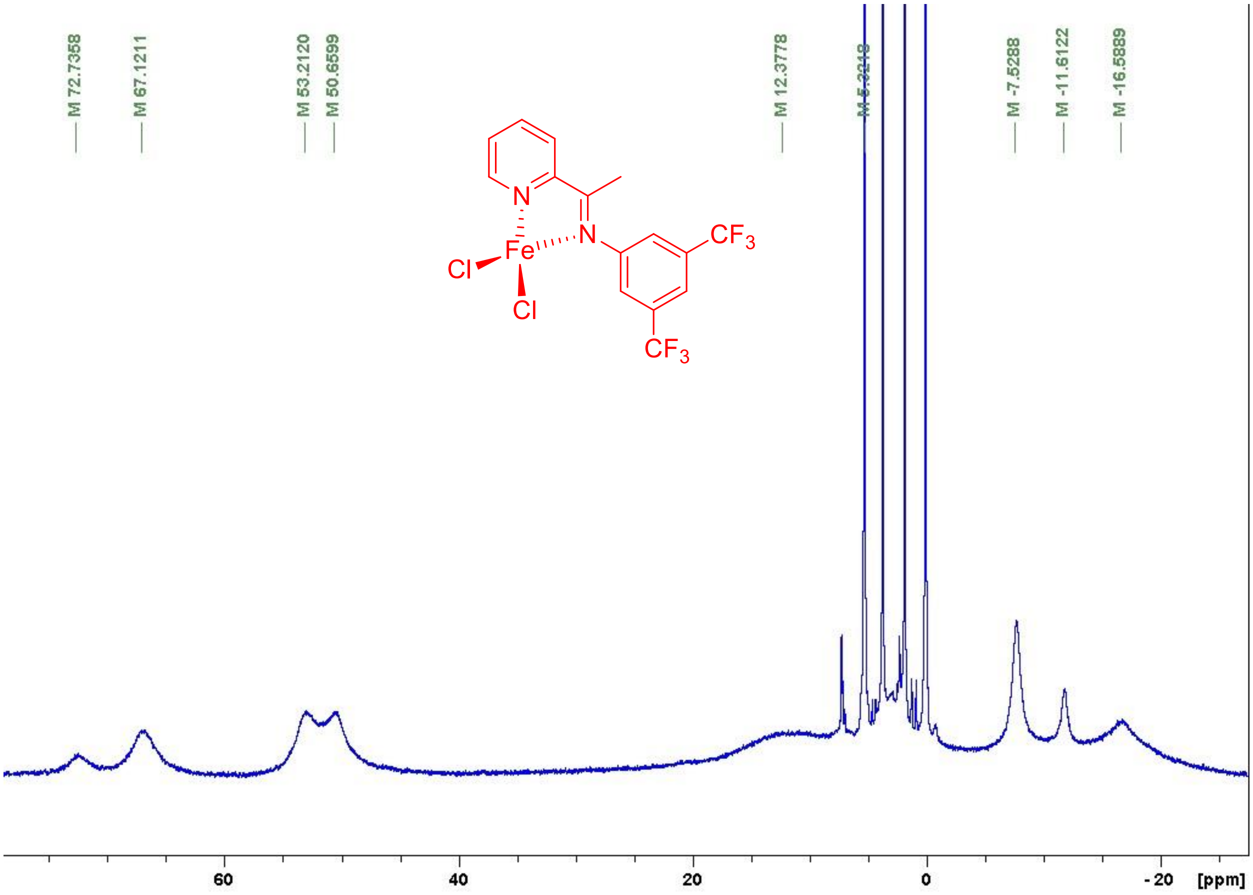

Figure S13. ^1H NMR spectrum of complex **C5** (300 MHz, CD_2Cl_2, 25 °C)

δ (ppm) = 72.7 ($\Delta\nu_{1/2}$ = 496 Hz, 1H), 67.1 ($\Delta\nu_{1/2}$ = 611 Hz, 1H), 53.2 & 50.65 (1H), 12.4 ($\Delta\nu_{1/2}$ = 1146 Hz, 1H), -7.5 ($\Delta\nu_{1/2}$ = 215 Hz, 3H), -11.6 ($\Delta\nu_{1/2}$ = 162 Hz, 1H), -16.6 ($\Delta\nu_{1/2}$ = 1711 Hz, 1H).

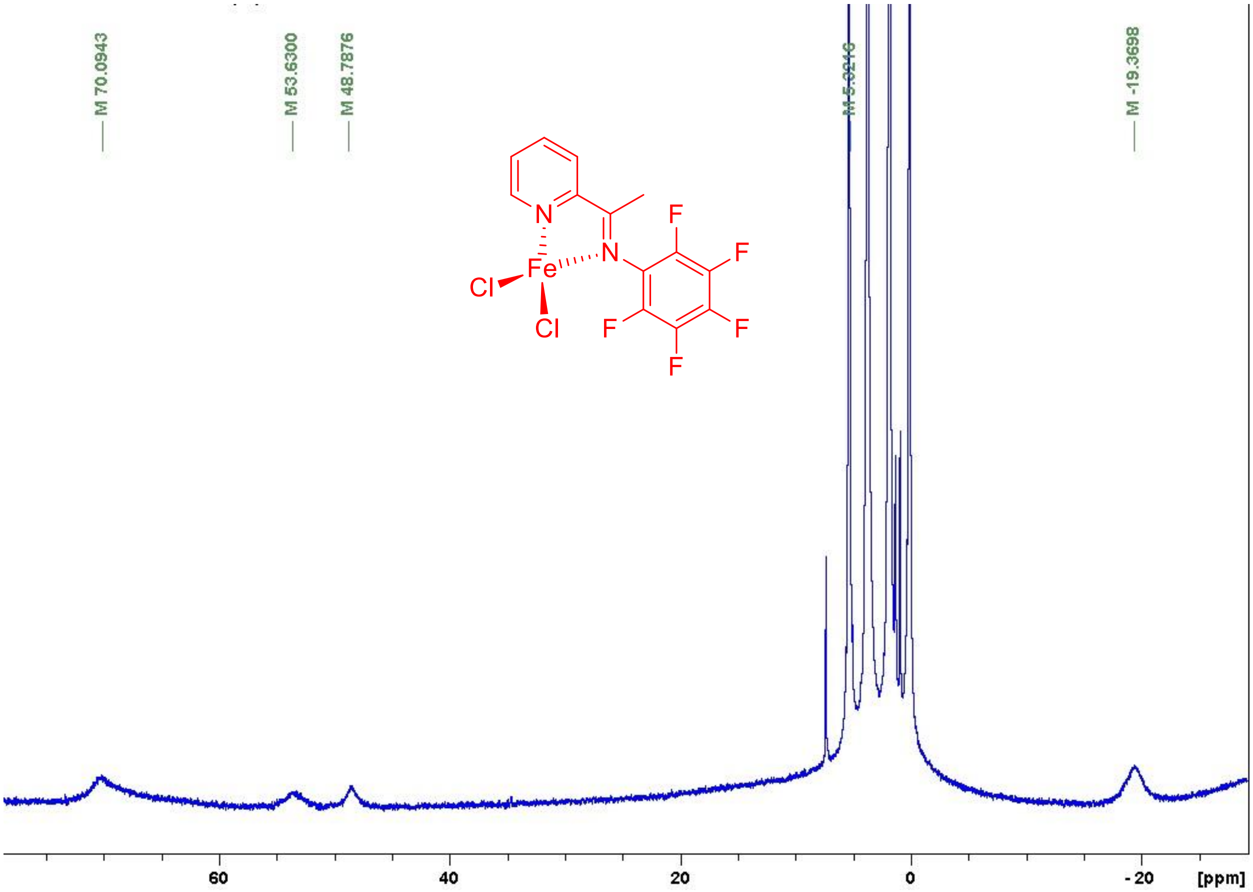

Figure S14. ^{1}H NMR spectrum of complex **C6** (300 MHz, CD_2Cl_2, 25 °C)

δ (ppm) = 70.1 ($\Delta\nu_{1/2}$ = 809 Hz, 2H), 53.6 ($\Delta\nu_{1/2}$ = 555 Hz, 1H), 48.8 ($\Delta\nu_{1/2}$ = 304 Hz, 1H), -19.4 ($\Delta\nu_{1/2}$ = 487 Hz, 3H).

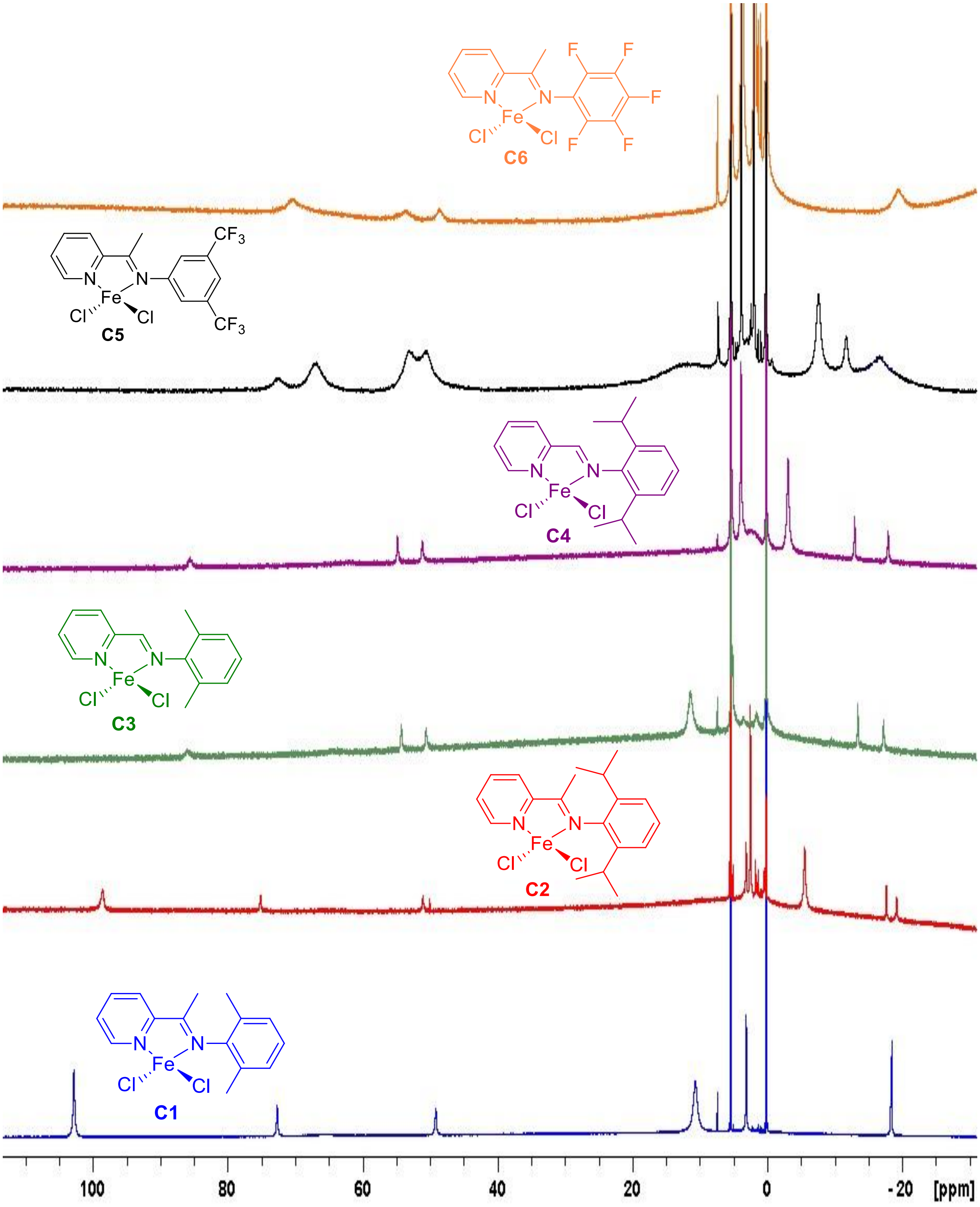

Figure S15. ^{1}H NMR spectrum of complexes **C1**- **C6** stacked (300 MHz, CD_2Cl_2, 25 °C)

Table S1. Polymerization of isoprene using **C1–C6**/$AlEt_3$/$[Ph_3C][B(C_6F_5)_4]$ (1/10/1) catalytic systems [a]

Entry	Complex	Conv. (%)	$M_{n(exp)}$ [b] (g/mol)	$Đ$ [b]	Microstructure [c] (%)	
					1,4 (*trans/cis*)	3,4
1	**C1**	>99	63,000[d]	1.6	90 (57/33)	10
2	**C2**	>99	19,000[d]	1.5	91 (79/12)	9
3	**C3**	>99	45,000	1.9	79 (25/54)	21
4	**C4**	>99	47,000	1.5	75 (32/43)	25
5	**C5**	>99	342,000	1.5	58 (0/58)	42
6	**C6**	>99	385,500	1.3	54 (0/54)	46

[a] Polymerization conditions: 10 µmol of Fe(II) complex; Isoprene/Fe/$AlEt_3$/$[Ph_3C][B(C_6F_5)_4]$ = 500/1/10/1; toluene = 5mL; time = 1 h; temperature = 25 °C; [b] determined by size exclusion chromatography (SEC); [c] determined by ^{1}H NMR and ^{13}C NMR; $M_{n(th)}$ = 33 700 g/mol (considering one growing chain per metal center); Activity = 34 x 10^3 $g_{(PI)}.mol_{(cat)}^{-1}$ h^{-1} or TOF = 500 h^{-1} for all; [d] contribution of a low amount (< 5 %) of a second fraction displaying high M_n

Table S2. Polymerization of isoprene using **C1–C6**/MAO (1/500) catalytic systems [a]

Entry	Complex	Conv. (%)	$M_{n(exp)}$ [b] (g/mol)	$Đ$ [b]	Microstructure [c] (%)	
					1,4 (*trans/cis*)	3,4
1	**C1**	>99	21,000	2.2	81 (50/31)	19
2	**C2**	>99	13,000[d]	1.3	91 (77/14)	9
3	**C3**	>99	33,500	1.9	89 (32/57)	11
4	**C4**	>99	29,000[d]	1.4	76 (30/46)	24
5	**C5**	>99	184,000	1.3	58 (0/58)	42
6	**C6**	>99	223,000[e]	1.8	54 (0/54)	46

[a] Polymerization conditions: 10 µmol of Fe(II) complex; Isoprene/Fe/MAO = 500/1/500; toluene = 5 mL; time = 1 h; temperature = 25 °C; [b] determined by size exclusion chromatography (SEC); [c] determined by ^{1}H NMR and ^{13}C NMR; $M_{n(the)}$ = 33,700 g/mol (considering one growing chain per metal center); Activity = 34 x 10^3 $g_{(PI)}.mol_{(cat)}^{-1}$ h^{-1} or TOF = 500 h^{-1} for all; [d] contribution of a low amount (< 5 %) of a second fraction displaying high M_n; [e] bimodal

Table S3. Polymerization of isoprene using **C1 – C6**/Al^iBu_3/$[Ph_3C][B(C_6F_5)_4]$ (1/3/1) catalytic systems[a]

Entry	Complex	Conv. (%)	Microstructure [b] (%)	
			1,4 (*trans/cis*)	3,4
1	**C1**	>99	91 (76/15)	9
2	**C2**	>99	92 (76/16)	8
3	**C3**	>99	78 (28/50)	22
4	**C4**	>99	75 (26/49)	25
5	**C5**	>99	59 (0/59)	41
6	**C6**	>99	54 (0/54)	46

[a] Polymerization conditions: 10 µmol of Fe(II) complex; Isoprene/Fe/$AlEt_3$/$[Ph_3C][B(C_6F_5)_4]$ = 500/1/3/1; toluene = 5mL; time = 1 h; temperature = 25 °C; [b] determined by ^{1}H NMR and ^{13}C NMR; Activity = 34 x 10^3 $g_{(PI)}.mol_{(cat)}^{-1}$ h^{-1} or TOF = 500 h^{-1} for all.

Table S4. Polymerization of isoprene (2,500 equiv./Fe) using **C4**/MAO (1/500) catalytic systems[a]

Entry	Complex	Conv. (%)	Microstructure [b] (%)	
			1,4 (*trans*/*cis*)	3,4
1	**C4**	> 99	73 (21/52)	27
Reference*	**C4**	83	74.4 (4.5/70)	25.6

[a] Polymerization conditions: 8 µmol of Fe(II) complex; Isoprene/Fe/MAO = 2 500/1/500; toluene = 7 mL and CH_2Cl_2 = 1 mL; time = 2 h; temperature = 25 °C; [b] determined by ^{1}H NMR and ^{13}C NMR; * ref. Guo, L.; Jing, X.; Xiong, S.; Liu, W.; Liu, Y.; Liu, Z.; Chen, C. Influences of Alkyl and Aryl Substituents on Iminopyridine Fe(II)- and Co(II)-Catalyzed Isoprene Polymerization. *Polymers* **2016**, *8* (11), 389

Table S5. Polymerization of 5,000 equiv. of isoprene/Fe using **C1 – C4**/Al^{i}Bu$_3$/[Ph$_3$C][B(C_6F_5)$_4$] catalytic systems[a]

Complex	Time (min)	Conversion[b] (%)	Complex	Time (min)	Conversion[b] (%)
**C1**	4	17	**C3**	1	21
	10	44		3	70
	15	59		6	82
	20	83		8	86
**C2**	5	3		10	89
	10	5	**C4**	2	15
	15	9		5	42
	30	12		7	60
	60	25		10	78

[a] : Polymerization conditions: 5 µmol of Fe(II) complex; Isoprene/Al^{i}Bu$_3$/[Ph$_3$C][B(C_6F_5)$_4$]/Fe = 5,000/3/1/1; toluene = 25 mL; temperature = 25 °C; [b] determined by ^{1}H NMR.

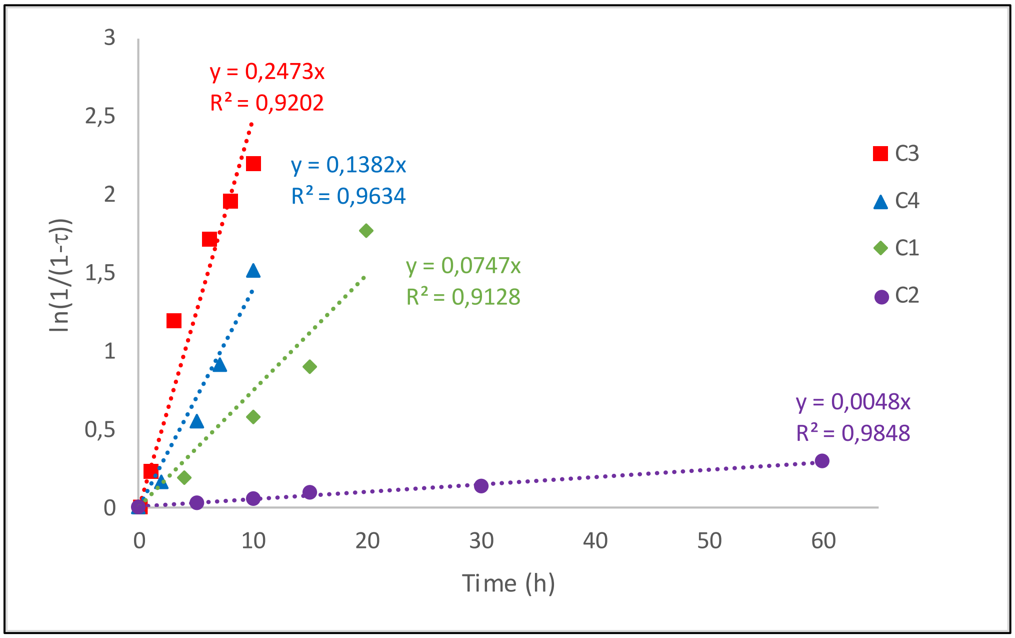

Figure S16. First-order kinetic plots for pre-catalysts **C1 – C4** (Isoprene/Al^{i}Bu$_3$/[Ph$_3$C][B(C_6F_5)$_4$]/Fe = 5,000/3/1/1).

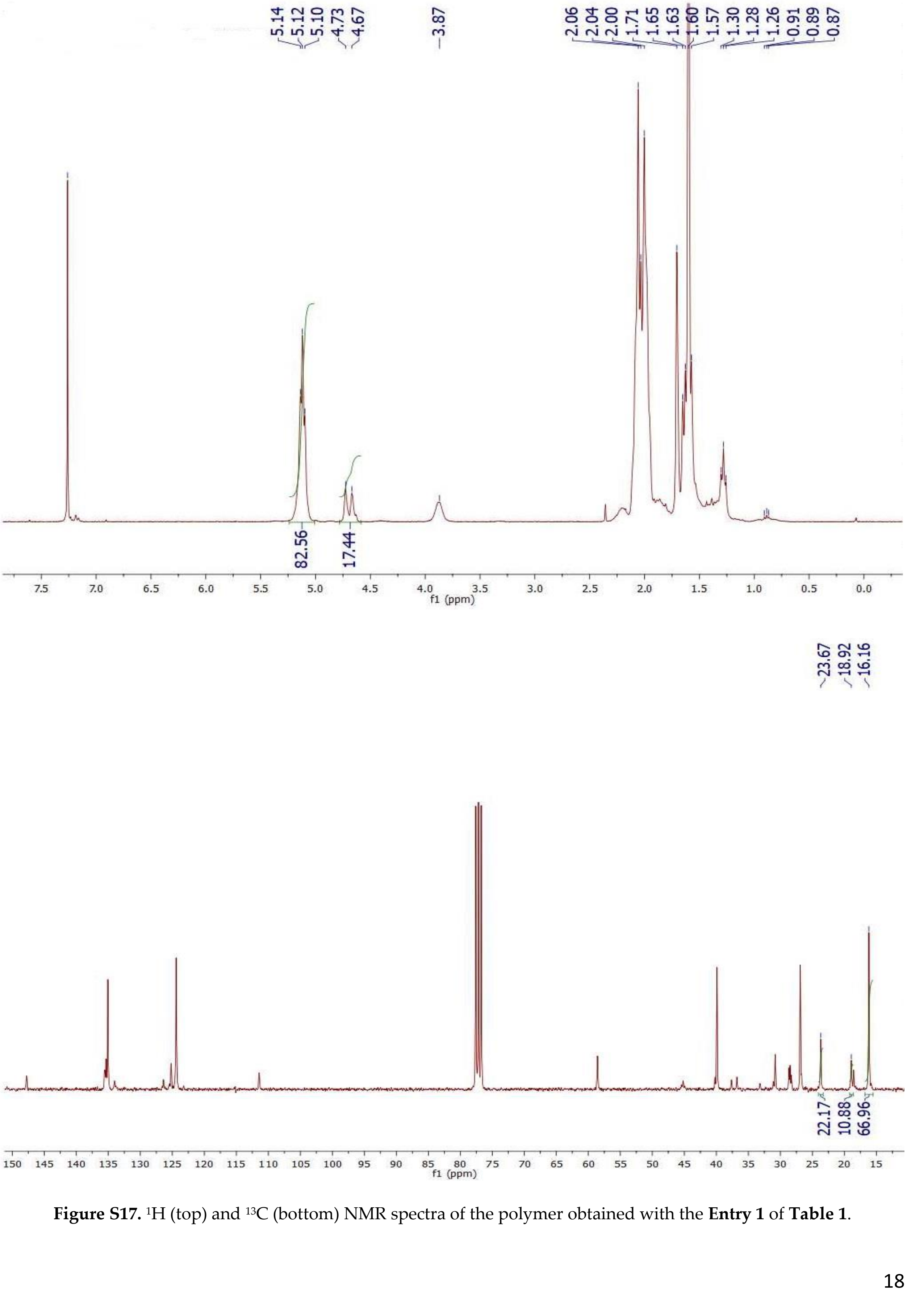

Figure S17. ^{1}H (top) and ^{13}C (bottom) NMR spectra of the polymer obtained with the **Entry 1** of **Table 1**.

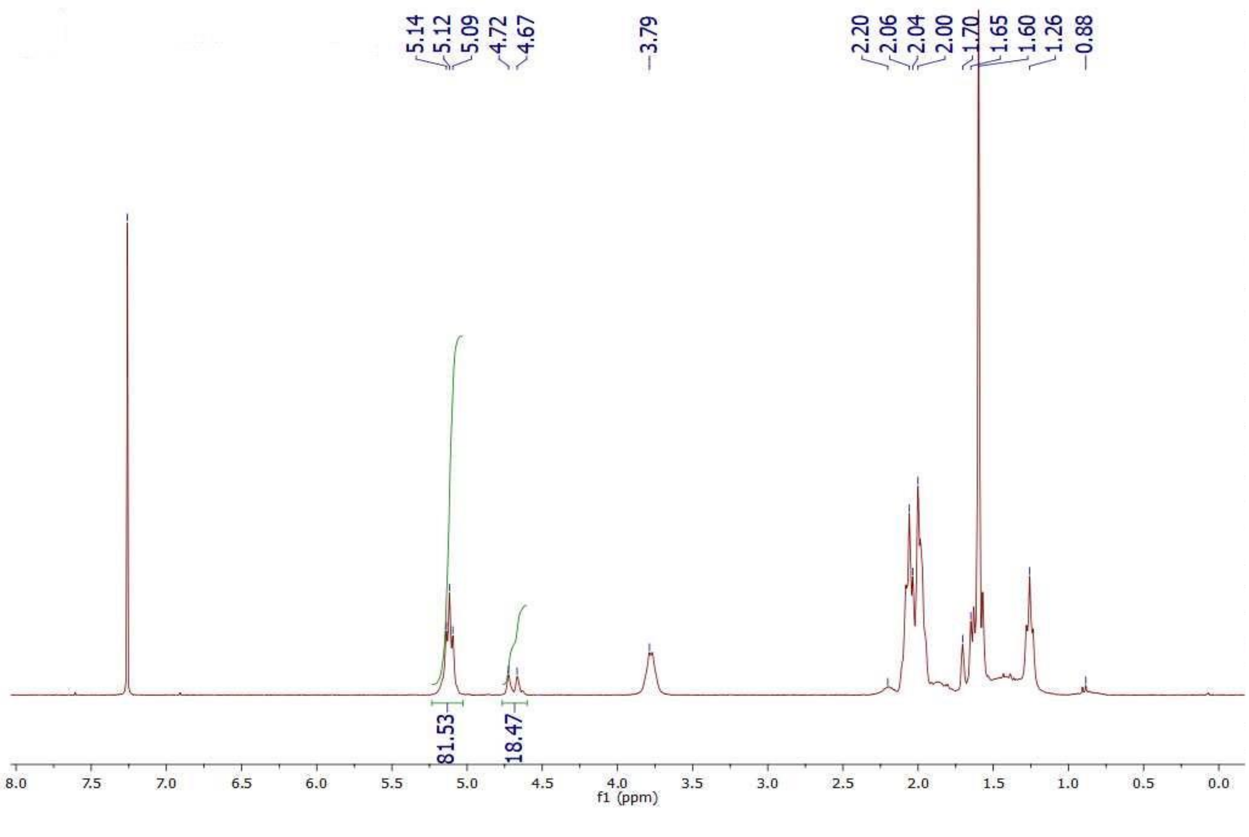

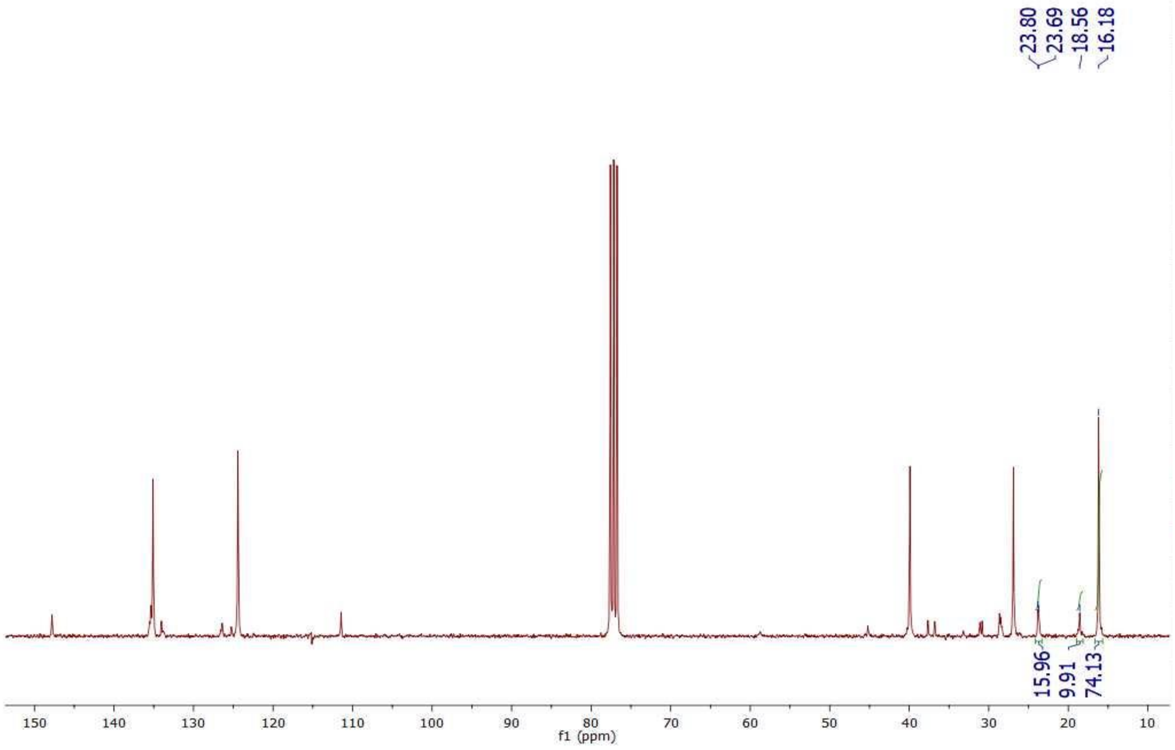

Figure S18. ^{1}H (top) and ^{13}C (bottom) NMR spectra of the polymer obtained with the **Entry 2** of **Table 1**.

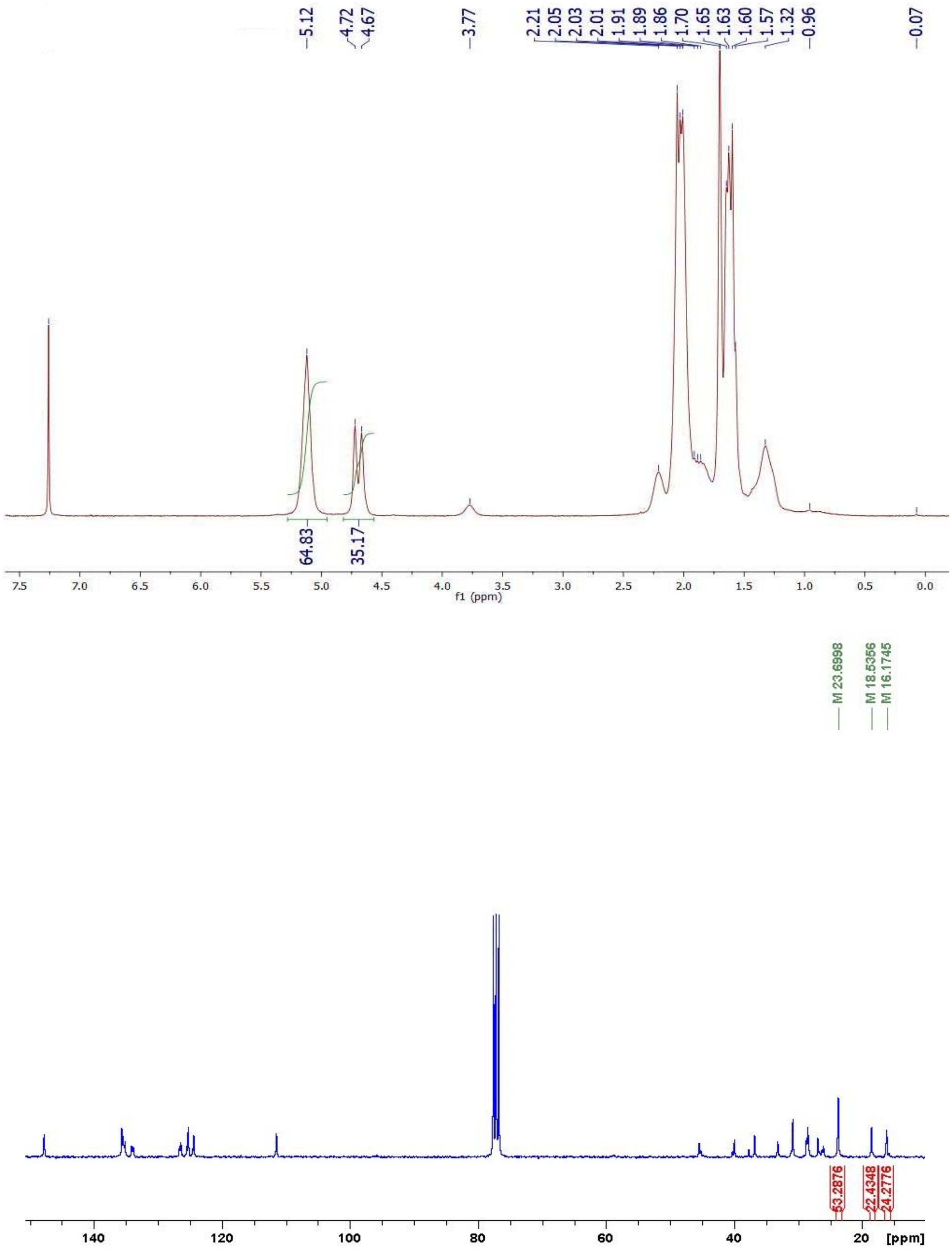

Figure S19. ^{1}H (top) and ^{13}C (bottom) NMR spectra of the polymer obtained with the **Entry 3** of **Table 1**.

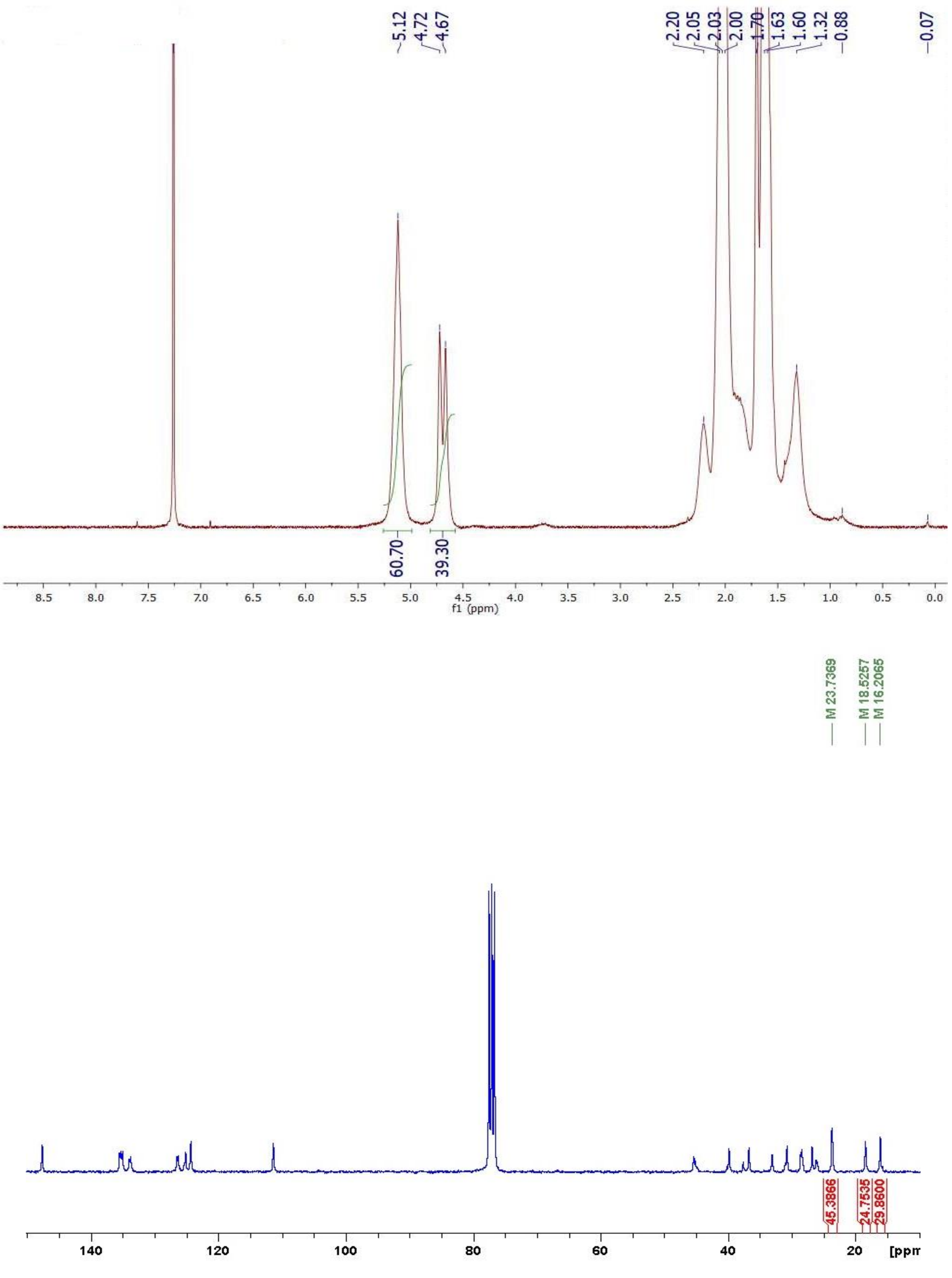

Figure S20. ^{1}H (top) and ^{13}C (bottom) NMR spectra of the polymer obtained with the **Entry 4** of **Table 1**.

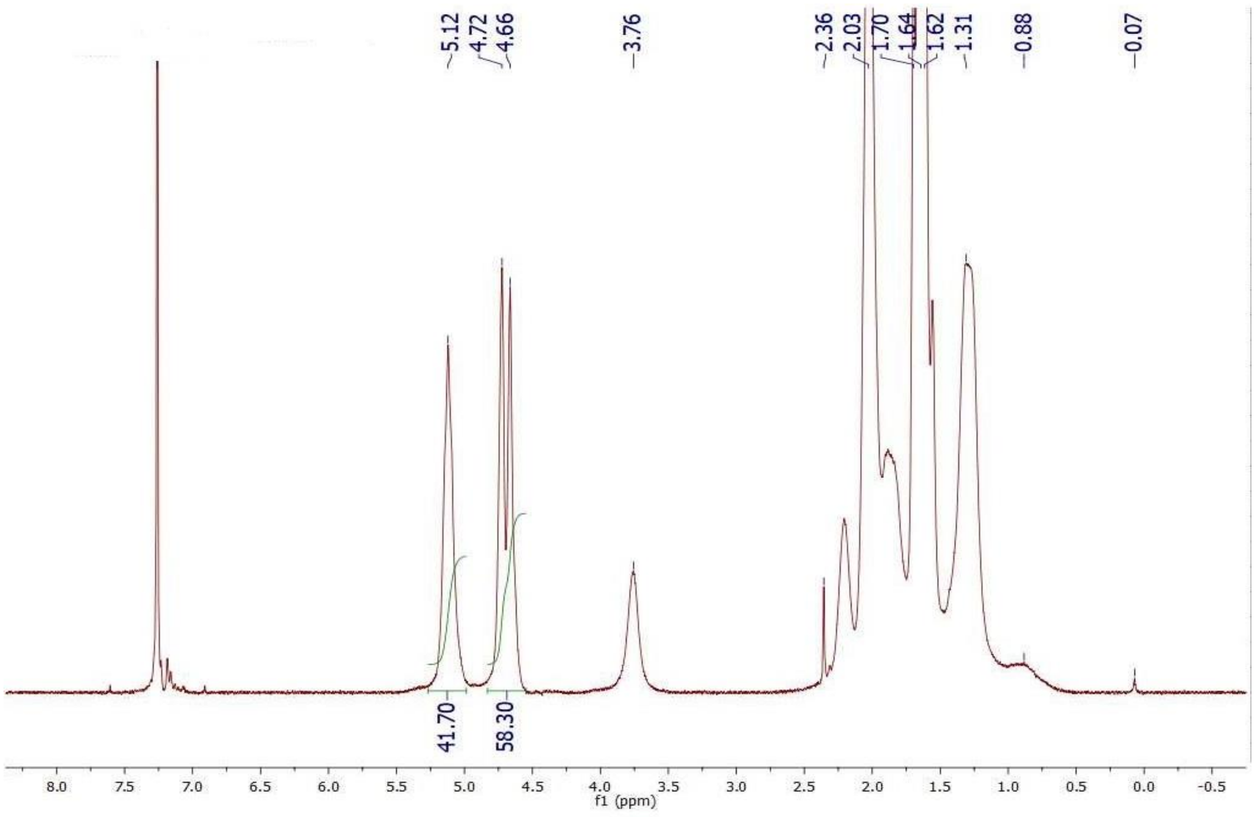

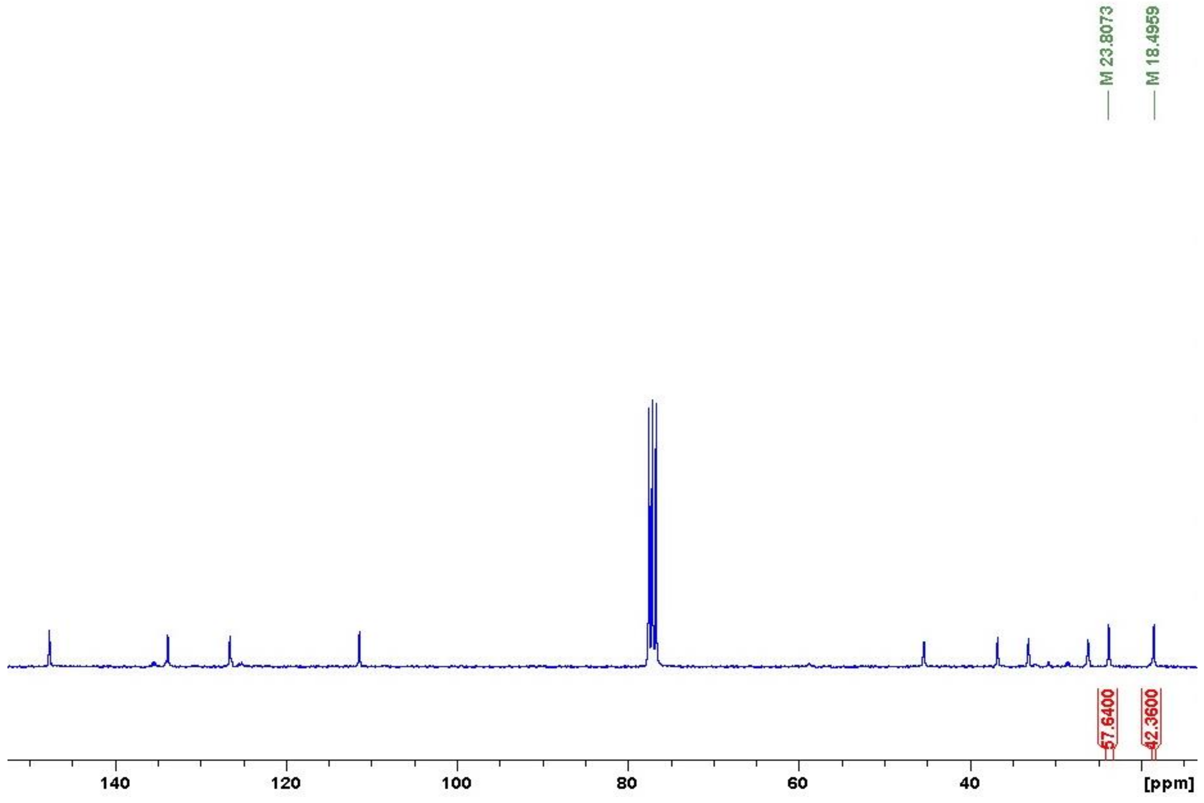

Figure S21. ¹H (top) and ¹³C (bottom) NMR spectra of the polymer obtained with the **Entry 5** of **Table 1**.

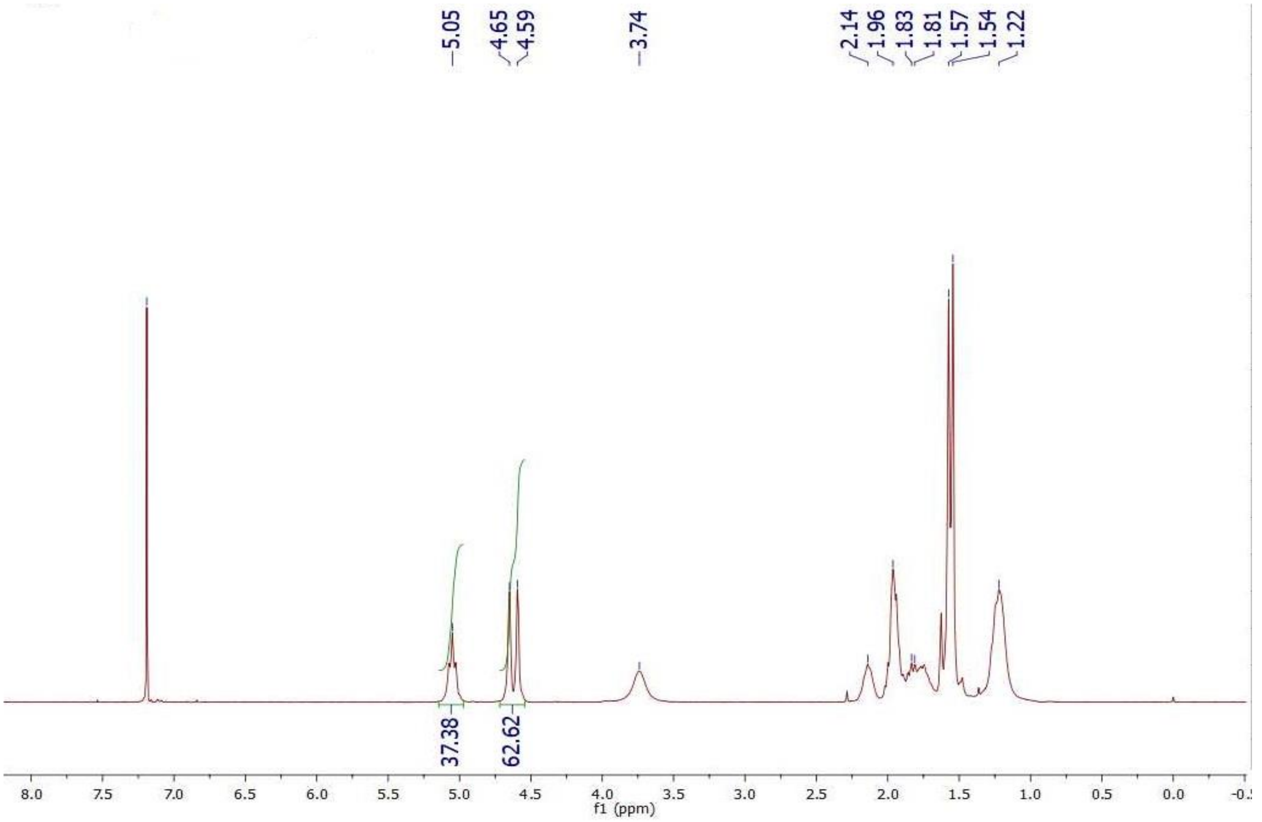

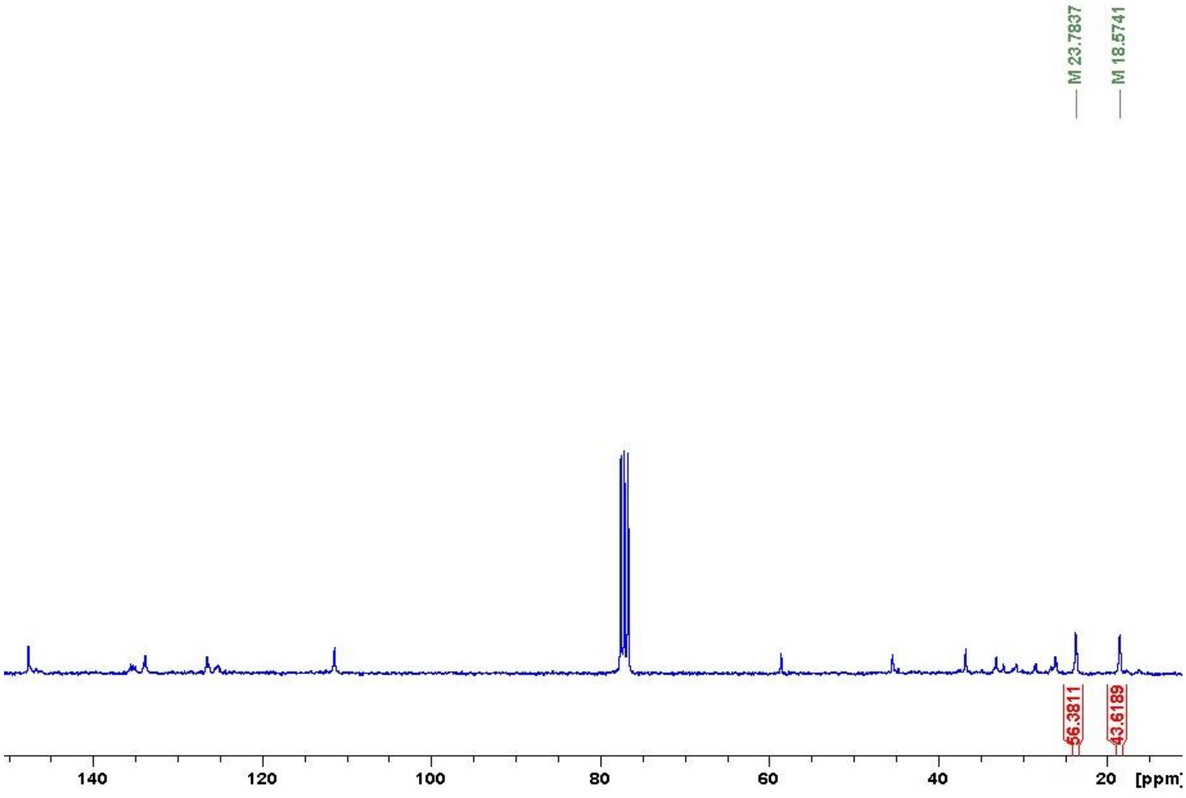

Figure S22. ^{1}H (top) and ^{13}C (bottom) NMR spectra of the polymer obtained with the **Entry 6** of **Table 1**.

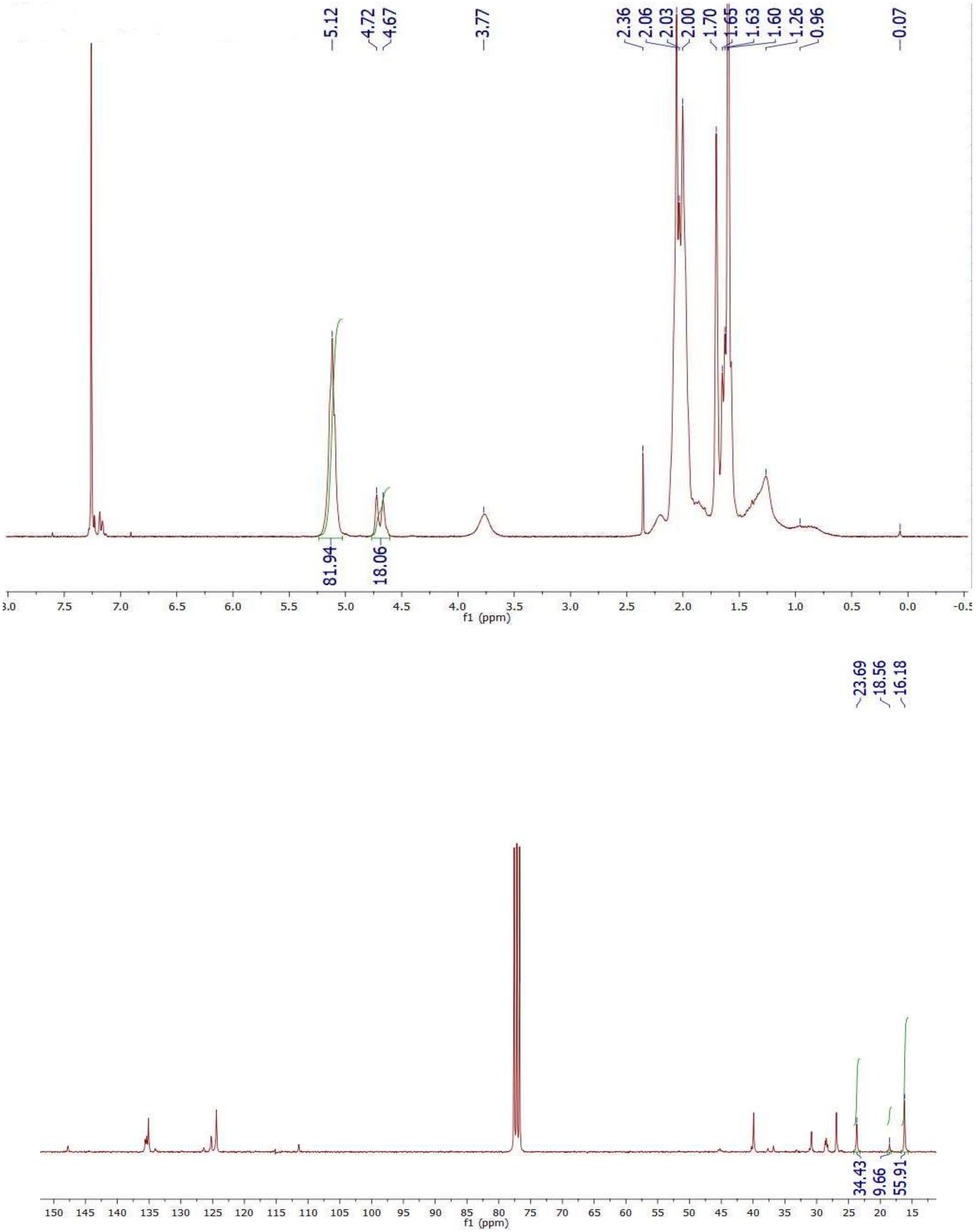

Figure S23. ^1H (top) and ^{13}C (bottom) NMR spectra of the polymer obtained with the **Entry 1** of **Table S1**.

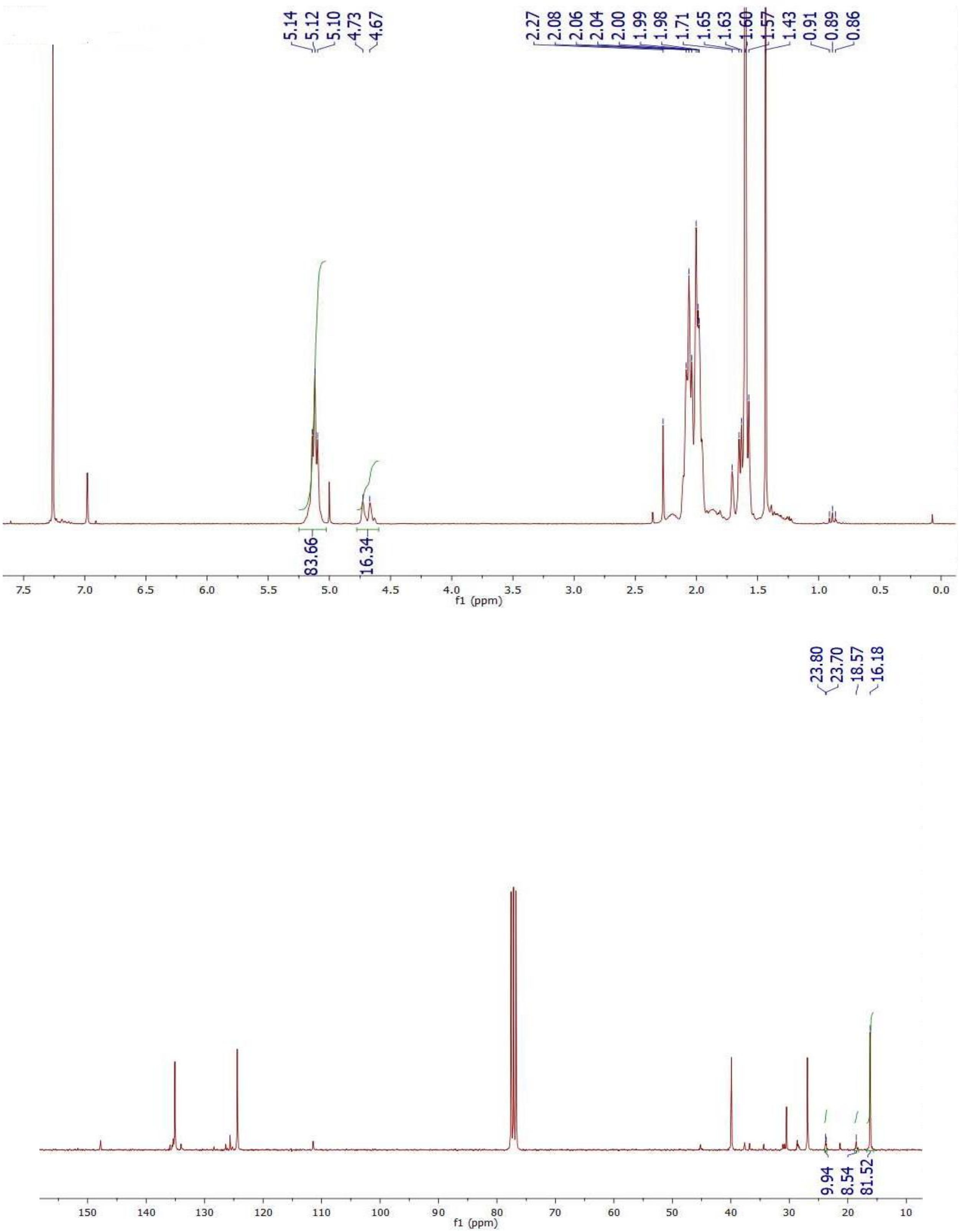

Figure S24. ^{1}H (top) and ^{13}C (bottom) NMR spectra of the polymer obtained with the **Entry 2** of **Table S1**.

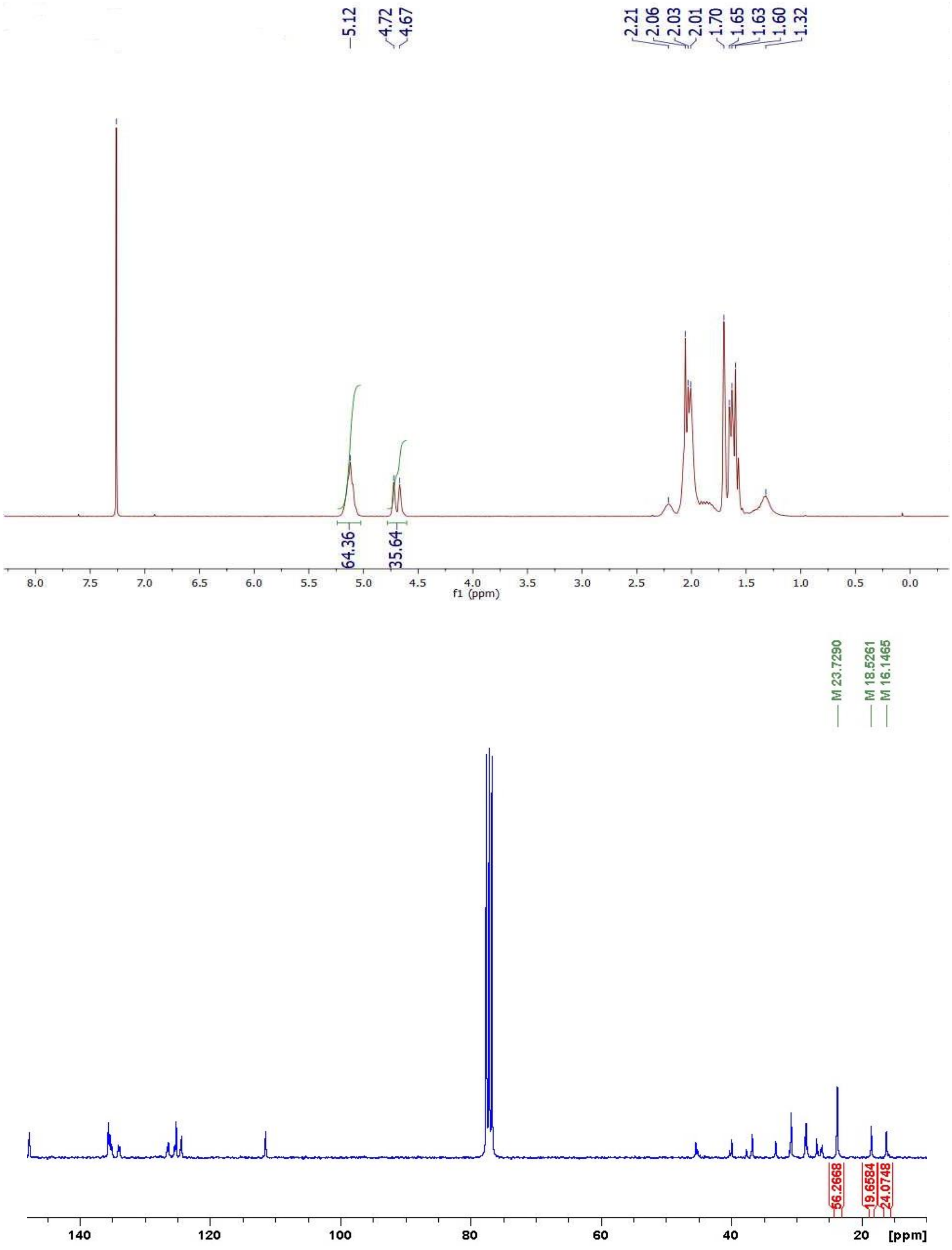

Figure S25. ^{1}H (top) and ^{13}C (bottom) NMR spectra of the polymer obtained with the **Entry 3** of **Table S1**.

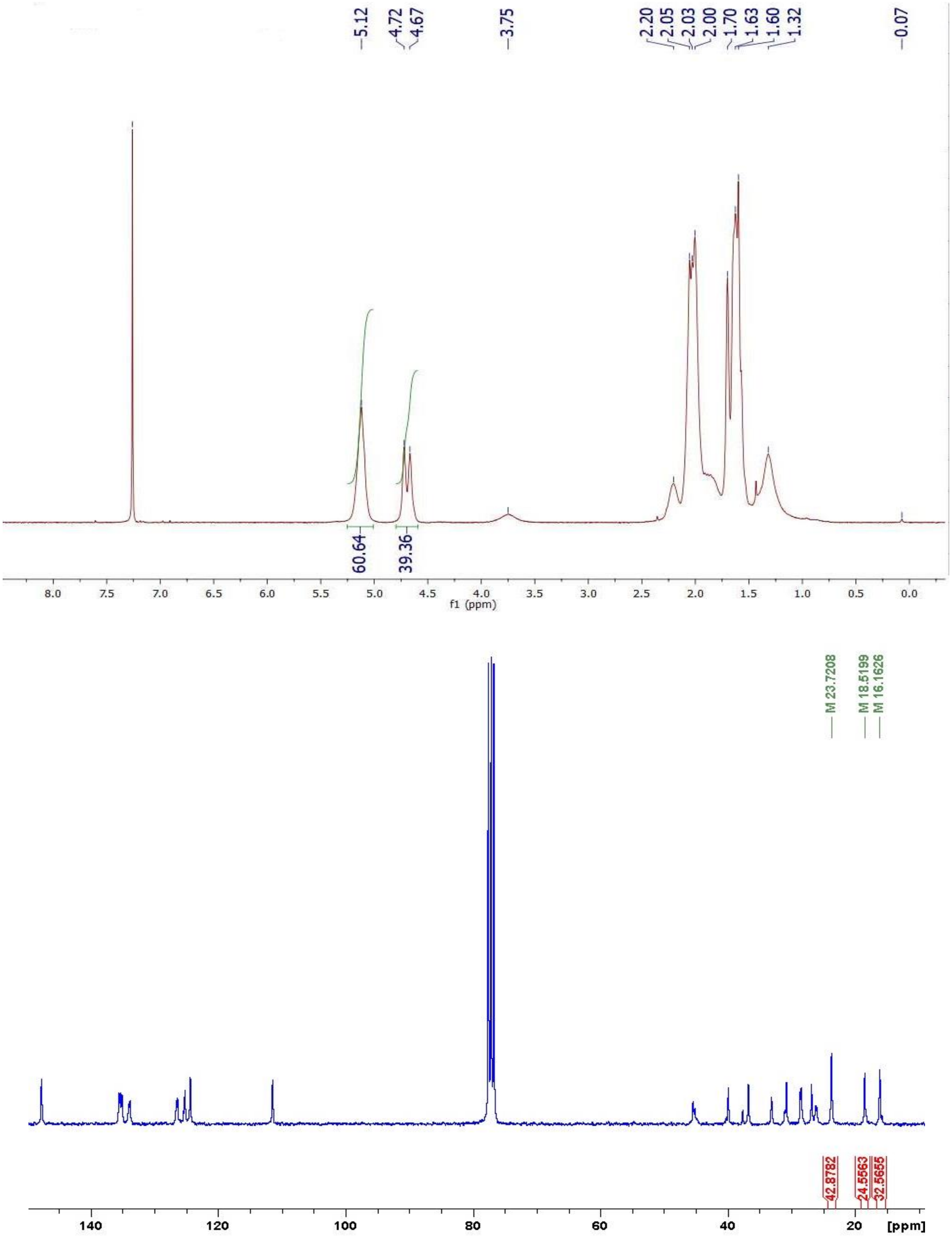

Figure S26. ^{1}H (top) and ^{13}C (bottom) NMR spectra of the polymer obtained with the **Entry 4** of **Table S1**.

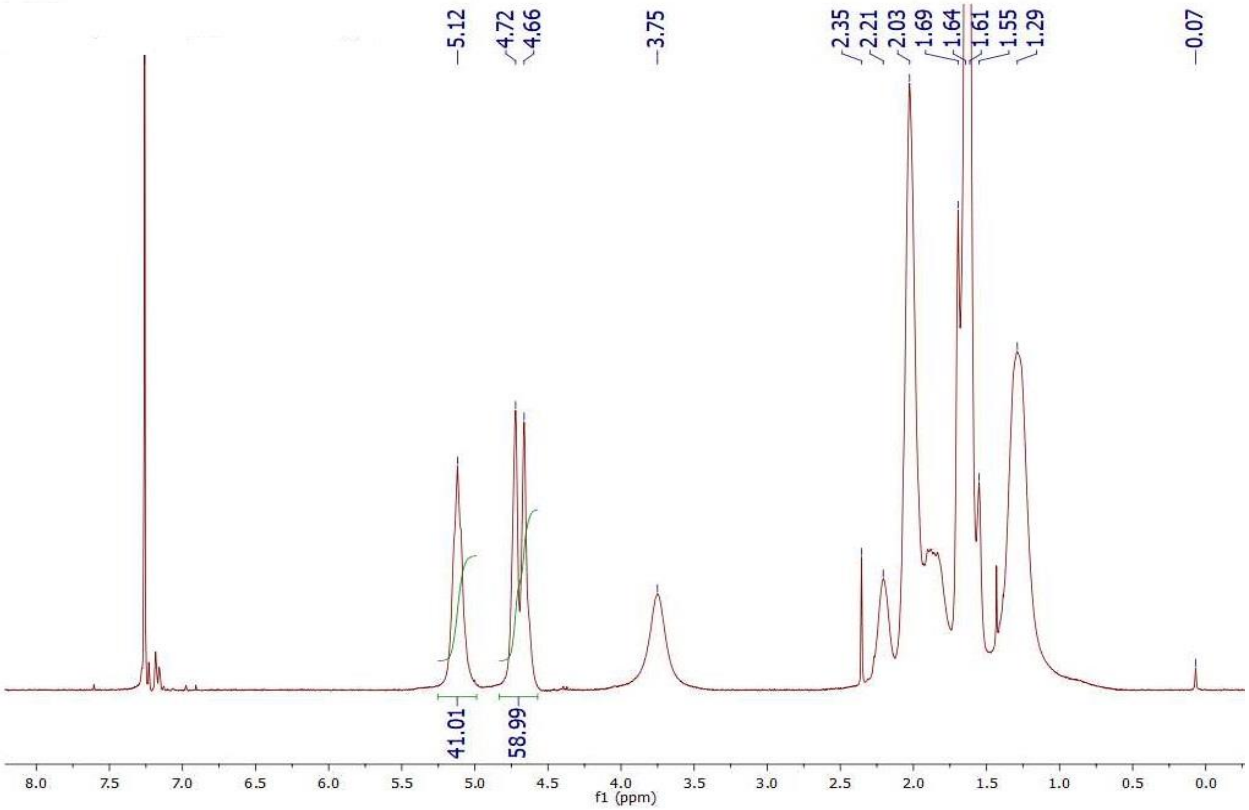

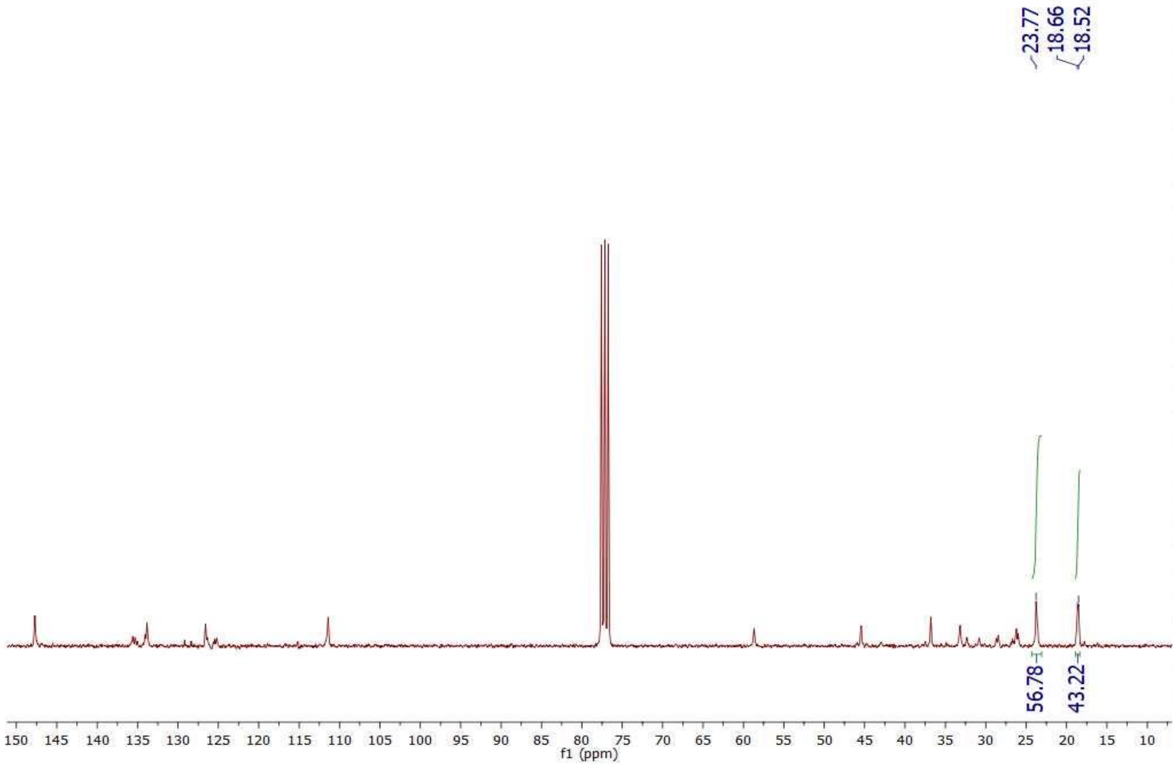

Figure S27. ^{1}H (top) and ^{13}C (bottom) NMR spectra of the polymer obtained with the **Entry 5** of **Table S1**.

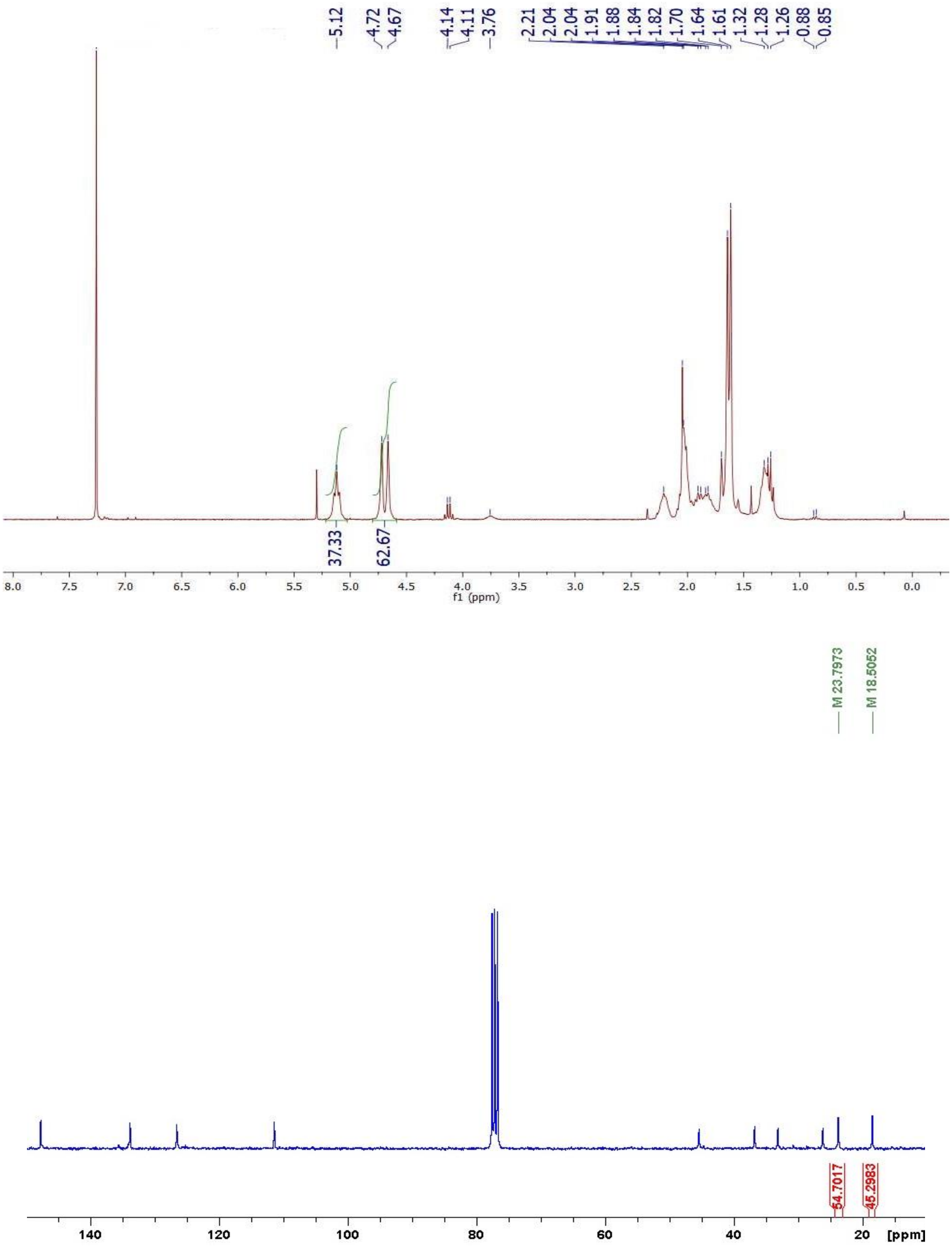

Figure S28. ^{1}H (top) and ^{13}C (bottom) NMR spectra of the polymer obtained with the **Entry 6** of **Table S1**.

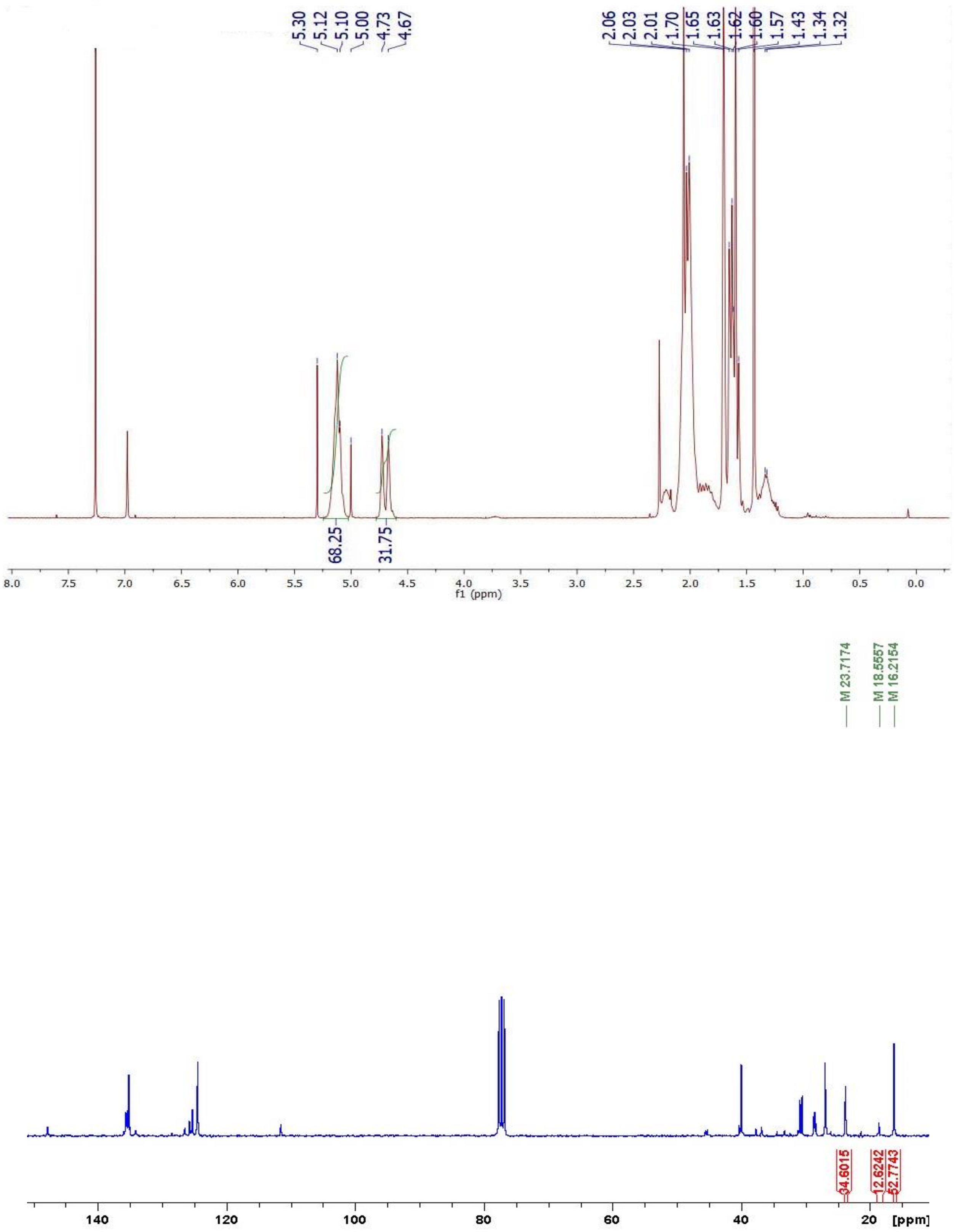

Figure S29. ¹H (top) and ¹³C (bottom) NMR spectra of the polymer obtained with the **Entry 1** of **Table S2**.

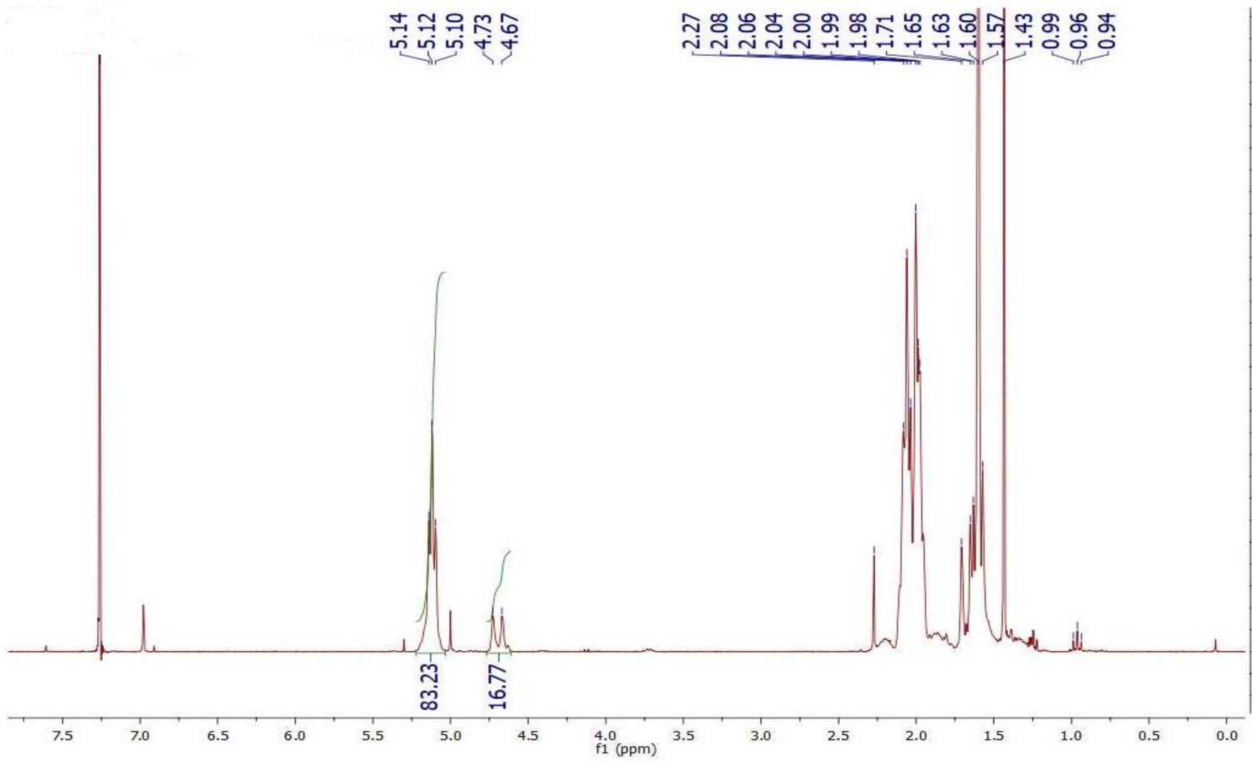

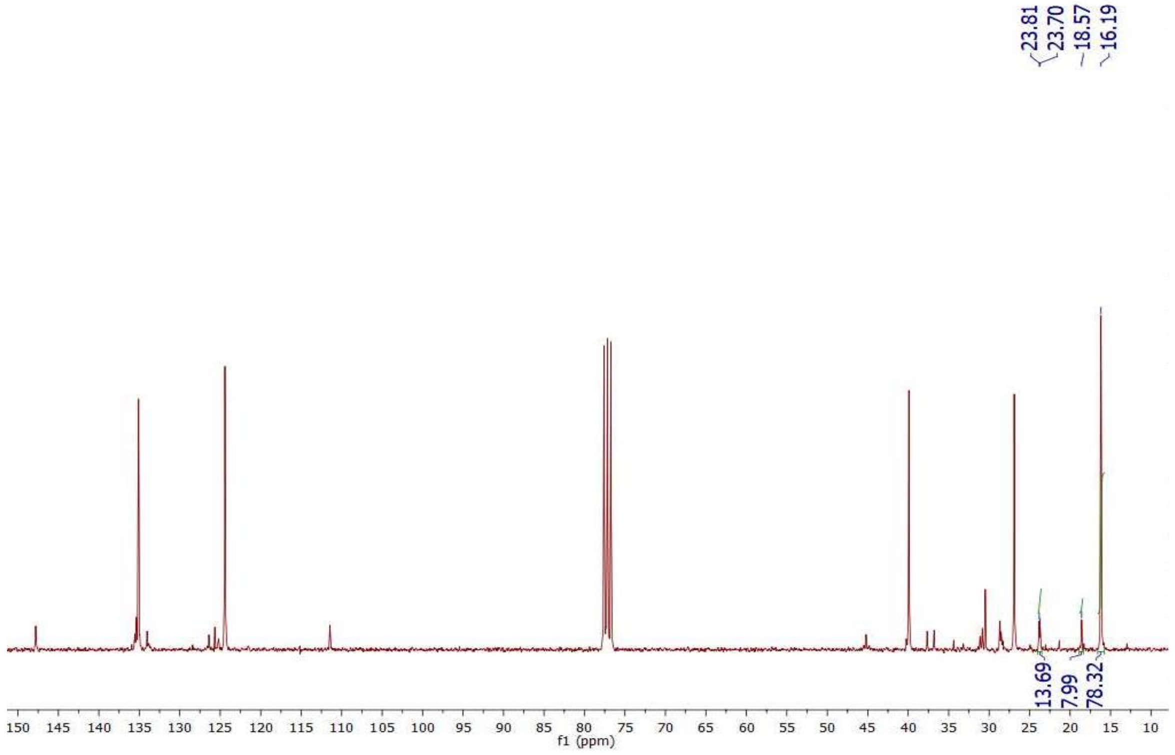

Figure S30. ^{1}H (top) and ^{13}C (bottom) NMR spectra of the polymer obtained with the **Entry 2** of **Table S2**.

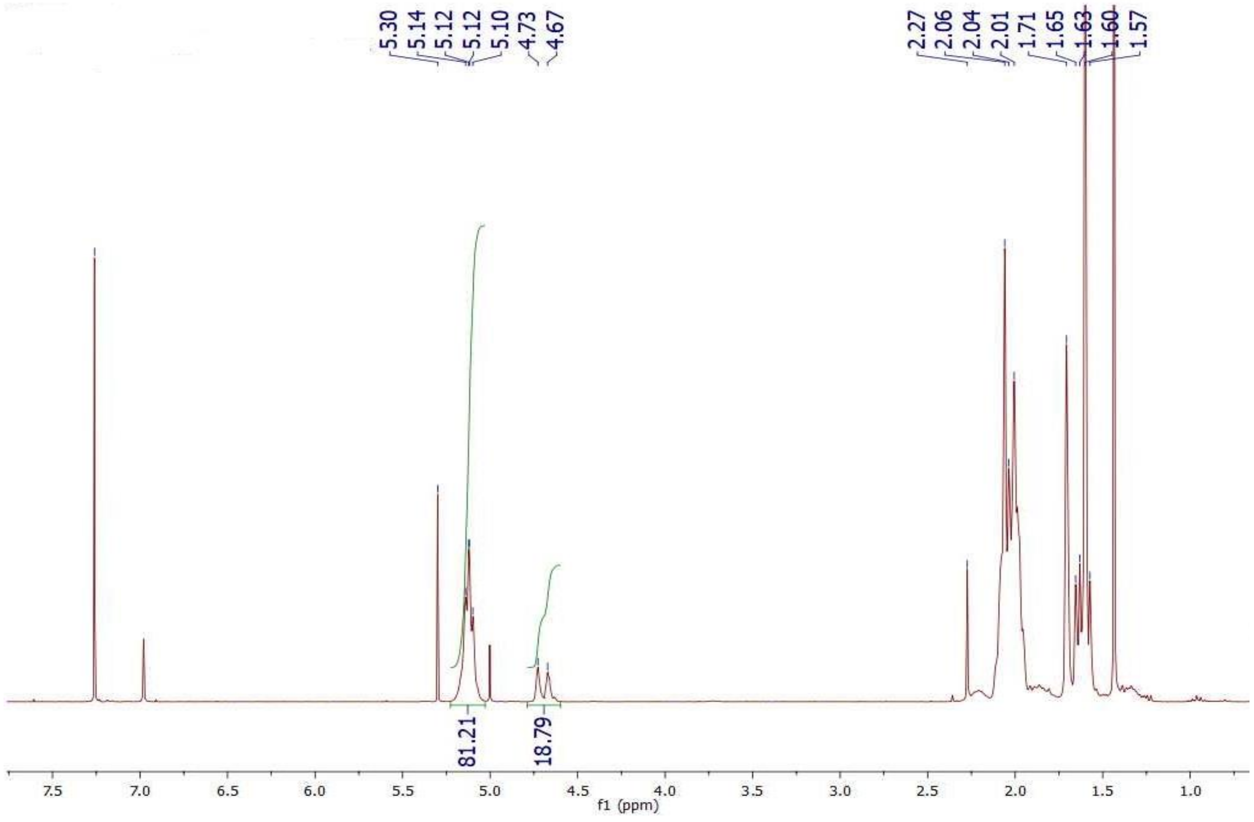

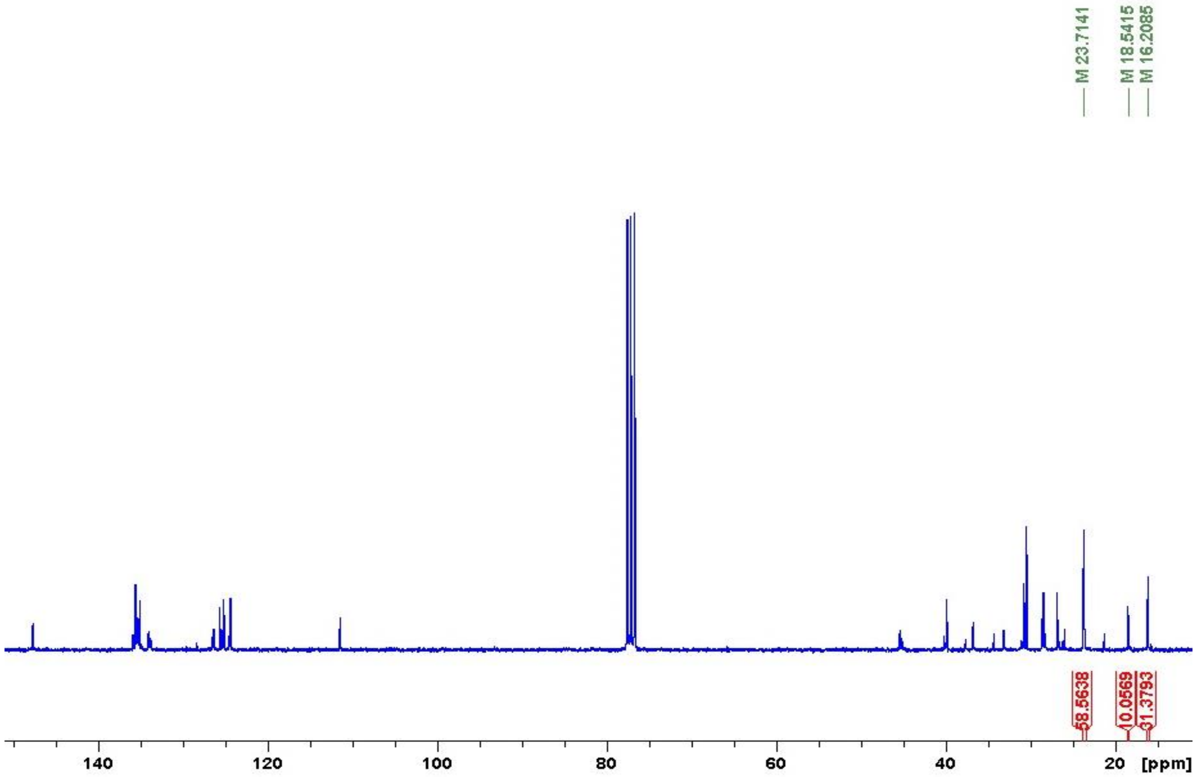

Figure S31. ^{1}H (top) and ^{13}C (bottom) NMR spectra of the polymer obtained with the **Entry 3** of **Table S2**.

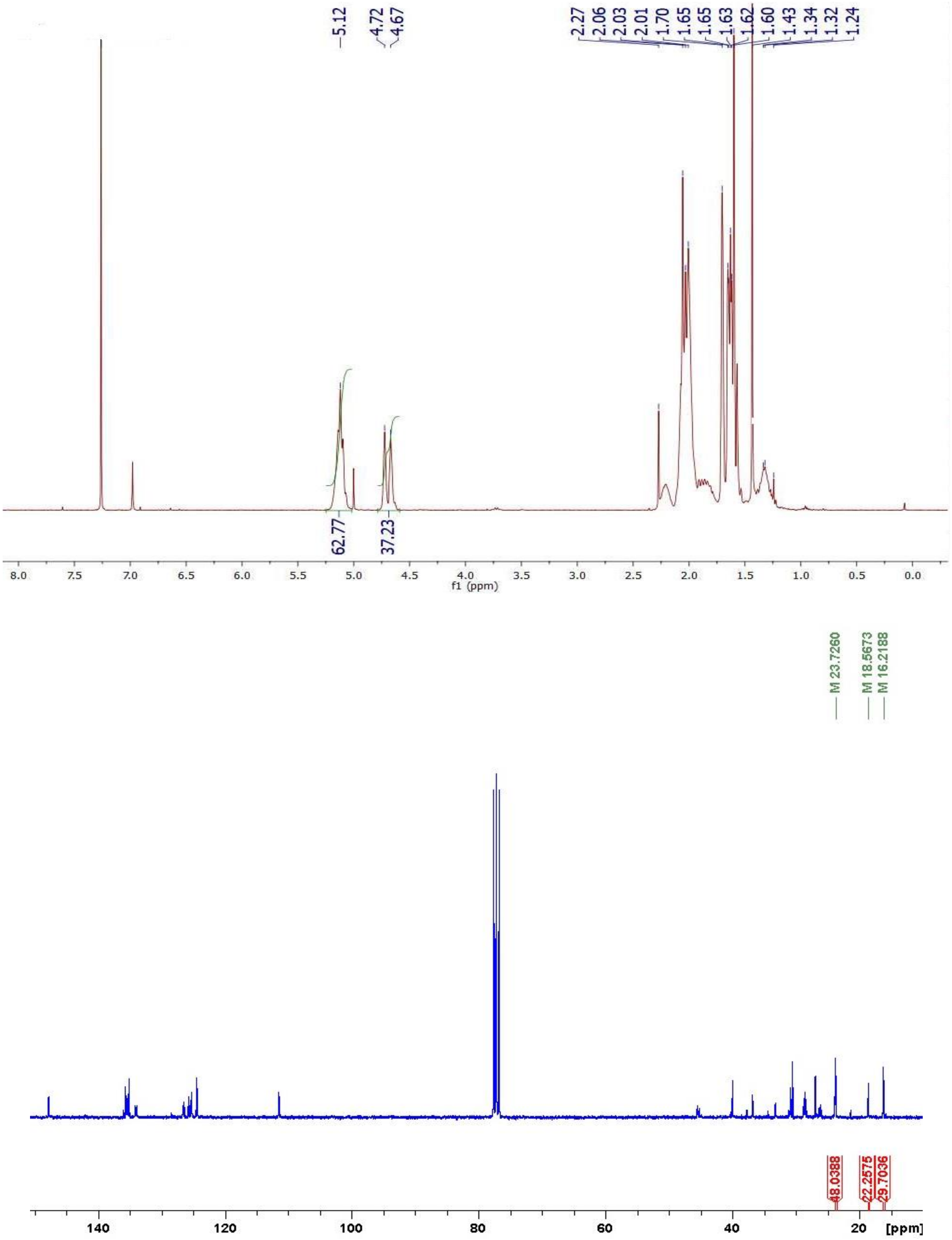

Figure S32. ^{1}H (top) and ^{13}C (bottom) NMR spectra of the polymer obtained with the **Entry 4** of **Table S2**.

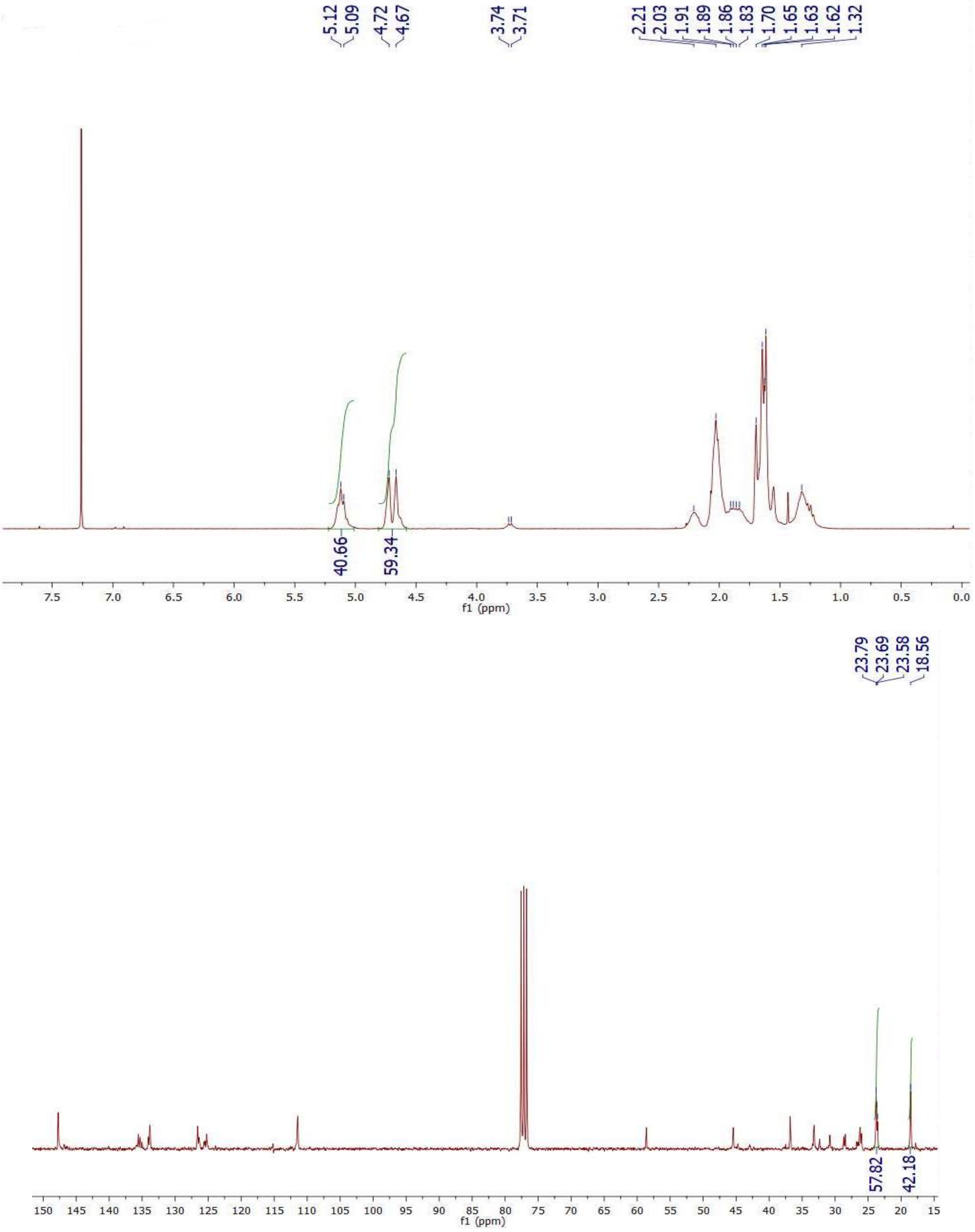

Figure S33. ^{1}H (top) and ^{13}C (bottom) NMR spectra of the polymer obtained with the **Entry 5** of **Table S2**.

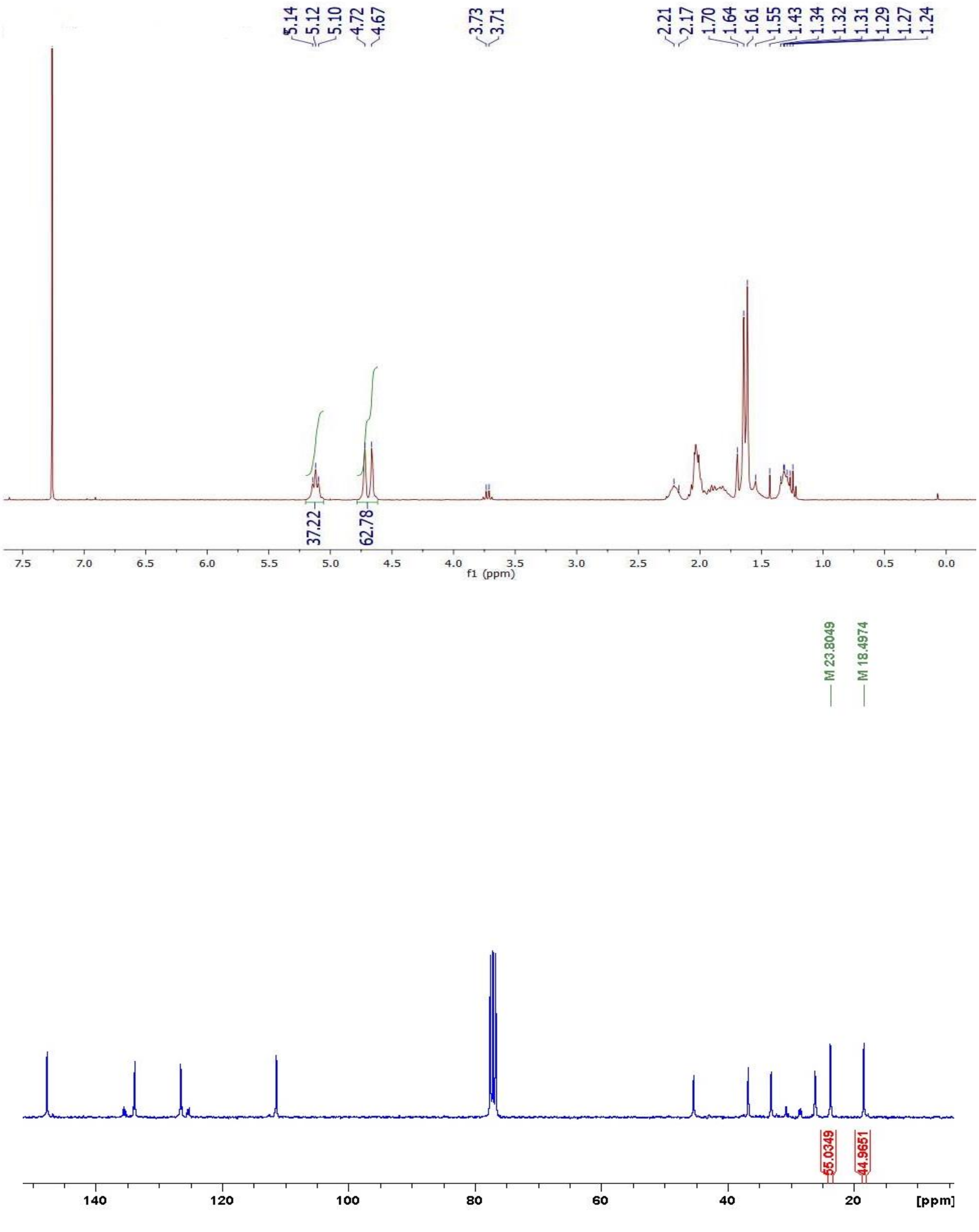

Figure S34. ^{1}H (top) and ^{13}C (bottom) NMR spectra of the polymer obtained with the **Entry 6** of **Table S2**.

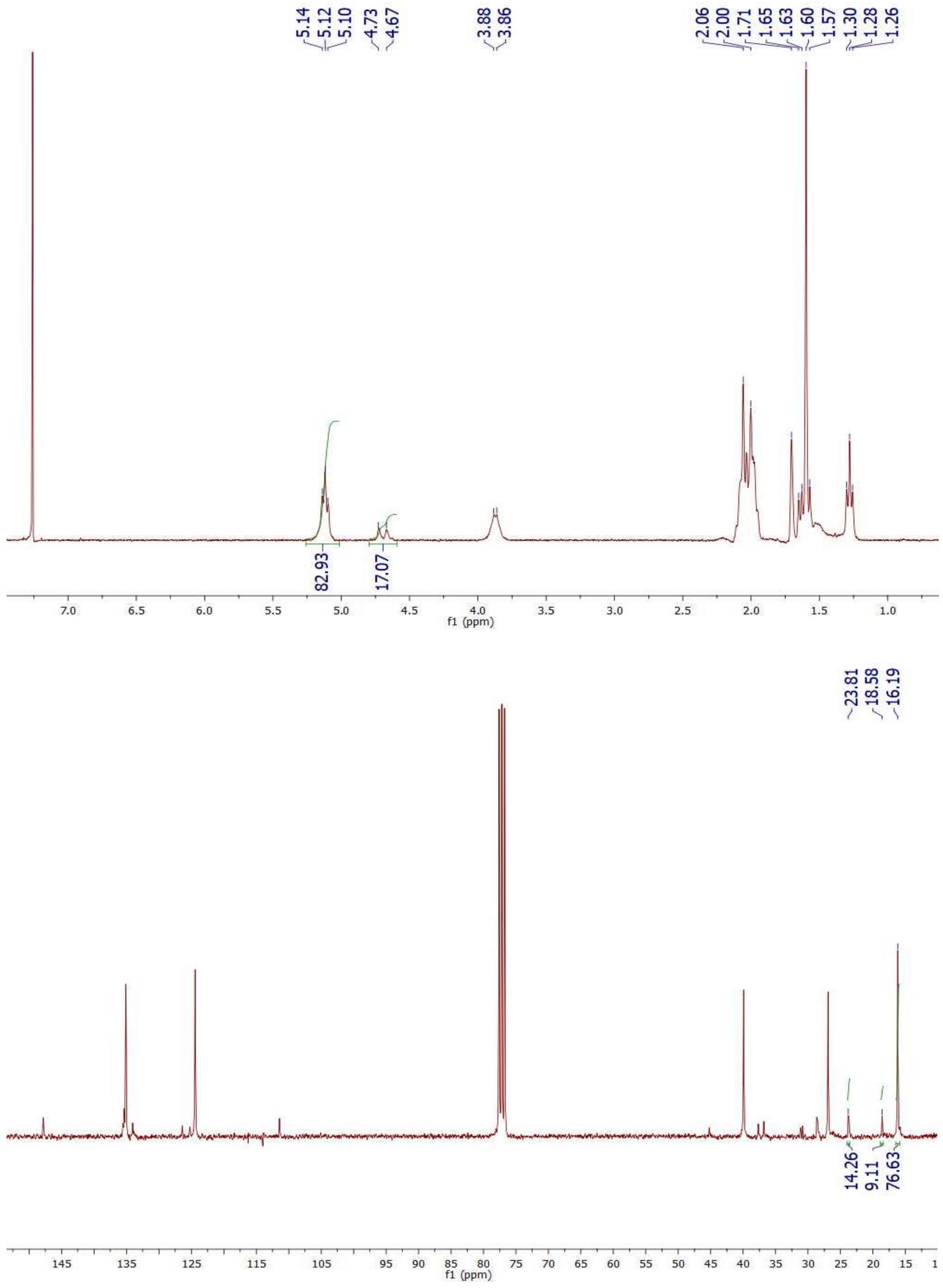

Figure S35. ^{1}H (top) and ^{13}C (bottom) NMR spectra of the polymer obtained with the **Entry 1** of **Table S3**.

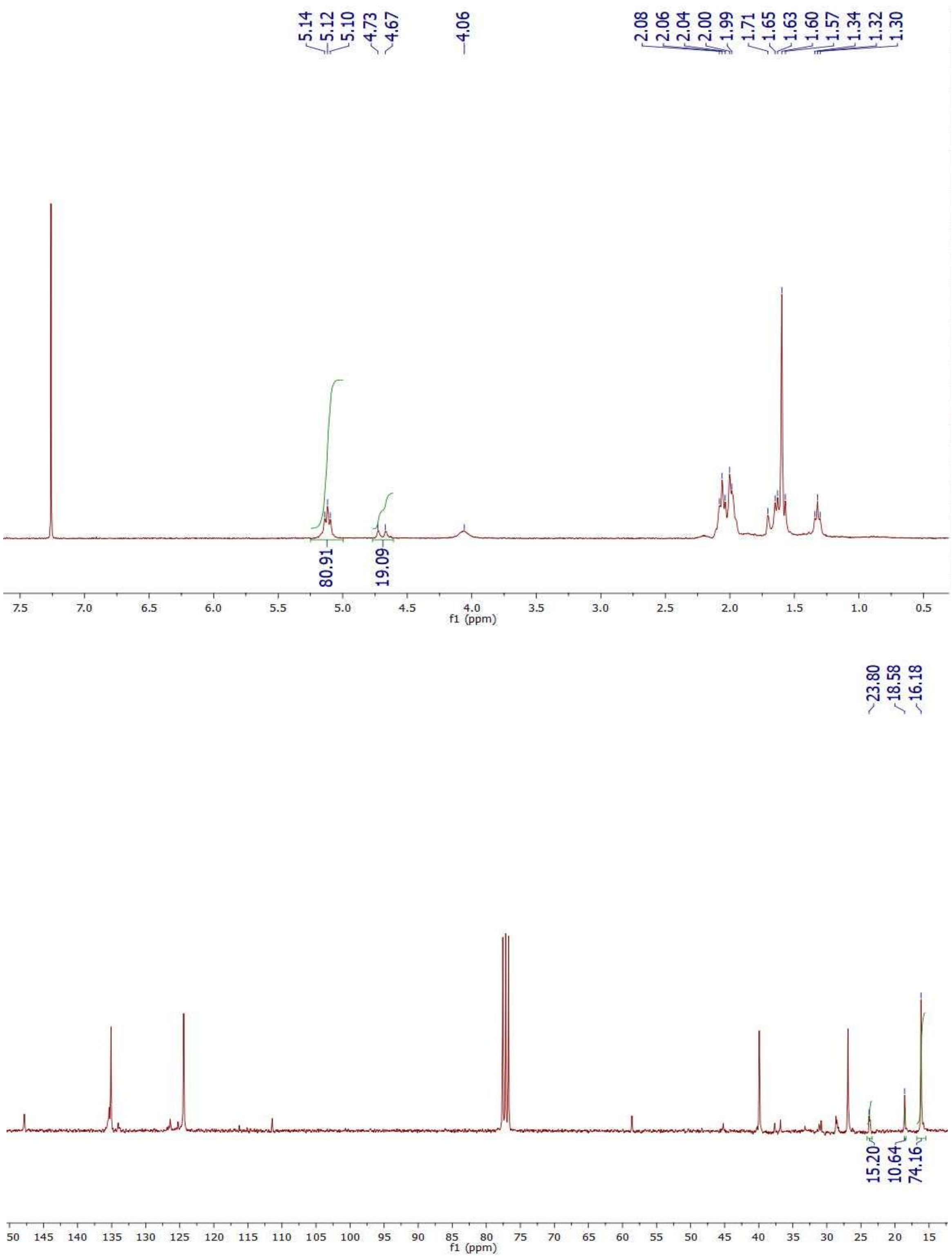

Figure S36. ^{1}H (top) and ^{13}C (bottom) NMR spectra of the polymer obtained with the **Entry 2** of **Table S3**.

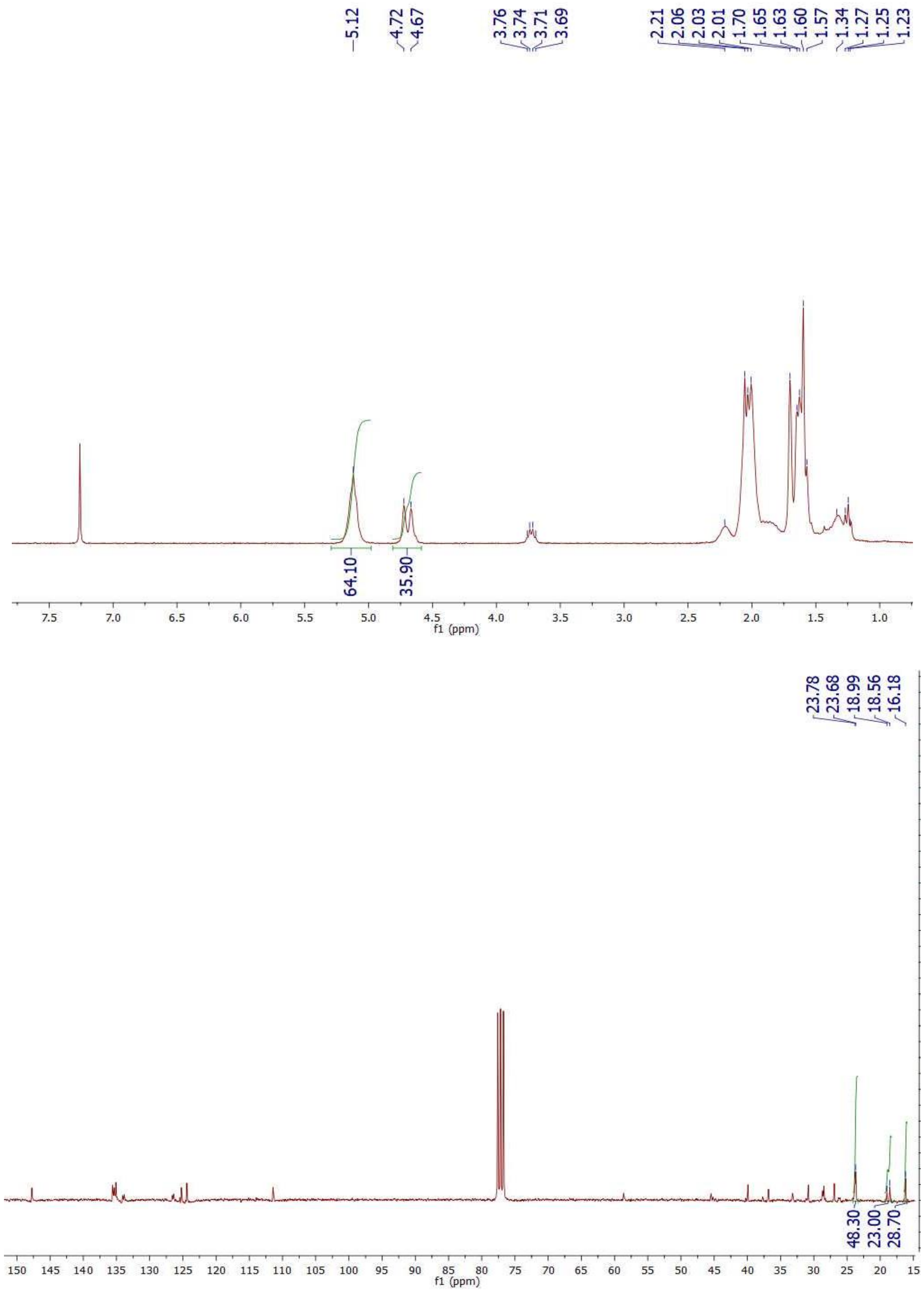

Figure S37. ^{1}H (top) and ^{13}C (bottom) NMR spectra of the polymer obtained with the **Entry 3** of **Table S3**.

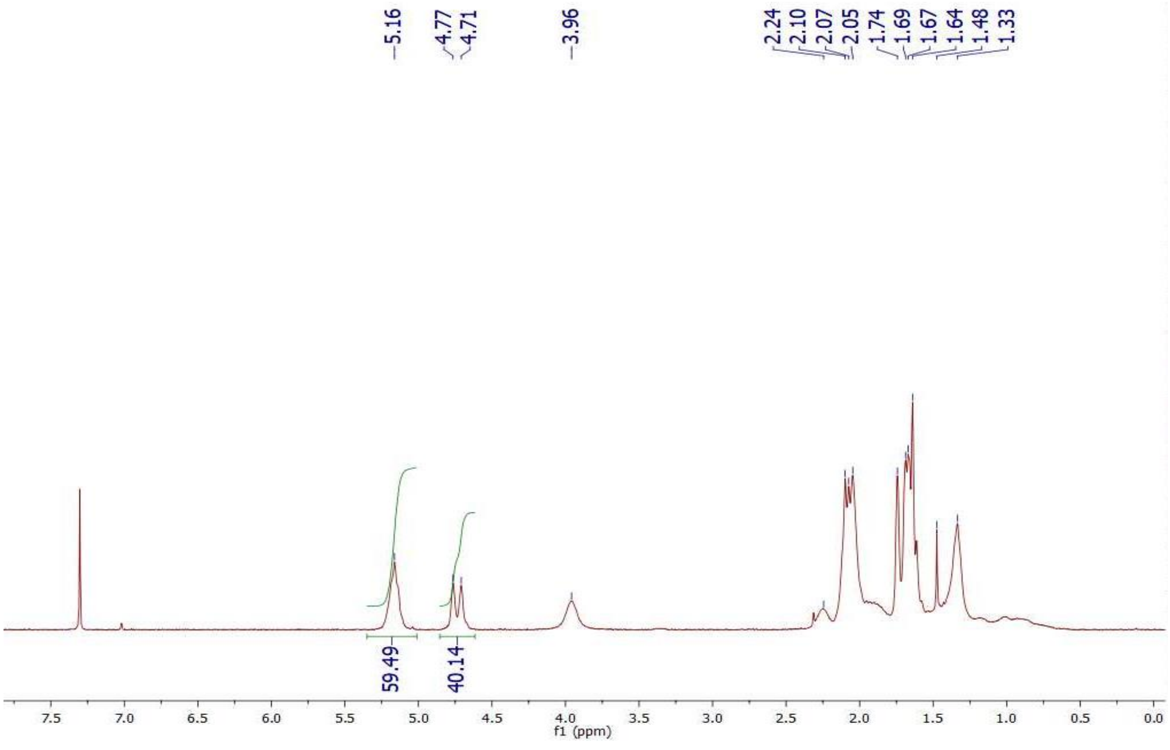

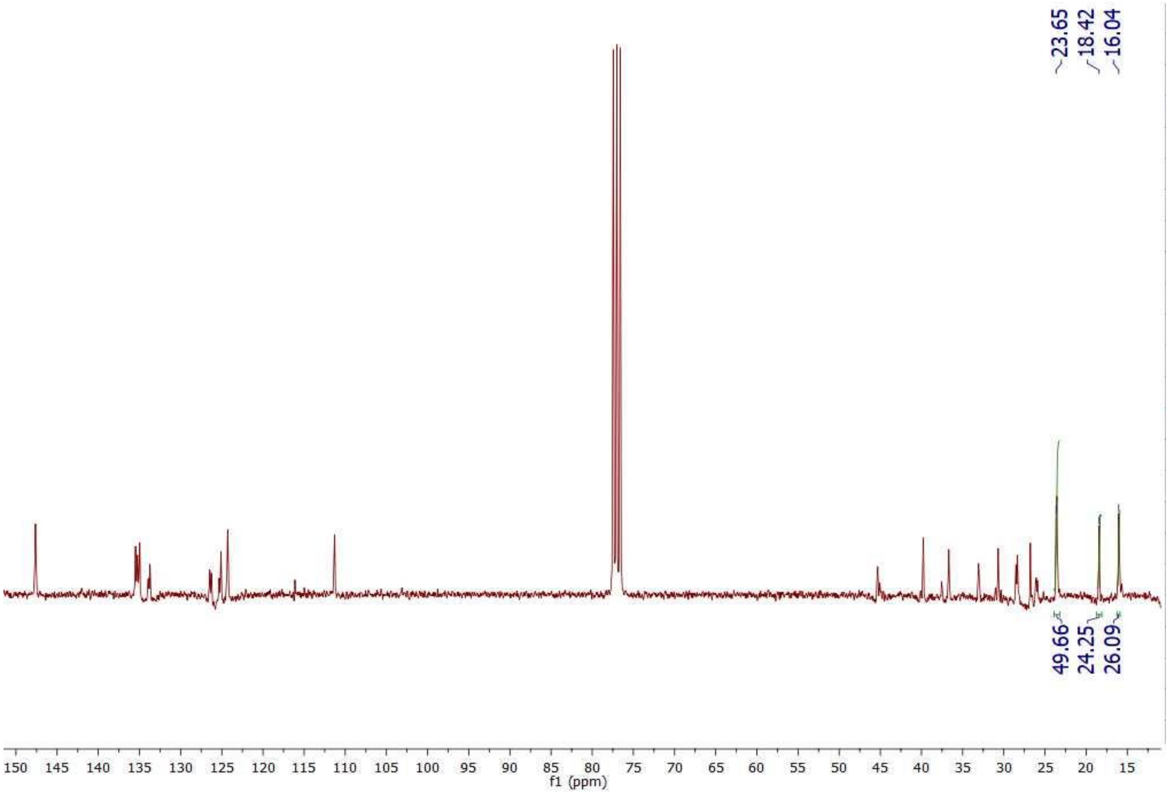

Figure S38. ^{1}H (top) and ^{13}C (bottom) NMR spectra of the polymer obtained with the **Entry 4** of **Table S3**.

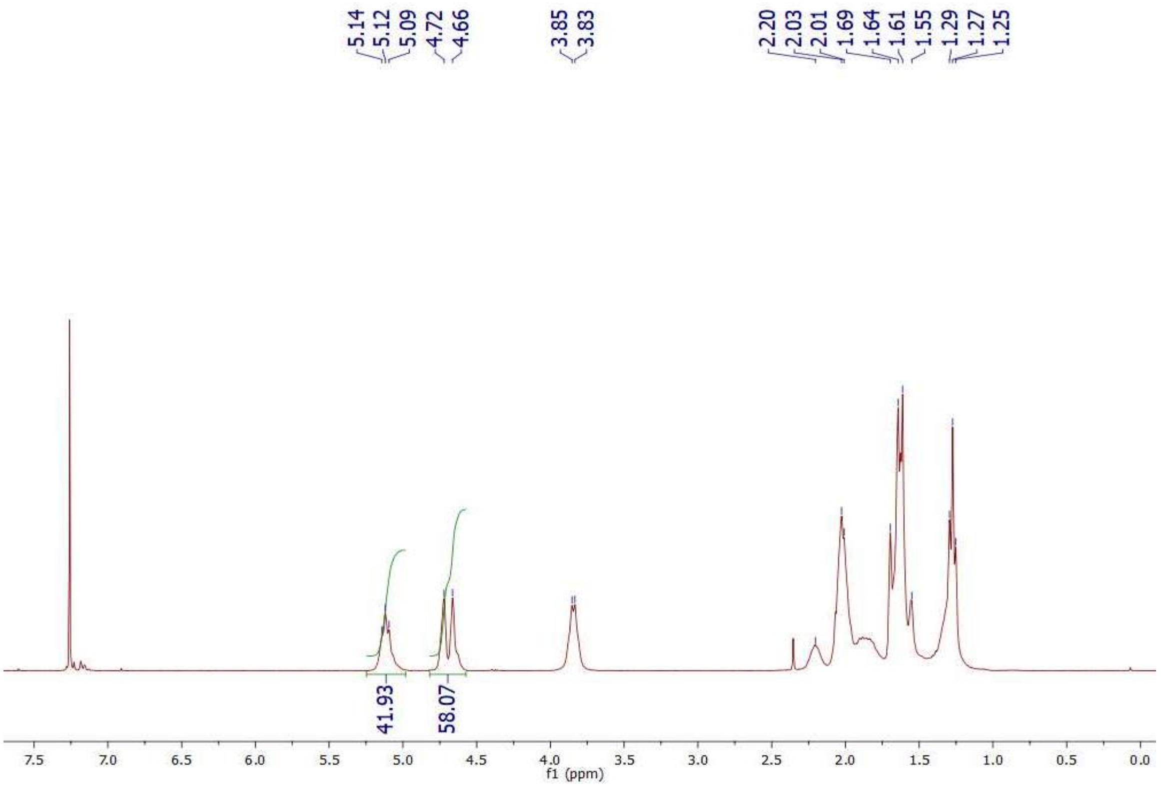

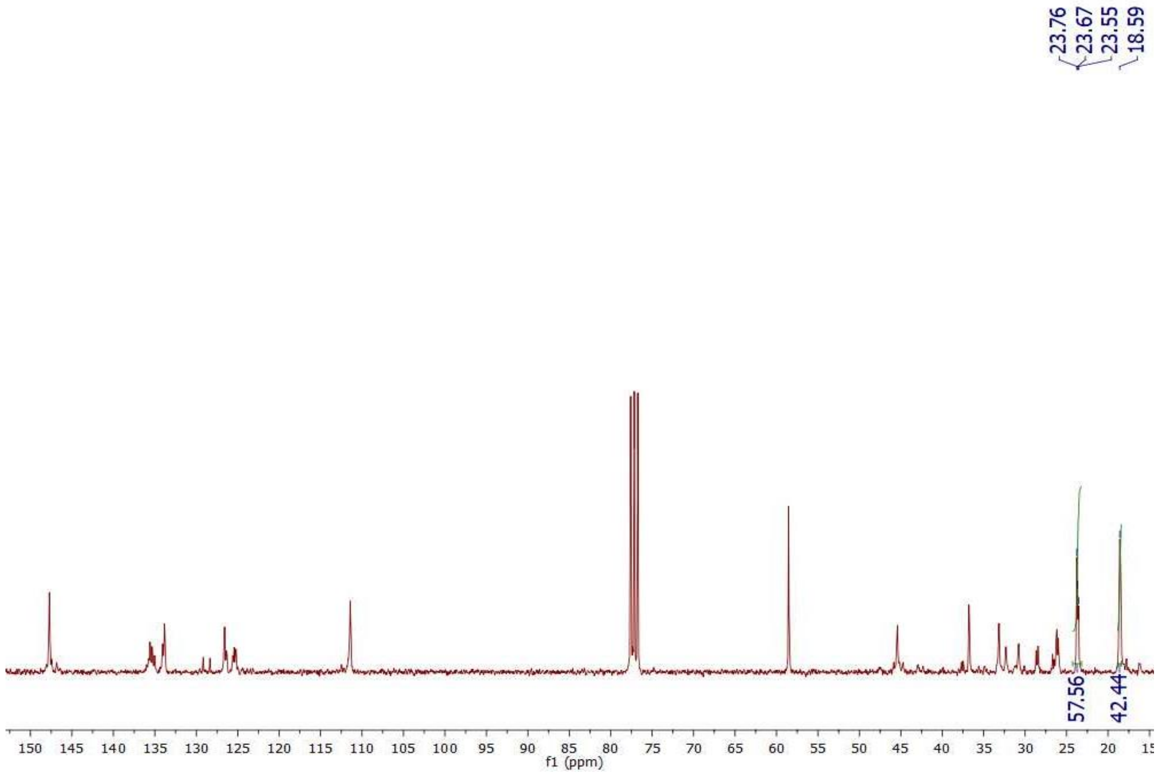

Figure S39. ^{1}H (top) and ^{13}C (bottom) NMR spectra of the polymer obtained with the **Entry 5** of **Table S3**.

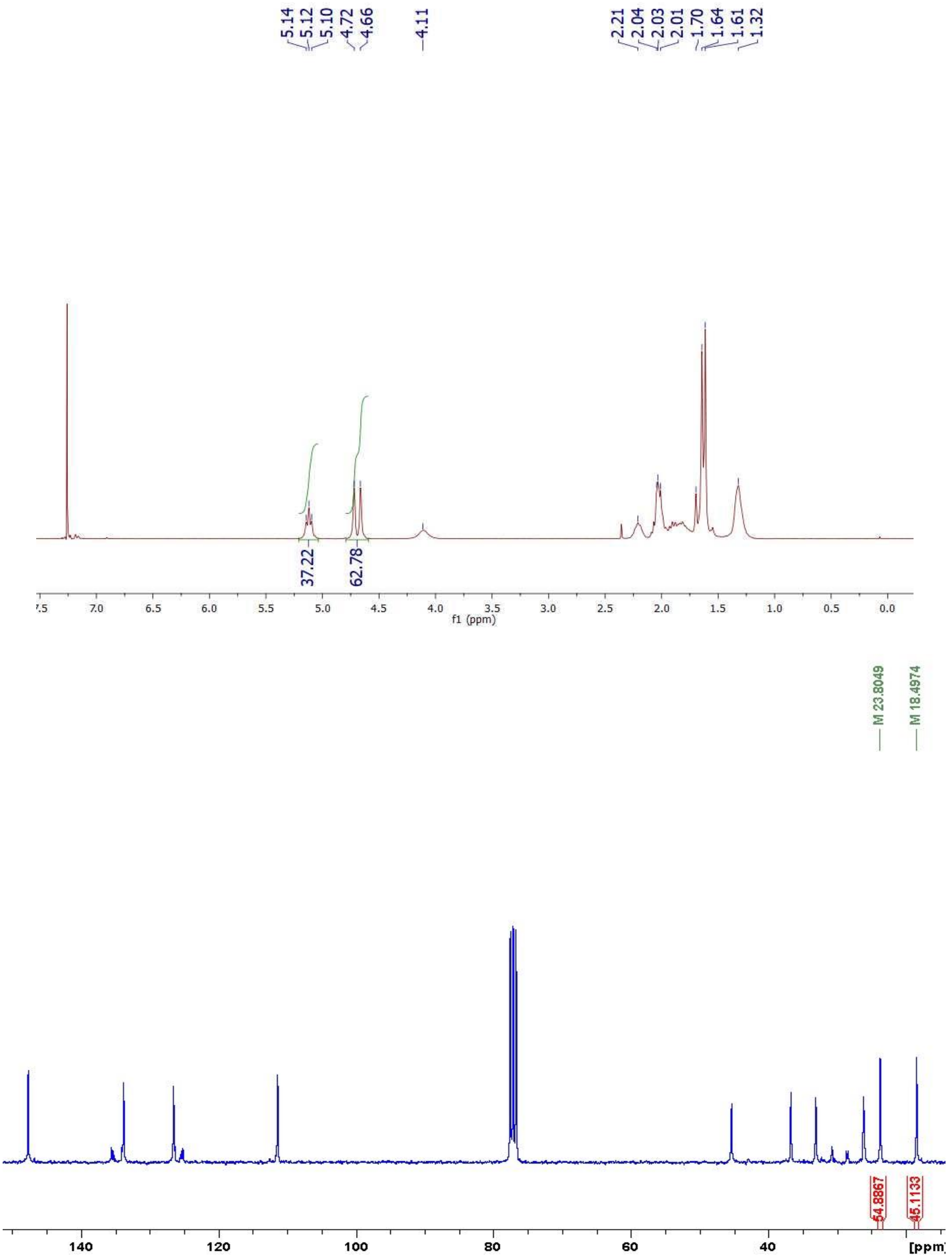

Figure S40. ^1H (top) and ^{13}C (bottom) NMR spectra of the polymer obtained with the **Entry 6** of **Table S3**.

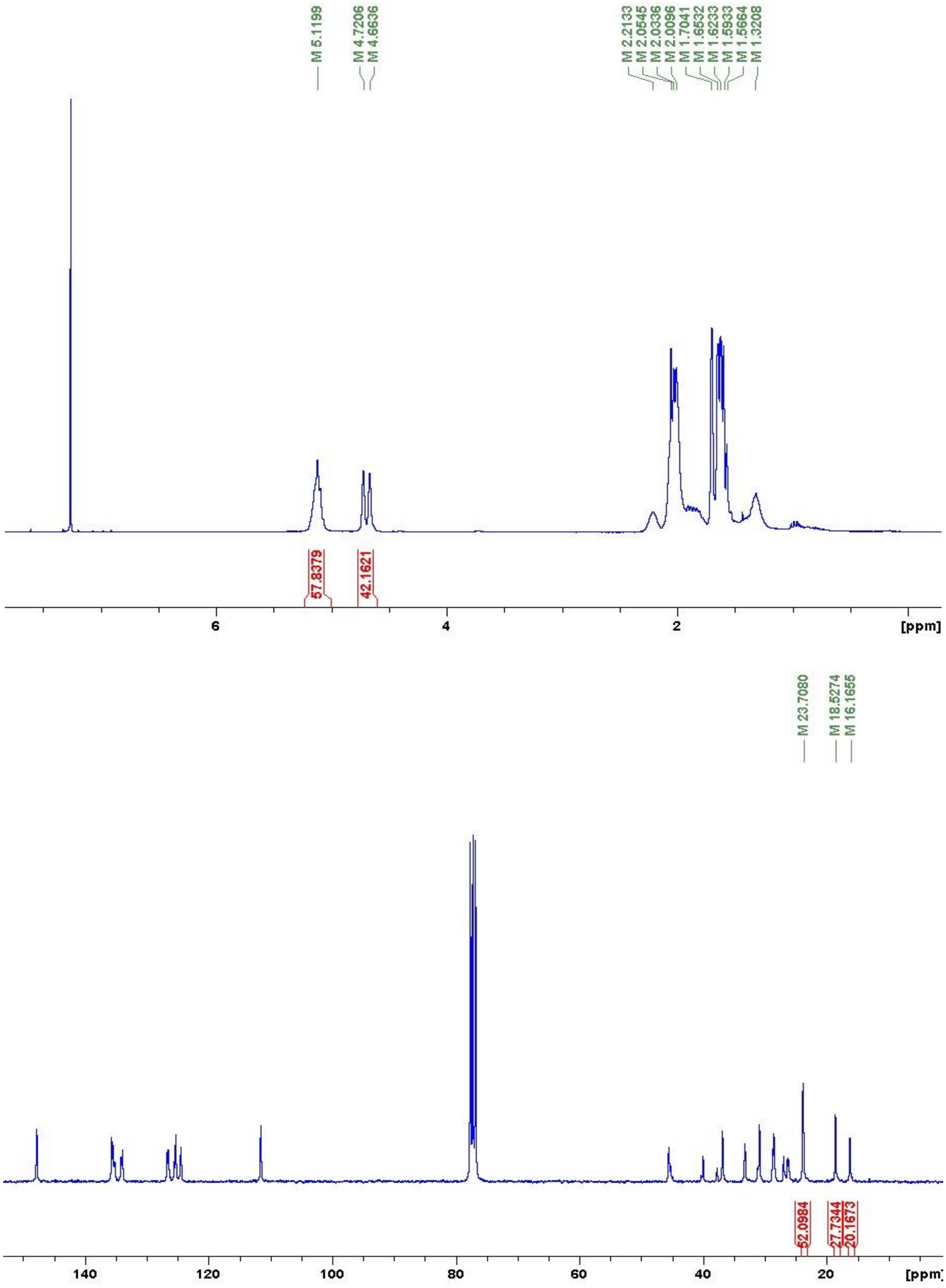

Figure S41. ^{1}H (top) and ^{13}C (bottom) NMR spectra of the polymer obtained with the **Entry 1** of **Table S4**.

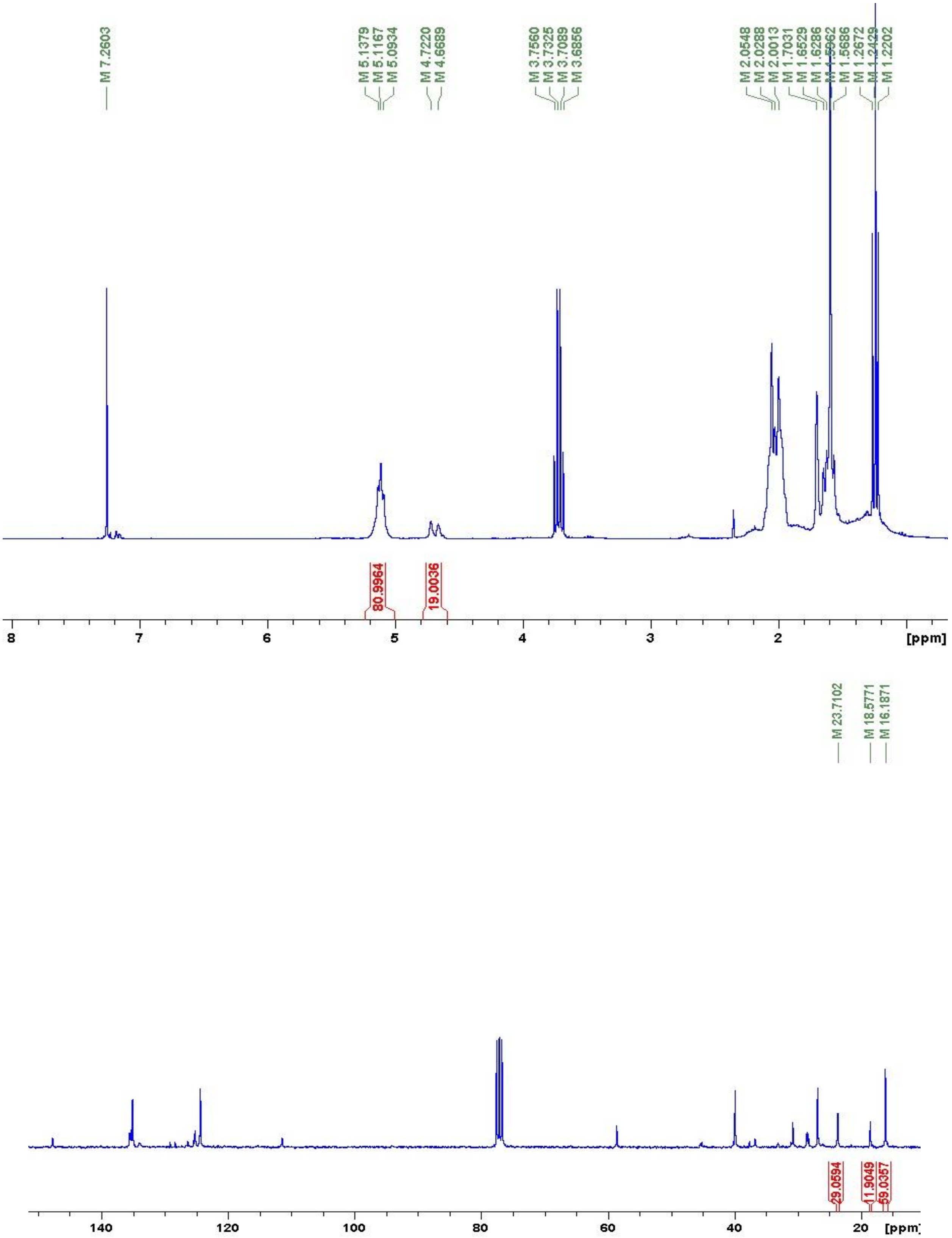

Figure S42. ^{1}H (top) and ^{13}C (bottom) NMR spectra of the polymer obtained with the **Entry 1** of **Table 2**.

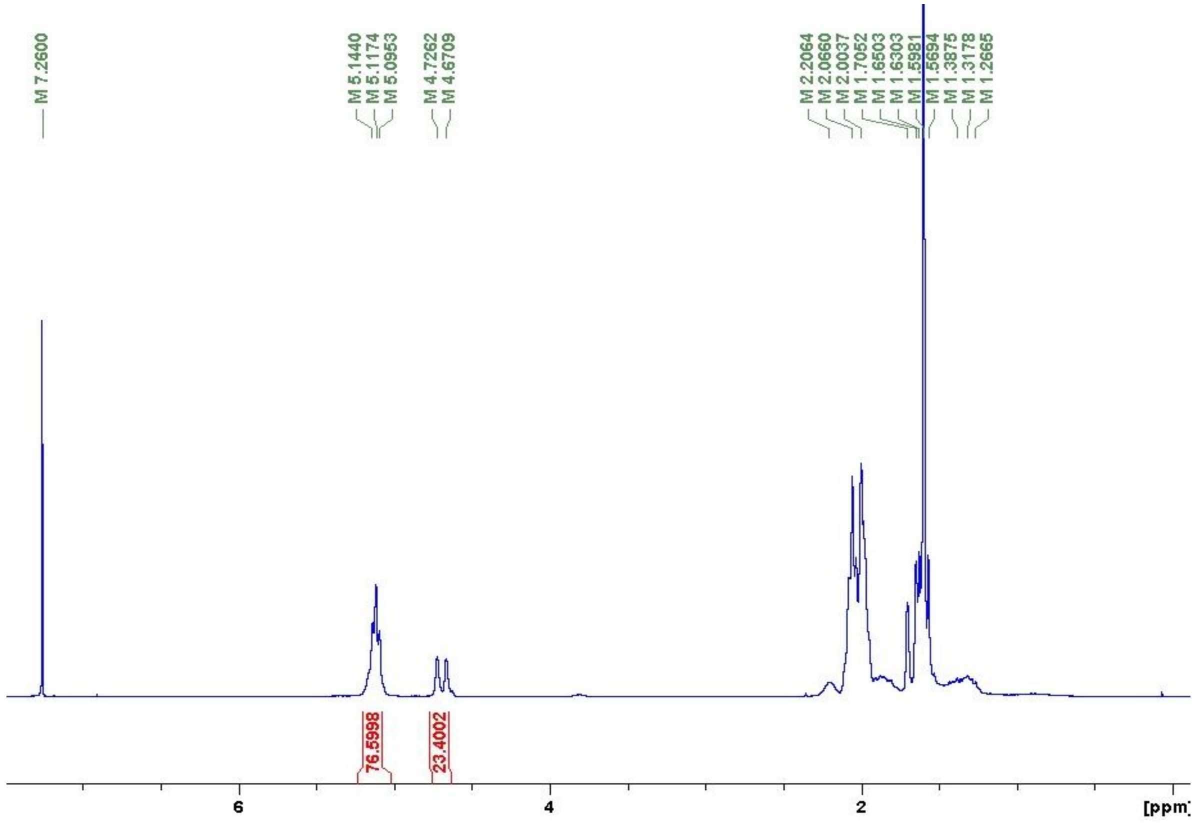

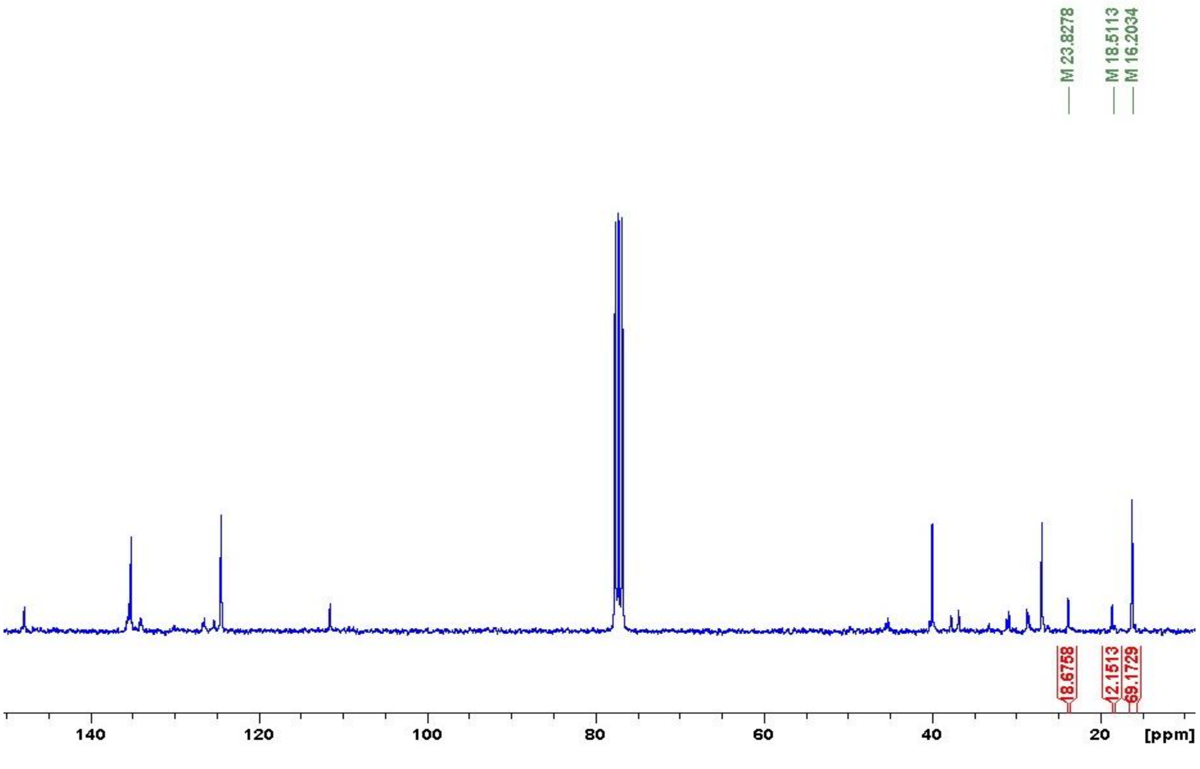

Figure S43. ^{1}H (top) and ^{13}C (bottom) NMR spectra of the polymer obtained with the **Entry 2** of **Table 2**.

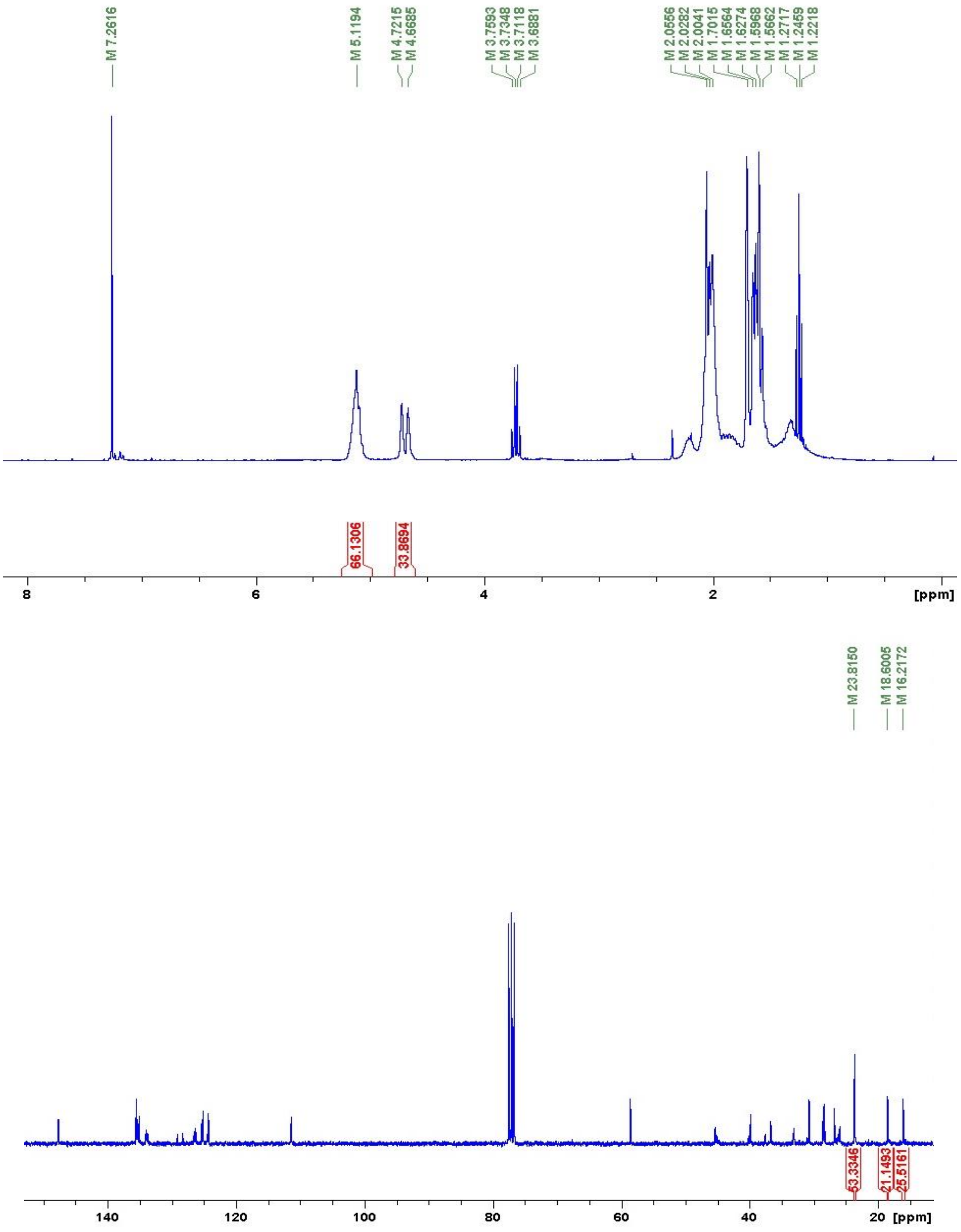

Figure S44. ^{1}H (top) and ^{13}C (bottom) NMR spectra of the polymer obtained with the **Entry 3** of **Table 2**.

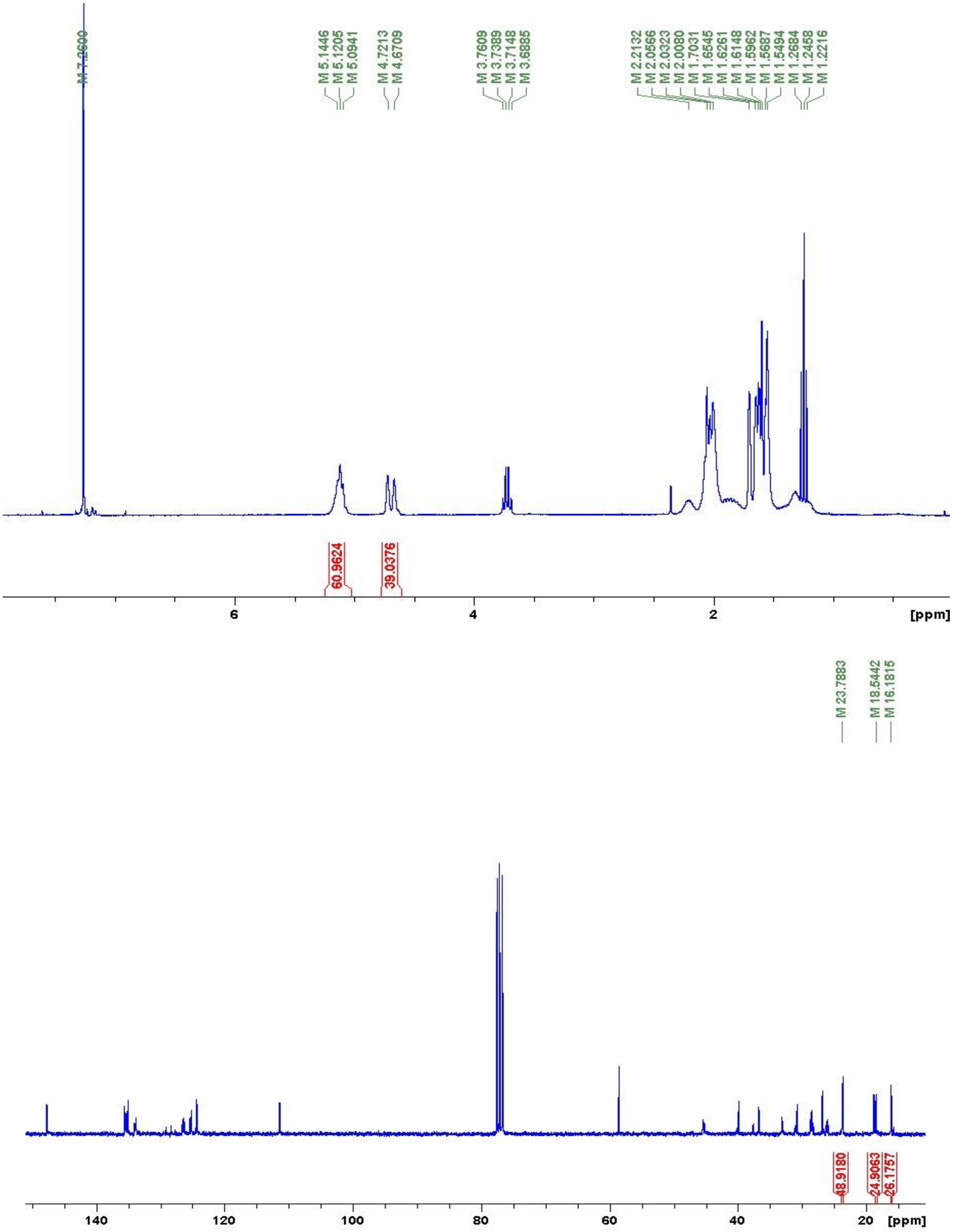

Figure S45. ^{1}H (top) and ^{13}C (bottom) NMR spectra of the polymer obtained with the **Entry 4** of **Table 2**.

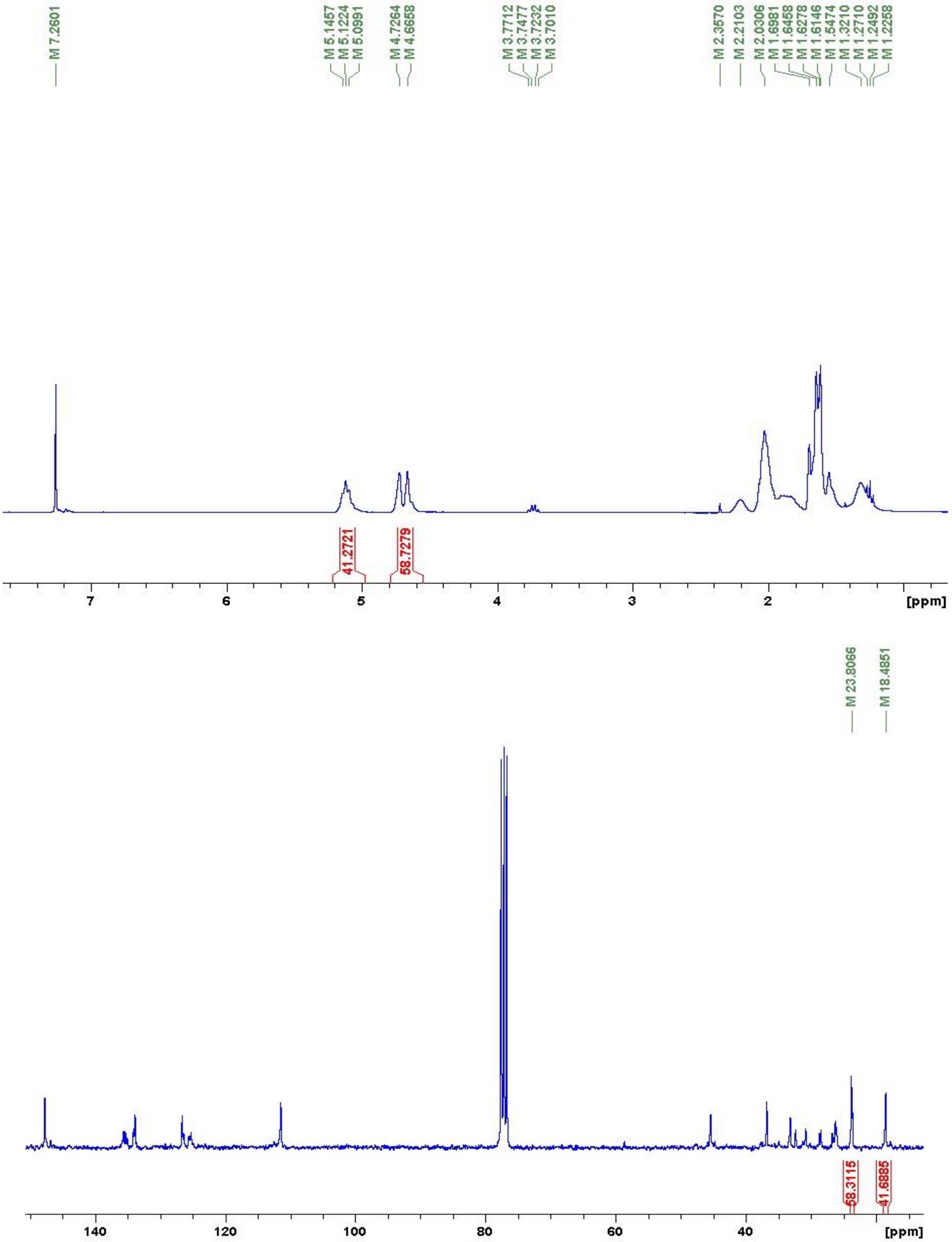

Figure S46. ^{1}H (top) and ^{13}C (bottom) NMR spectra of the polymer obtained with the **Entry 5** of **Table 2**.

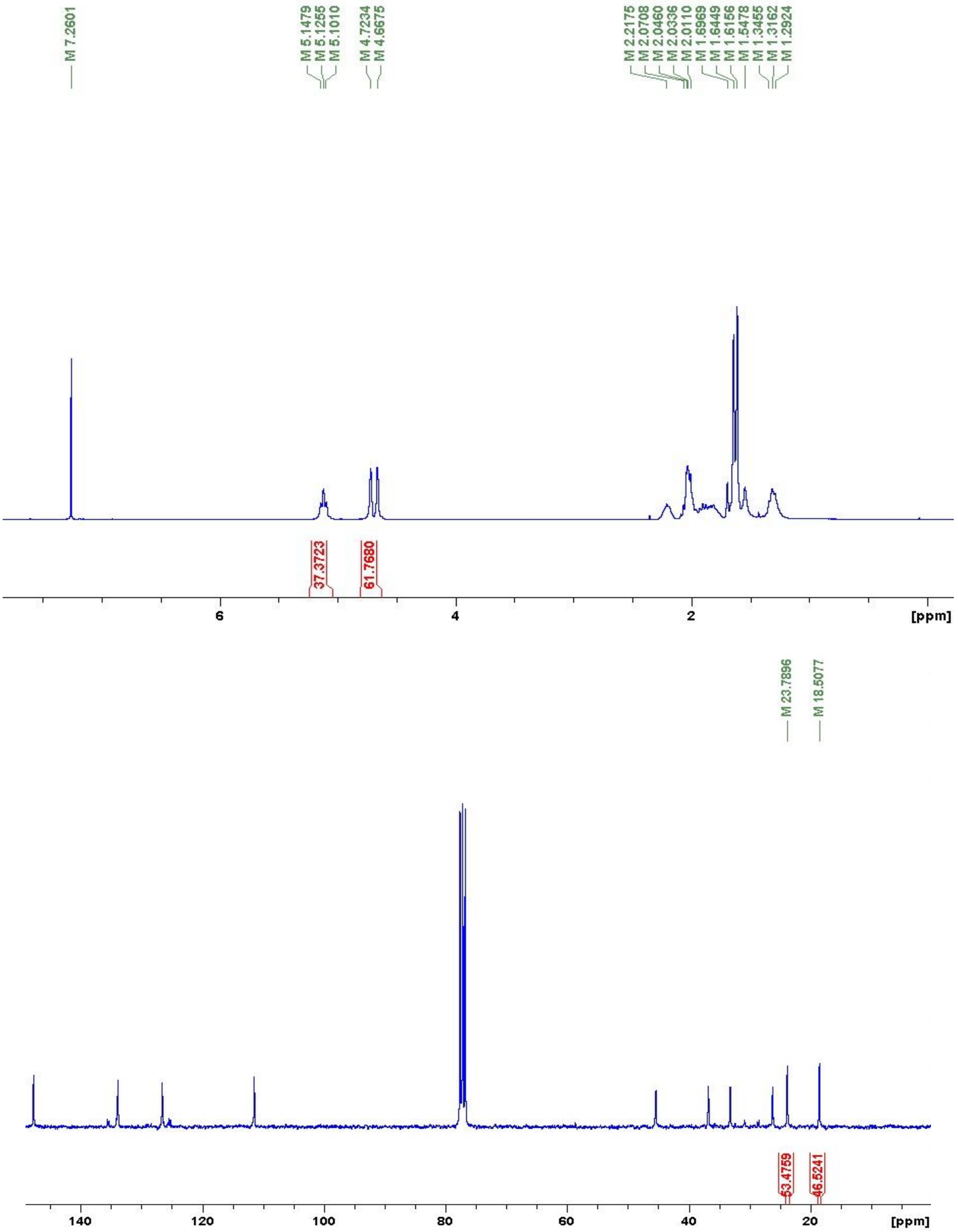

Figure S47. ^{1}H (top) and ^{13}C (bottom) NMR spectra of the polymer obtained with the **Entry 6** of **Table 2**.

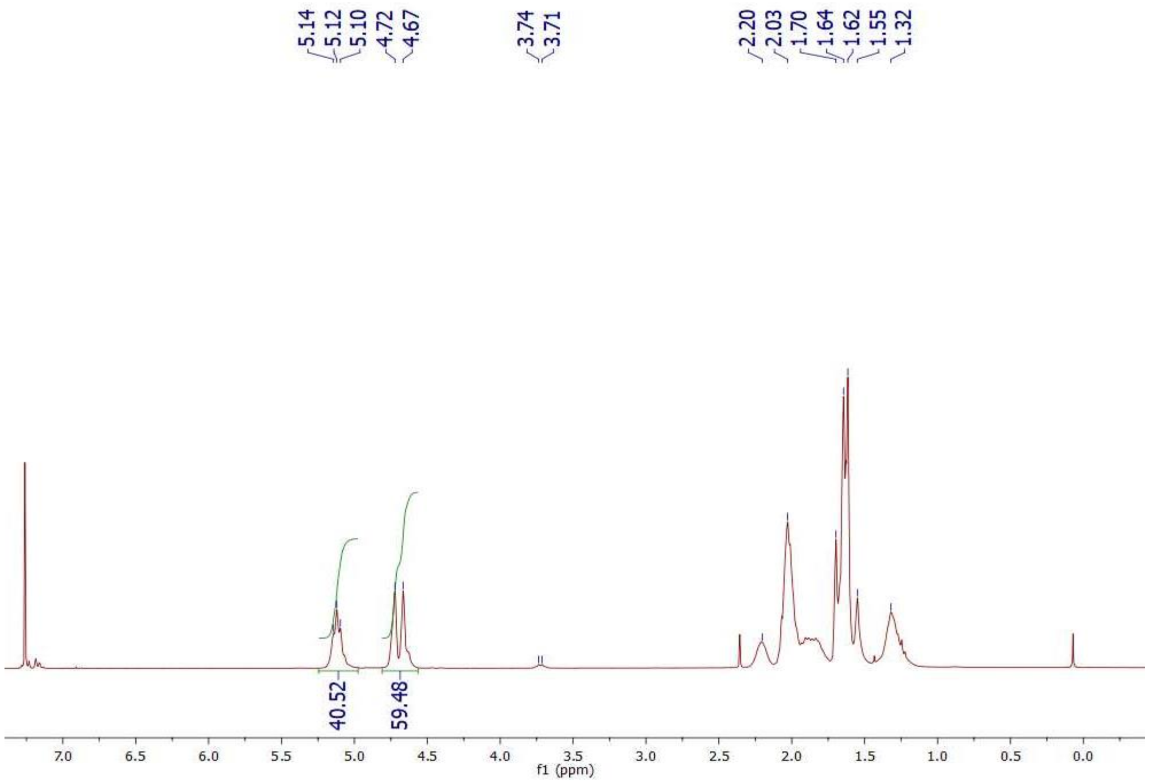

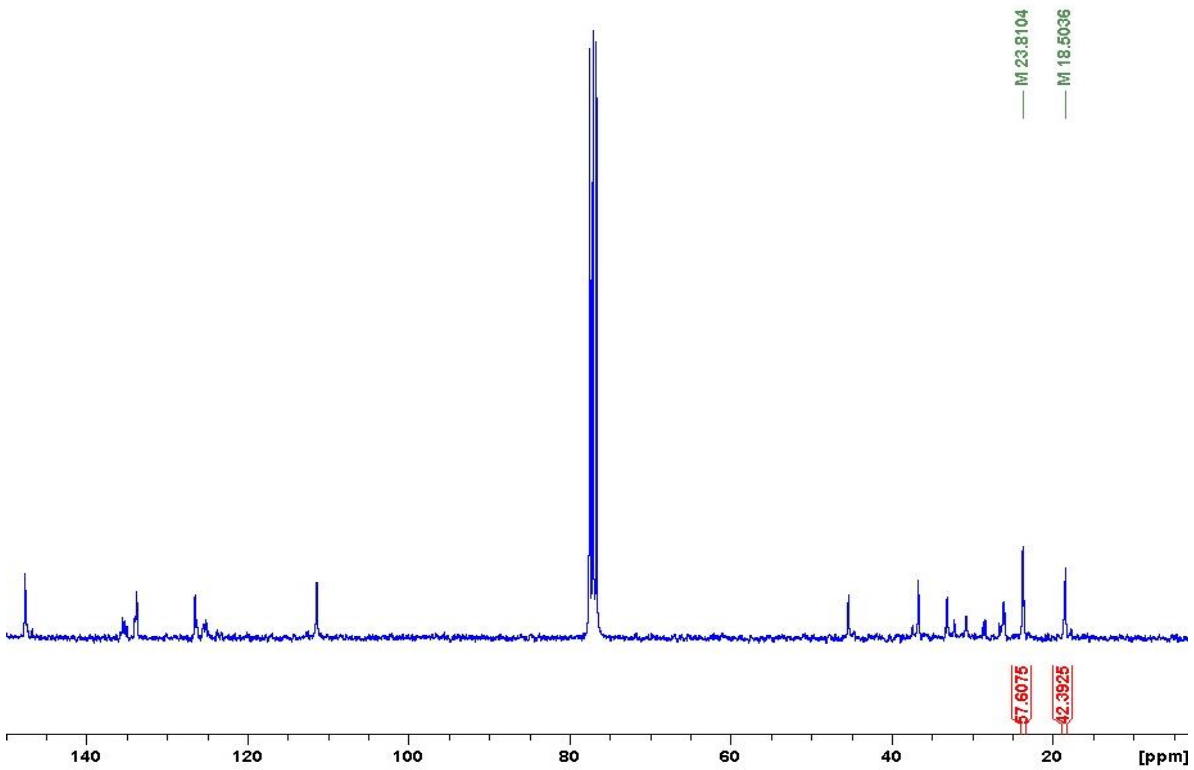

Figure S48. ^{1}H (top) and ^{13}C (bottom) spectra of the polymer obtained with the **Entry 1** of **Table 4**.

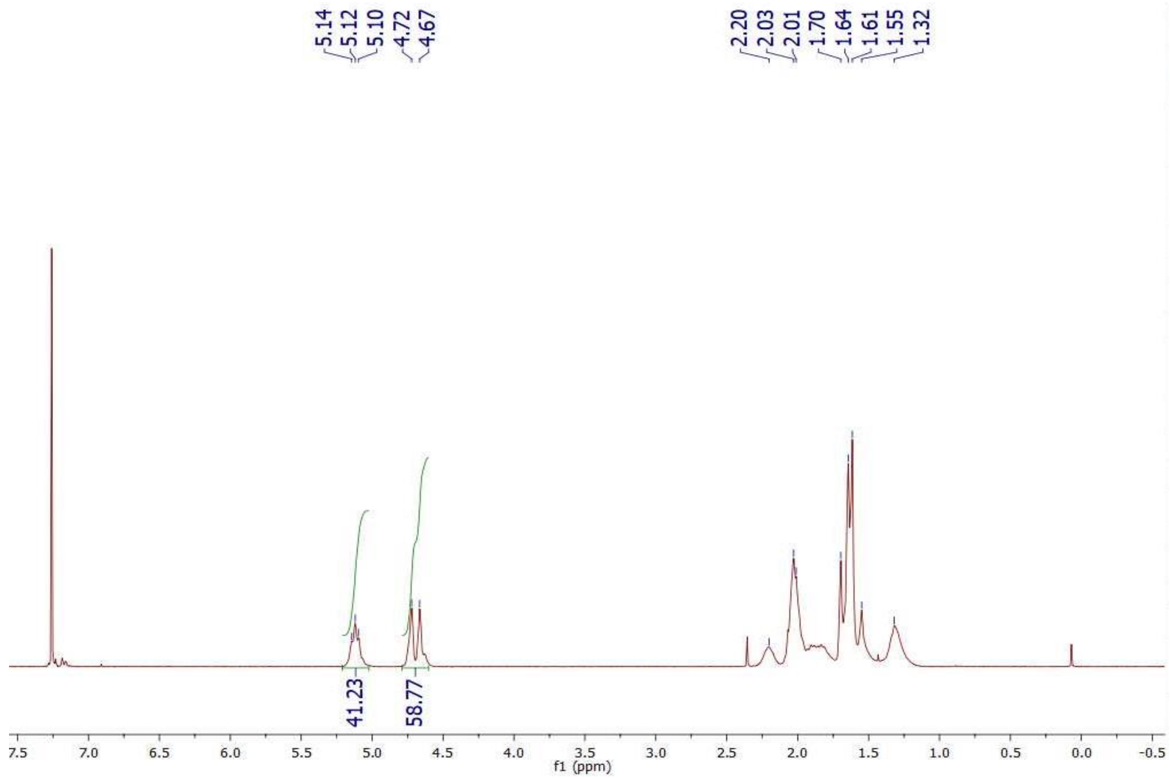

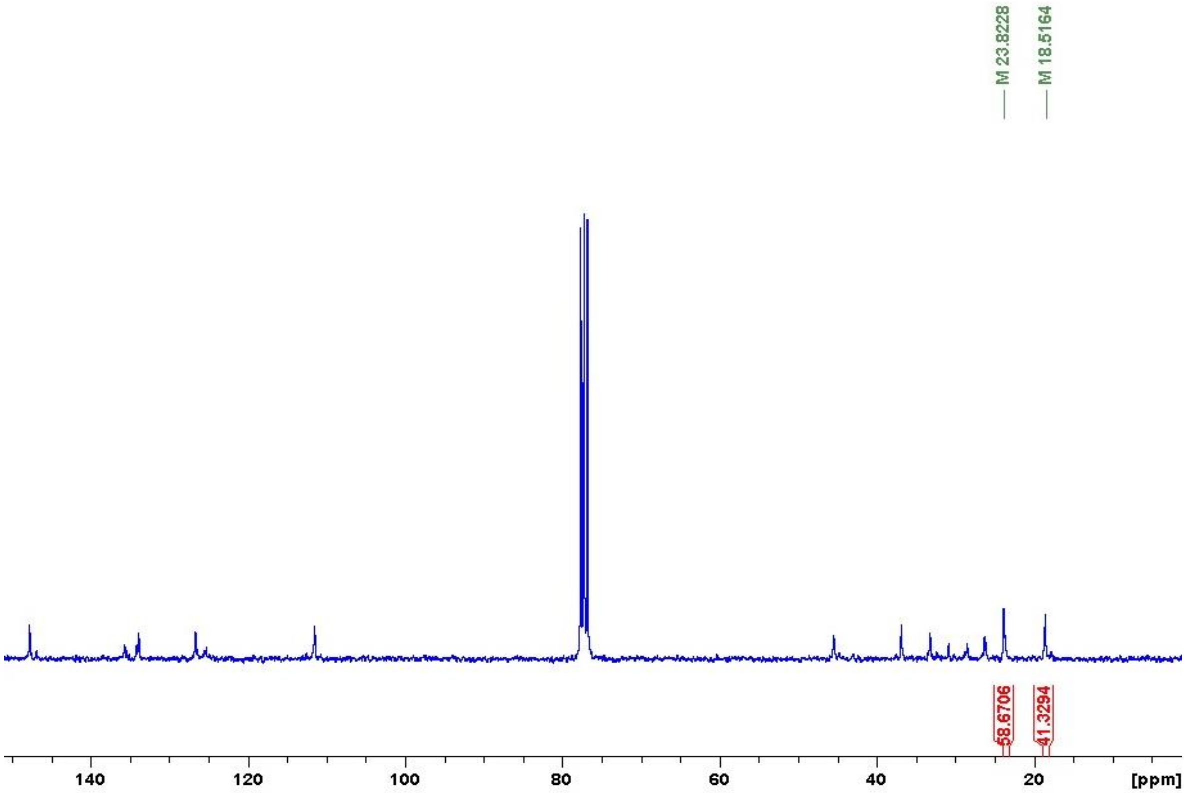

Figure S49. ^{1}H (top) and ^{13}C (bottom) NMR spectra of the polymer obtained with the **Entry 2** of **Table 4**.

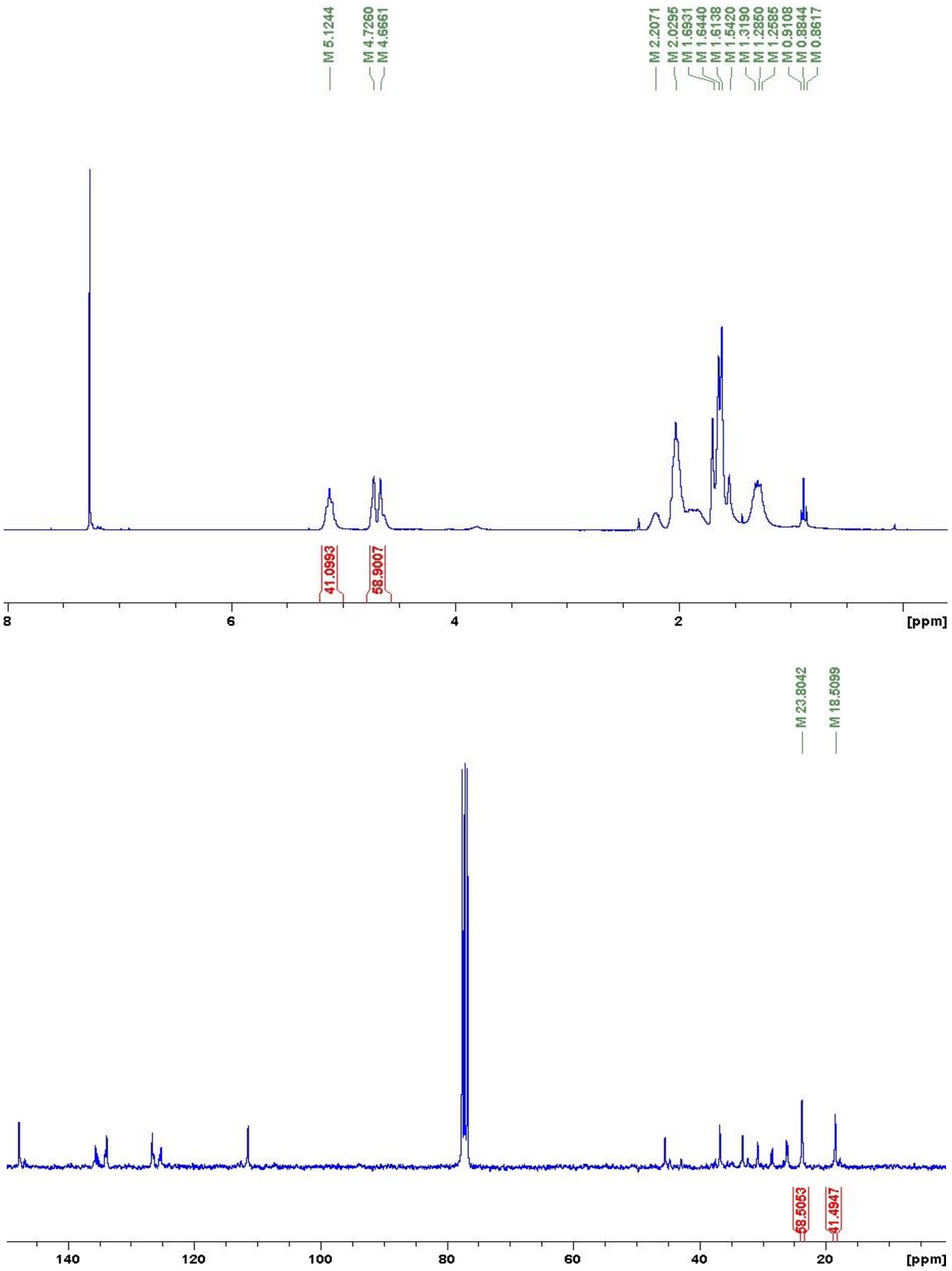

Figure S50. ^{1}H (top) and ^{13}C (bottom) NMR spectra of the polymer obtained with the **Entry 3** of **Table 4**

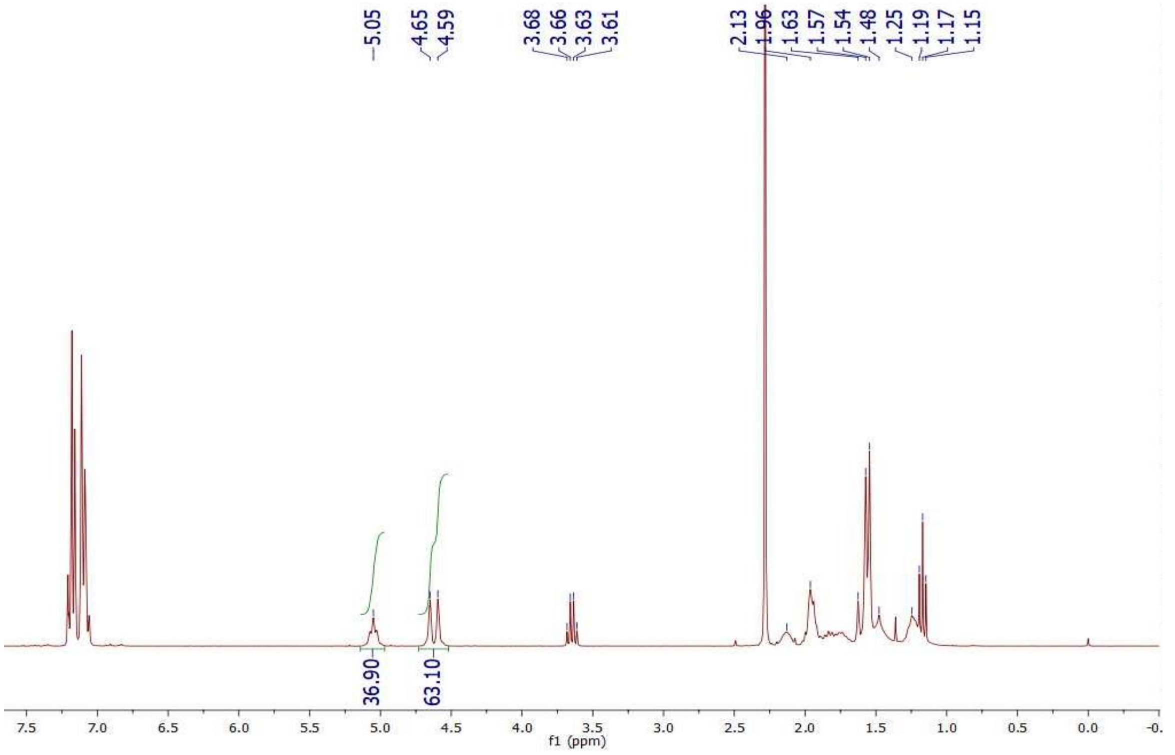

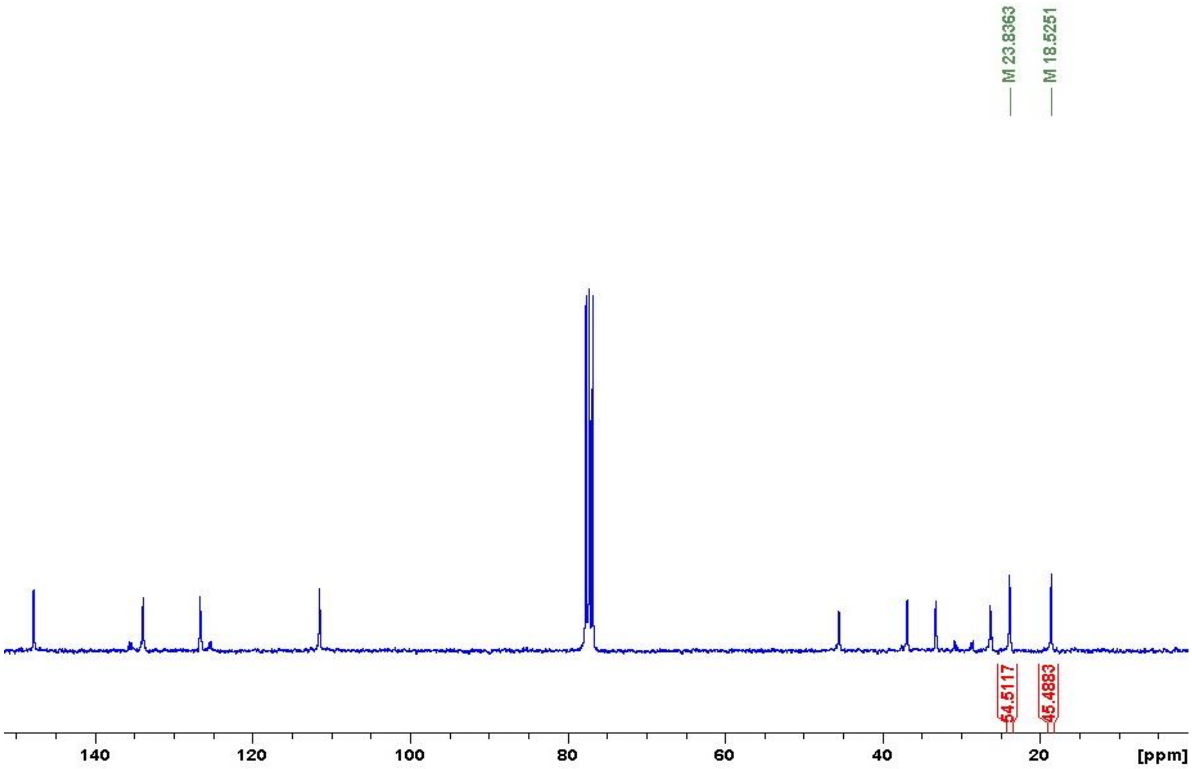

Figure S51. ^{1}H (top) and ^{13}C (bottom) NMR spectra of the polymer obtained with the **Entry 5** of **Table 4**

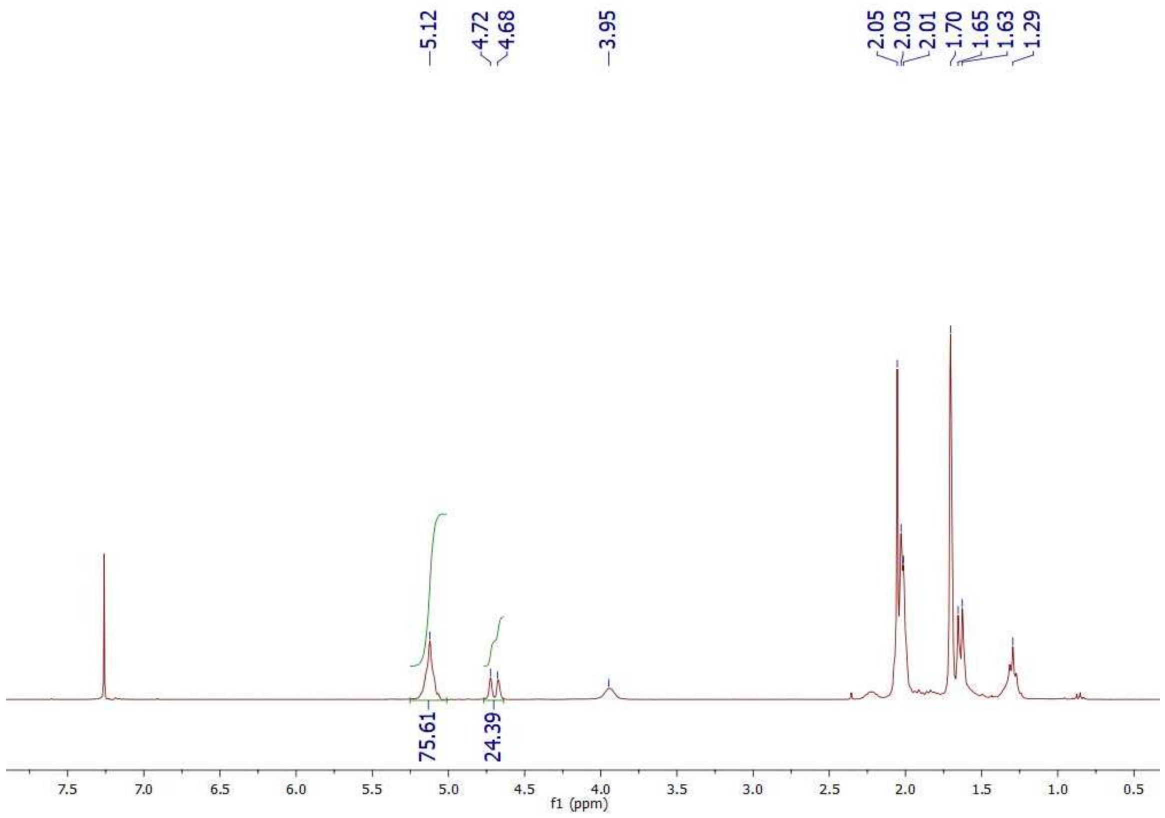

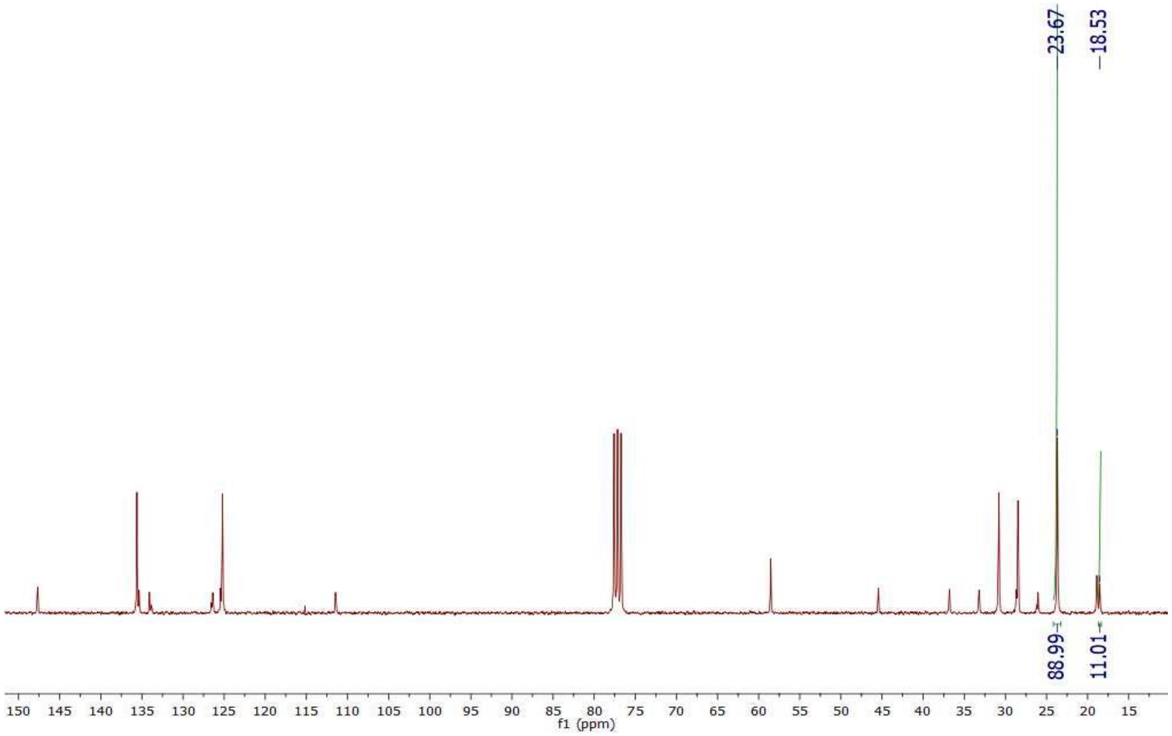

Figure S52. ^{1}H (top) and ^{13}C (bottom) NMR spectra of the polymer obtained with the **Entry 7** of **Table 4**

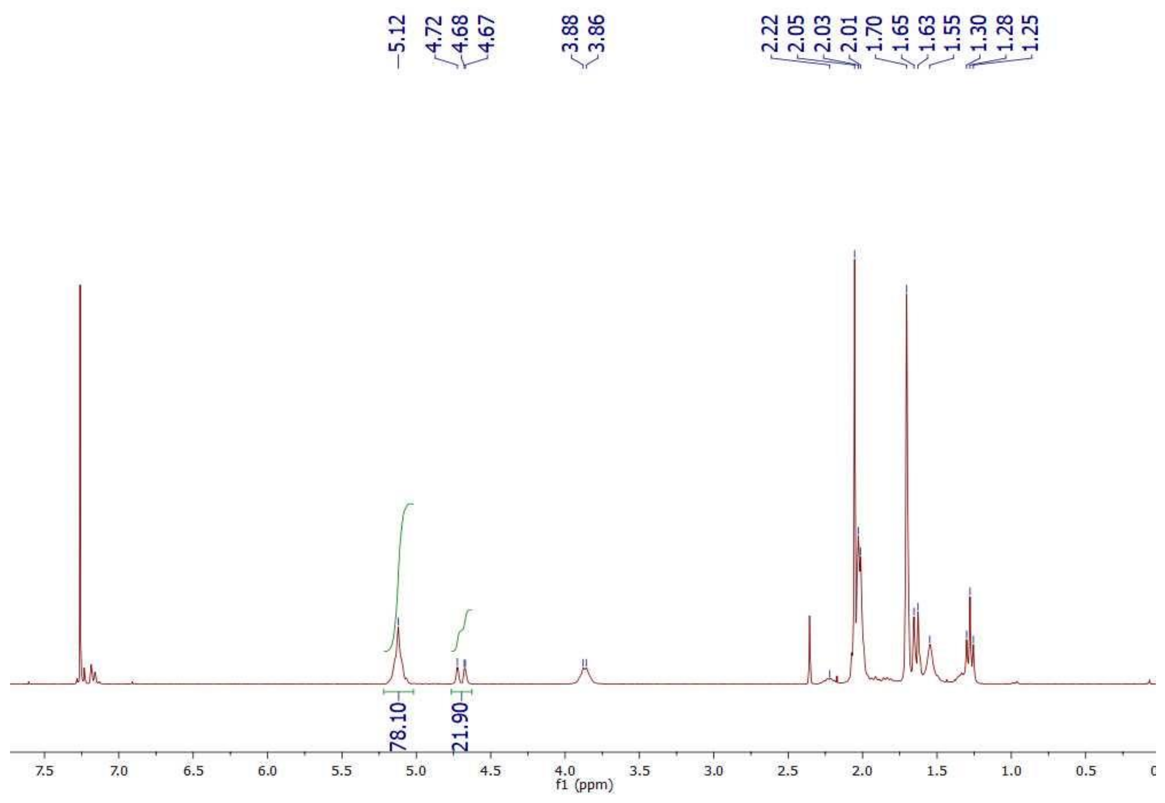

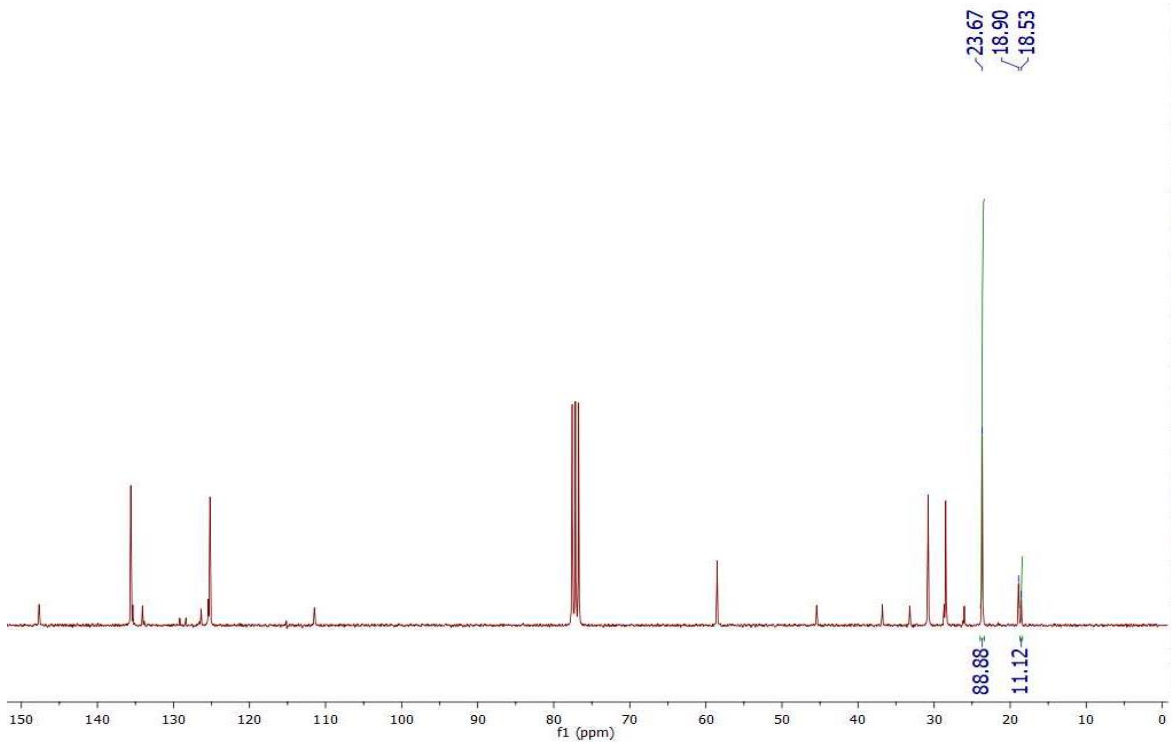

Figure S53. ^{1}H (top) and ^{13}C (bottom) NMR spectra of the polymer obtained with the **Entry 8** of **Table 4**

Figure S54. SEC traces of polymerization experiments

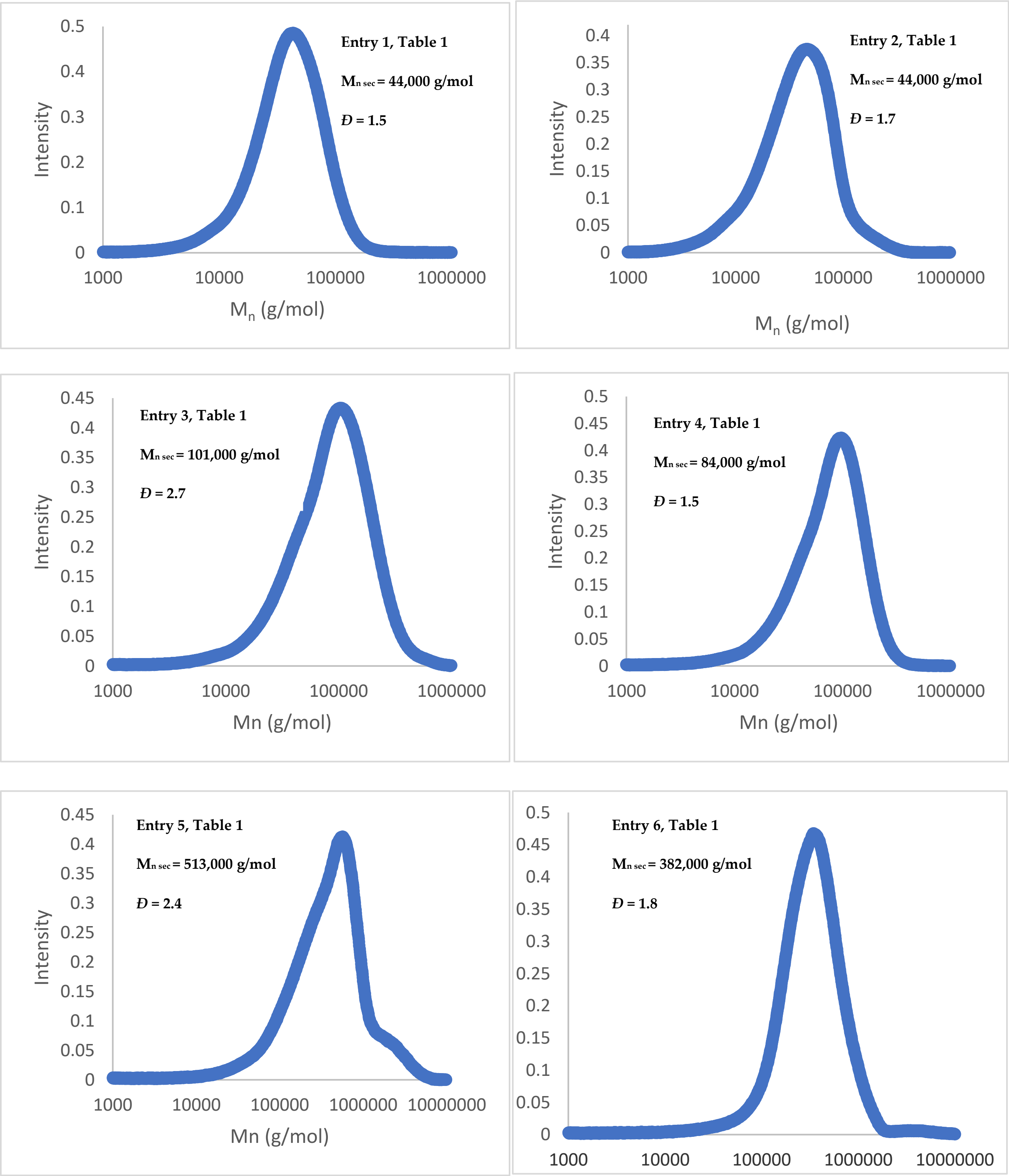

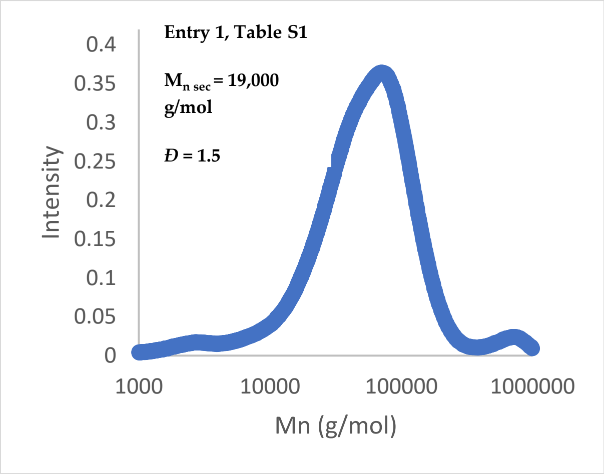
Entry 1, Table S1
Mn sec = 19,000 g/mol
Đ = 1.5
Intensity
Mn (g/mol)

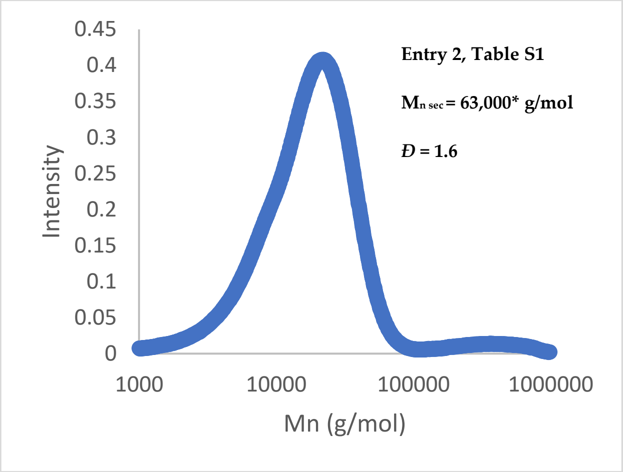
Entry 2, Table S1
Mn sec = 63,000* g/mol
Đ = 1.6
Intensity
Mn (g/mol)

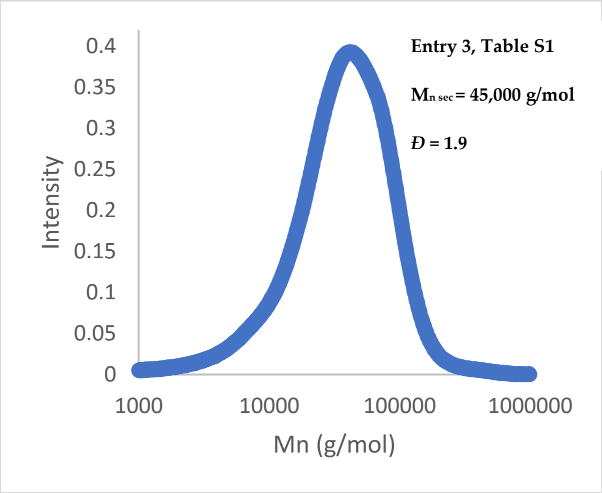
Entry 3, Table S1
Mn sec = 45,000 g/mol
Đ = 1.9
Intensity
Mn (g/mol)

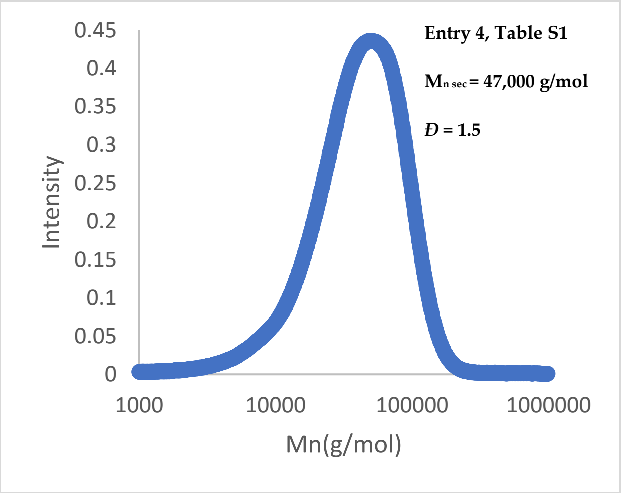
Entry 4, Table S1
Mn sec = 47,000 g/mol
Đ = 1.5
Intensity
Mn(g/mol)

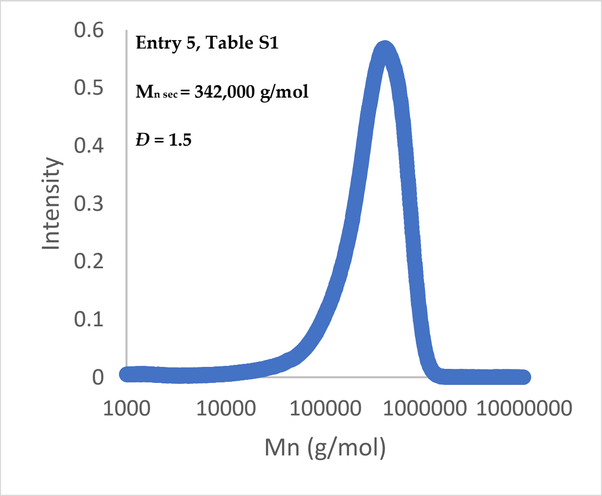
Entry 5, Table S1
Mn sec = 342,000 g/mol
Đ = 1.5
Intensity
Mn (g/mol)

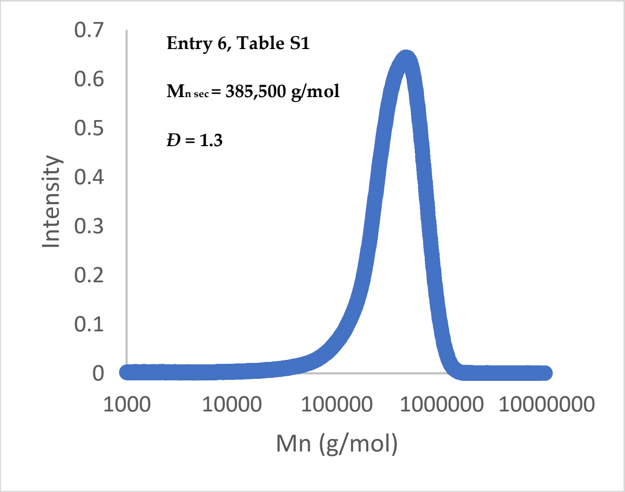
Entry 6, Table S1
Mn sec = 385,500 g/mol
Đ = 1.3
Intensity
Mn (g/mol)

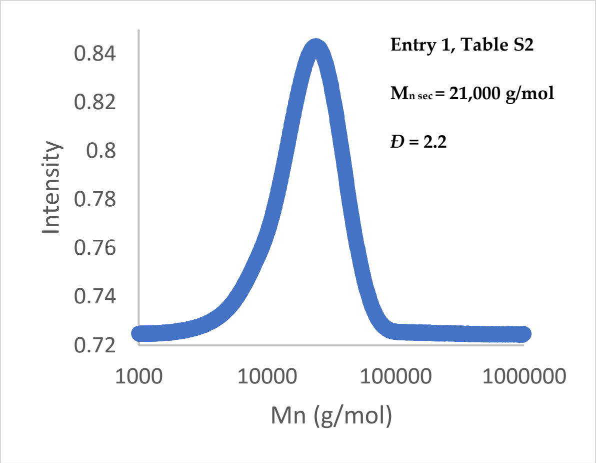
Entry 1, Table S2
$M_{n\ sec}$ = 21,000 g/mol
$Đ$ = 2.2
Intensity
0.84
0.82
0.8
0.78
0.76
0.74
0.72
1000
10000
100000
1000000
Mn (g/mol)

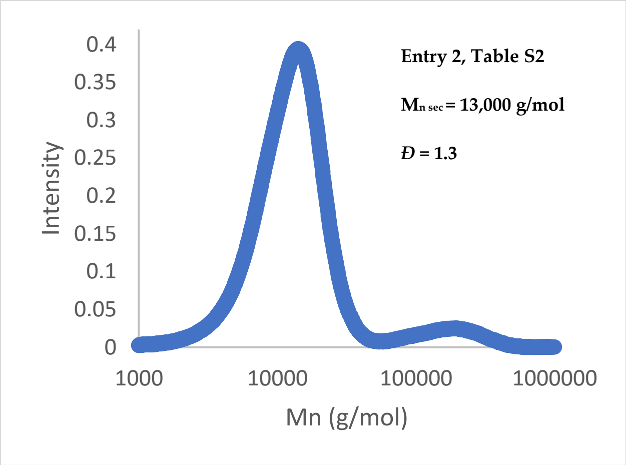
Entry 2, Table S2
$M_{n\ sec}$ = 13,000 g/mol
$Đ$ = 1.3
Intensity
0.4
0.35
0.3
0.25
0.2
0.15
0.1
0.05
0
1000
10000
100000
1000000
Mn (g/mol)

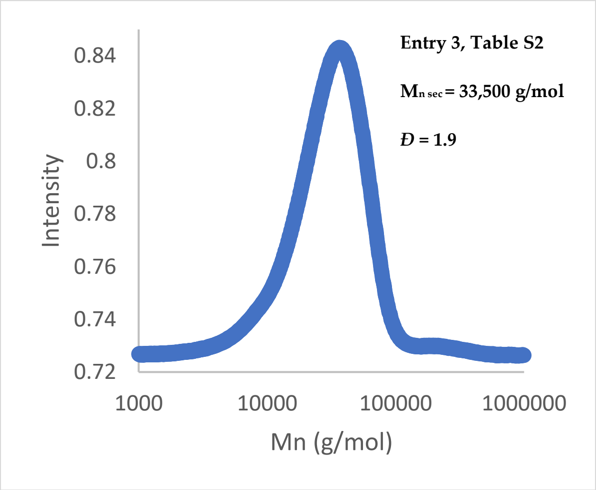
Entry 3, Table S2
$M_{n\ sec}$ = 33,500 g/mol
$Đ$ = 1.9
Intensity
0.84
0.82
0.8
0.78
0.76
0.74
0.72
1000
10000
100000
1000000
Mn (g/mol)

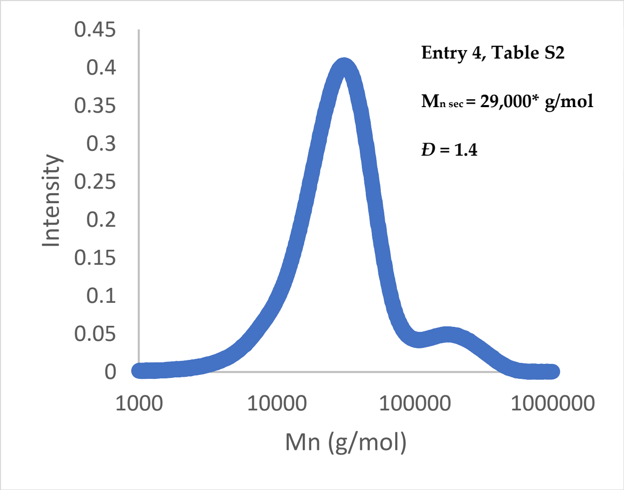
Entry 4, Table S2
$M_{n\ sec}$ = 29,000* g/mol
$Đ$ = 1.4
Intensity
0.45
0.4
0.35
0.3
0.25
0.2
0.15
0.1
0.05
0
1000
10000
100000
1000000
Mn (g/mol)

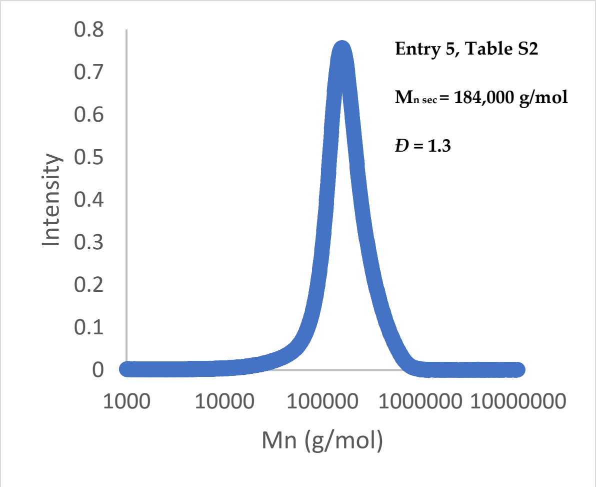
Entry 5, Table S2
$M_{n\ sec}$ = 184,000 g/mol
$Đ$ = 1.3
Intensity
0.8
0.7
0.6
0.5
0.4
0.3
0.2
0.1
0
1000
10000
100000
1000000
10000000
Mn (g/mol)

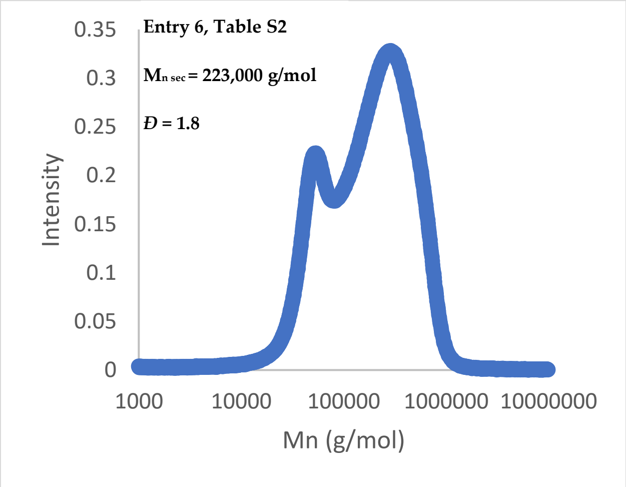
Entry 6, Table S2
$M_{n\ sec}$ = 223,000 g/mol
$Đ$ = 1.8
Intensity
0.35
0.3
0.25
0.2
0.15
0.1
0.05
0
1000
10000
100000
1000000
10000000
Mn (g/mol)

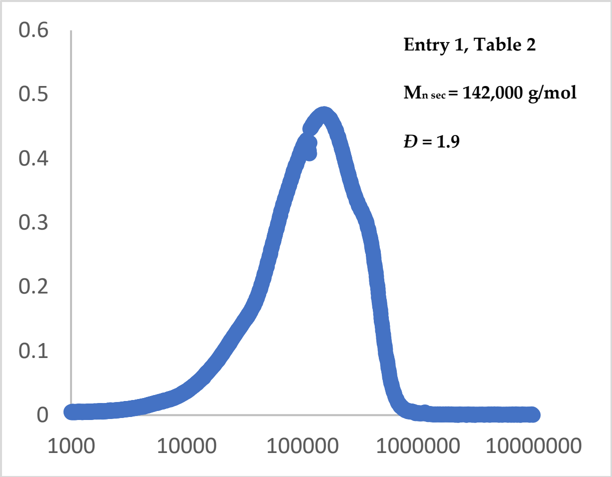
Entry 1, Table 2
$M_{n\ sec}$ = 142,000 g/mol
Đ = 1.9
0.6
0.5
0.4
0.3
0.2
0.1
0
1000
10000
100000
1000000
10000000

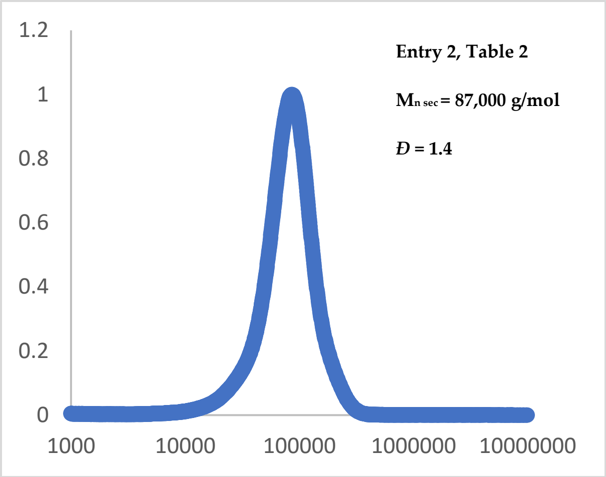
Entry 2, Table 2
$M_{n\ sec}$ = 87,000 g/mol
Đ = 1.4
1.2
1
0.8
0.6
0.4
0.2
0
1000
10000
100000
1000000
10000000

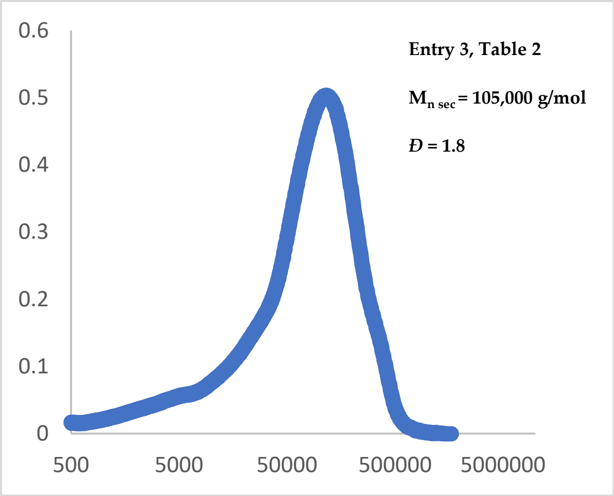
Entry 3, Table 2
$M_{n\ sec}$ = 105,000 g/mol
Đ = 1.8
0.6
0.5
0.4
0.3
0.2
0.1
0
500
5000
50000
500000
5000000

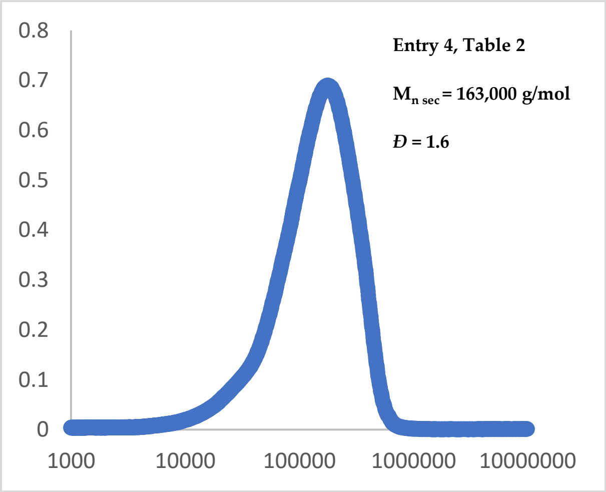
Entry 4, Table 2
$M_{n\ sec}$ = 163,000 g/mol
Đ = 1.6
0.8
0.7
0.6
0.5
0.4
0.3
0.2
0.1
0
1000
10000
100000
1000000
10000000

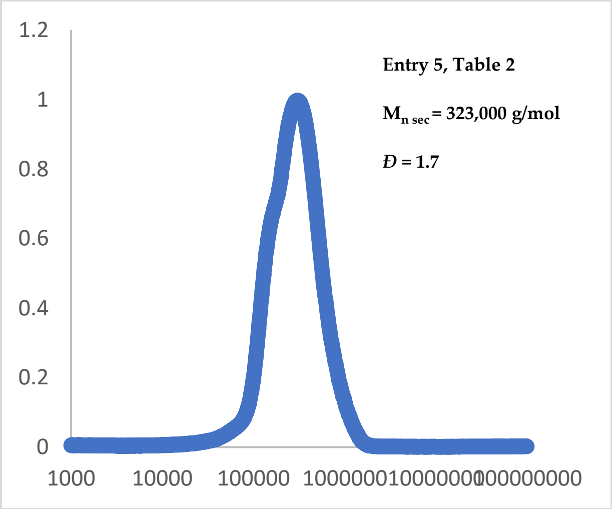
Entry 5, Table 2
$M_{n\ sec}$ = 323,000 g/mol
Đ = 1.7
1.2
1
0.8
0.6
0.4
0.2
0
1000
10000
100000
1000000
10000000
100000000

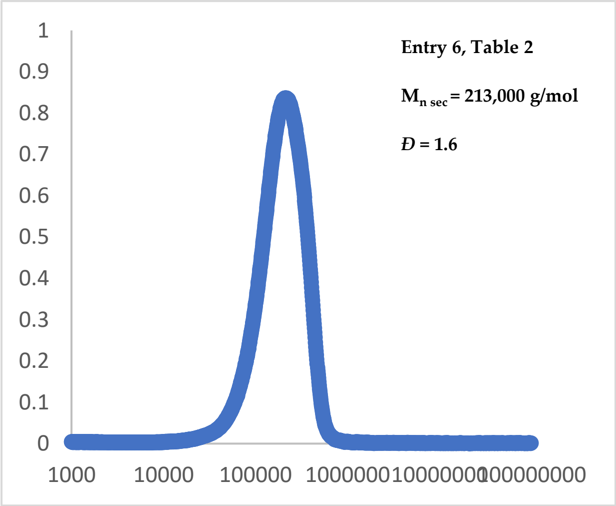
Entry 6, Table 2
$M_{n\ sec}$ = 213,000 g/mol
Đ = 1.6
1
0.9
0.8
0.7
0.6
0.5
0.4
0.3
0.2
0.1
0
1000
10000
100000
1000000
10000000
100000000

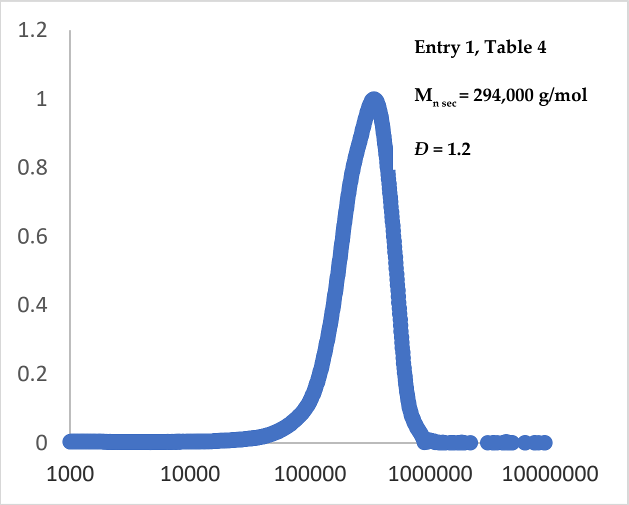
Entry 1, Table 4
M n sec = 294,000 g/mol
Đ = 1.2
1.2
1
0.8
0.6
0.4
0.2
0
1000
10000
100000
1000000
10000000

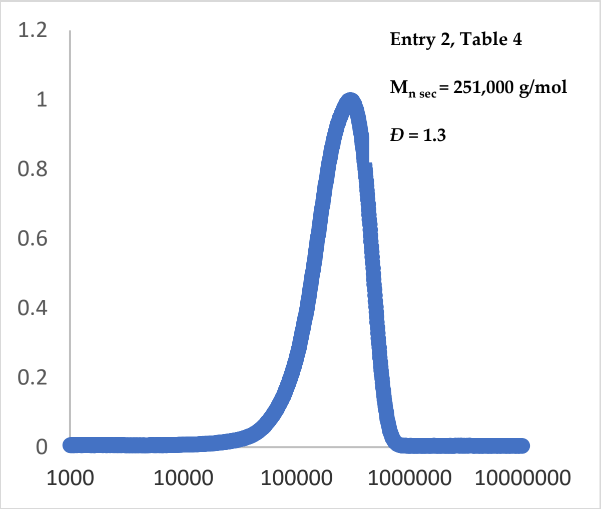
Entry 2, Table 4
M n sec = 251,000 g/mol
Đ = 1.3
1.2
1
0.8
0.6
0.4
0.2
0
1000
10000
100000
1000000
10000000

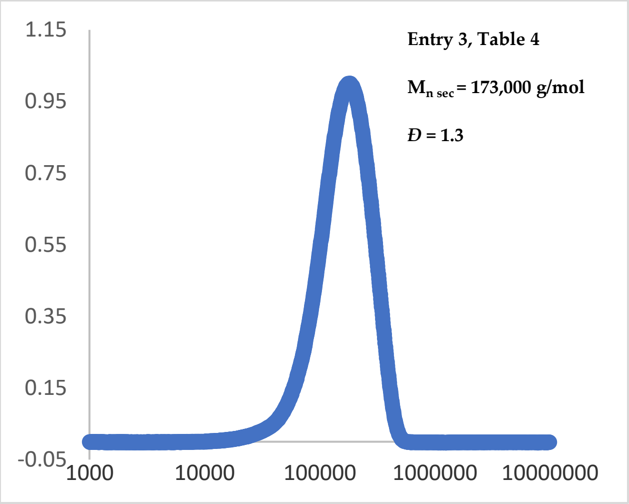
Entry 3, Table 4
M n sec = 173,000 g/mol
Đ = 1.3
1.15
0.95
0.75
0.55
0.35
0.15
-0.05
1000
10000
100000
1000000
10000000

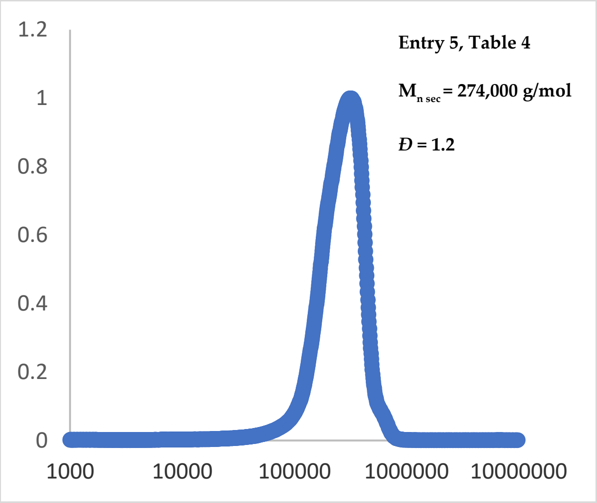
Entry 5, Table 4
M n sec = 274,000 g/mol
Đ = 1.2
1.2
1
0.8
0.6
0.4
0.2
0
1000
10000
100000
1000000
10000000

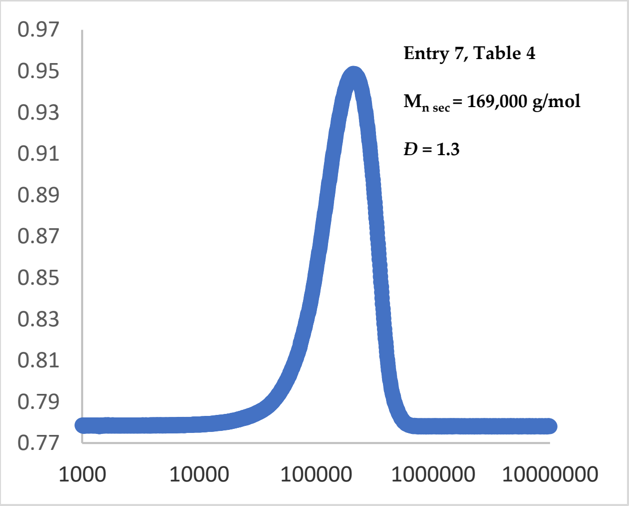
Entry 7, Table 4
M n sec = 169,000 g/mol
Đ = 1.3
0.97
0.95
0.93
0.91
0.89
0.87
0.85
0.83
0.81
0.79
0.77
1000
10000
100000
1000000
10000000

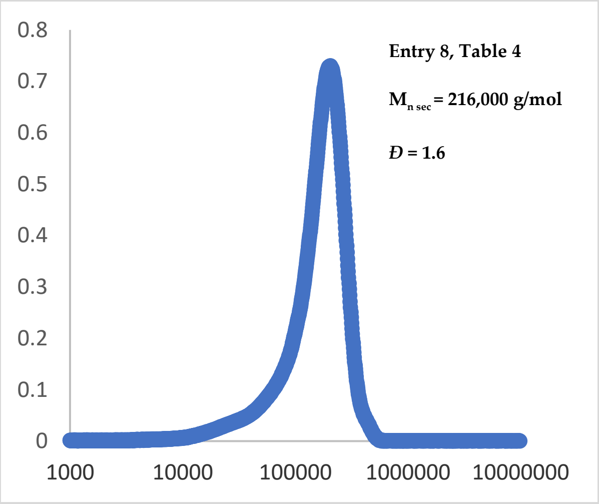
Entry 8, Table 4
M n sec = 216,000 g/mol
Đ = 1.6
0.8
0.7
0.6
0.5
0.4
0.3
0.2
0.1
0
1000
10000
100000
1000000
10000000
